# Supplementary material for: Defining the hidden burden of disease in rural communities in Bangladesh, Cambodia and Thailand: a cross-sectional household health survey protocol
Source: BMJ Open. 2024 Mar 23;14(3):e081079. doi: 10.1136/bmjopen-2023-081079 (PMC10961499; doi:10.1136/bmjopen-2023-081079)
Supplement: Supplementary data [file bmjopen-2023-081079supp001.pdf]

Appendix A. Questionnaire

DEFINING THE HIDDEN BURDEN OF DISEASE IN RURAL COMMUNITIES IN BANGLADESH, CAMBODIA AND THAILAND: A CROSS-SECTIONAL HOUSEHOLD HEALTH SURVEY PROTOCOL

Study site:

Village:

Household ID:

	Household head section		
Code	Question	Answer	Skip logic
S1a	(Household head or adult member of the household) Please give me the names of the persons who usually live in your household starting from the household head.<br><br>*Prompt: Have you told me the names of all the household members, even the ones are not here now?*<br><br>*Record the full name with the study ID in the paper-based household checklist*	Initials, and assign study ID automatically	
S1b	Could you now give me the names of the guests of the household who stayed here last night?		

Zhang M, *et al. BMJ Open* 2024; 14:e081079. doi: 10.1136/bmjopen-2023-081079

S2	What is the relationship of [NAME] to the head of the household?	Head→S3<br>Wife or husband→S4<br>Son or daughter→S4<br>Son-in-law or daughter-in-law→S4<br>Grandchild→S4<br>Parent→S4<br>Parent-in-law→S4<br>Brother or sister→S4<br>Other relative→S4<br>Adopted/ Foster/ Stepchild →S4<br>Not related→S4<br>Don't know→S4	If S2=Head then S3, else skip to S4
S3	What is your ethnic group?	Ethnic group1<br>Ethnic group 2<br>[insert names of locally relevant ethnic groups]	
S4	Is [NAME] male or female?	Male<br>Female	
S5	Does [NAME] usually live here in the past month?	Yes<br>No	
S6	Did [NAME] stay here last night?	Yes<br>No	
S7	How old is [NAME]?	______ in year<br><br>If age 5-14 years→S8-S10<br><br>Else→S1, until completing the information of all the mentioned household members→ S11	Go back to S1 until completing the information of household members.

Zhang M, *et al. BMJ Open* 2024; 14:e081079. doi: 10.1136/bmjopen-2023-081079

S8	Did [NAME] attend school or any early childhood education program at any time during the [2021-2022] school year?	Yes<br>No→S10	If S8=No, then S10
S9	During [this/that] school year, what level [is/was] (NAME) attending?	Early childhood<br>Primary<br>Secondary<br>Higher<br>Don't know<br><br>→ S1, until the information of all household members is taken →S11	
S10	What Is the highest level of school [NAME] has attended?	Early childhood<br>Primary<br>Secondary<br>Higher<br>Don't know<br><br>→ S1, until the information of all household members is taken→ S11	Go back to S1 until completing the list

	**Household Schedule summary**		
Code	Question	Answers	Skip logic
S11	Total persons in household	Auto calculation	
S12	Number of household member >=18 years	Auto calculation	
S13	Number of household member 15-17 years	Auto calculation	
S14	Number of women household member>=15 years	Auto calculation	

S15	Number of school age children 5-14 years old	Auto calculation	
S16	Number of children under 5 years old	Auto calculation	
S17	Just to make sure that I have a complete listing: are there any other people such as small children or infants that we have not listed?	Yes →S1<br>No	If yes, go to S1
S18	Are there any other people who may not be members of your family, such as domestic servants, lodgers, or friends who usually live here?	Yes→S1<br>No	If yes, go to S1
S19	Are there any guests or temporary visitors staying here, or anyone else who stayed here last night, who have not been listed?	Yes→S1<br>No	If yes, go to S1
S20	Is there anybody, who is a household member or a guest who stayed in your household last night, is not here for the survey now?	Yes →S1<br>No →EQ1	
S21	Can you tell me the names of the people who are not here?	Choose from the Initials and study IDs	
S22	What are the reasons [name] is not here now?	Not available at the time (work or school)<br>Physical illness or disability<br>Mental illness or disability<br>Other reason, specify:_____<br>Don't know	

	**Household section**		
Code	Question	Answers	Skip logic
	Now I'm going to ask you some questions about your household amenities and possessions.<br>[use Equity Tool of the country. Example Cambodia]		
	Does your household have		
EQ1	… Electricity	Yes<br>No	
EQ2	… a television?	Yes<br>No	
EQ3	… refrigerator?	Yes	

		No	
EQ4	…. CD or DVD player	Yes<br>No	
EQ5	… wardrobe?	Yes<br>No	
EQ6	…Generator or Battery or Solar panel	Yes<br>No	
EQ7	Does any member of your household own a motorcycle or scooter?	Yes<br>No	
EQ8	… A watch?	Yes<br>No	
EQ9	Does any member of this household have a bank account?	Yes<br>No	
EQ10	What is the main material of the floor?	Ceramic tiles<br>Wood planks<br>Other material	
EQ11	What is the main material of the exterior walls?	Cement blocks<br>Palm / bamboo / thatch<br>Other material	
	**Housing**		
HH1	What is the main source of drinking water for members of your household?<br><br>*(show card)*<br>*The showcard provided the definitions of the water source categories. Based on the description of the respondent, choose from the categories.*	Piped water<br>Piped into dwelling<br>Piped to yard/plot<br>Piped to neighbour<br>Public tap/standpipe<br>Tube well or borehole<br>Dug well<br>Protected well<br>Unprotected well<br>Water from spring<br>Protected spring<br>Unprotected spring	If "bottled water", then HH4

Zhang M, *et al. BMJ Open* 2024; 14:e081079. doi: 10.1136/bmjopen-2023-081079

		Rainwater<br>Tanker truck<br>Cart with small tank<br>Surface water (river/dam/Lake/pond/stream/canal/Irrigation channel)<br>Bottled water→HH4<br><br>Other, specify:____	
HH2	Do you do anything to the water to make it safer to drink?<br><br>*Prompt: Participants might not know it's for making the water safer to drink. The interviewer can prompt if they do anything to the water before drinking.*	Yes<br>No → HH4<br>Don't know	
HH3	What do you usually do to make the water safer to drink? Anything else? (check all that apply)	Boil<br>Add bleach/chlorine<br>Strain through a cloth<br>Use water filter (ceramic/sand/composite/etc)<br>Solar disinfection<br>Let it stand and settle<br>Other, specify______<br>Don't know	
HH4	What kind of toilet facility do members of your household usually use?<br><br>*If the respondent answers in general terms such as "flush toilet," probe to determine where the toilet flushes to; likewise, if the respondent answers "latrine," probe to determine the type of latrine.*	<u>Flush or pour flush toilet</u><br>Flush to piped sewer system<br>Flush to septic tank<br>Flush to pit latrine<br>Flush to somewhere else<br>Flush, don't know where<br><br><u>Pit latrine</u><br>Ventilated improved pit latrine	

	(show card)<br>*The show card provides you definitions of types of toilet, according to the description from the respondent choose the appropriate option.*<br>*If you cannot choose according to the participants description, show them the pictures on the showcard.*	Pit latrine with slab<br>Pit latrine without slab/open pit<br><br>Composting toilet<br>Bucket toilet<br>Hanging toilet/hanging latrine<br>No facility/bush/field<br><br>Other, specify _____________	
HH5	What type of fuel does your household mainly use for cooking?	Electricity<br>Liquefied Petroleum Gas (LPG)<br>Charcoal<br>Wood<br>No food cooked in household<br>Other (specify) ____________________	
HH6	Does the stove have a chimney or fan?	Yes<br>No<br>Don't know	
HH7	Is the cooking usually done in the house, in a separate building, or outdoors?	In the house<br>In a separate building<br>Outdoors<br>Other, specify:______	
HH8	Does your household have any mosquito nets?	Yes<br>No→ HH10	If HH8=No, then HH10
HH9	How many mosquito nets does your household have?	Number of net_______	
HH10	Do all of your household windows have mosquito/fly screens?	Yes<br>No	

Zhang M, *et al. BMJ Open* 2024; 14:e081079. doi: 10.1136/bmjopen-2023-081079

ST1	How often is your house floor being cleaned (e.g. swept)?	Everyday (7 days/week)<br>Almost everyday (5-6 days/week)<br>Every other day (3-4 days/week)<br>Weekly (1-2 days/week)<br>Monthly (1-3 days/month)<br>Scarcely (a few times/ year)<br>Never	
ST2	How often do you or other members of your household perform activities to maintain grass or weed in or around your household, or work on a vegetable garden?<br><br>Prompt: For example, cut or trim weeds, plant or pick vegetables in the yard, garden, by the walls or fences of your household?	Everyday (7 days/week)<br>Almost everyday (5-6 days/week)<br>Every other day (3-4 days/week)<br>Weekly (1-2 days/week)<br>Monthly (1-3 days/month)<br>Scarcely (a few times/ year)<br>Never	
	**Household member check**		
	Now I'm going to asking you some questions about the members of your household including you, if anyone have had an accident, or had illness in the past 30 days.		
	**Accident and illness in the past 30 days**		
HM1	Was any person of your household injured or killed in an accident?<br>Prompt: anyone injured or killed in an accident any time before the interview.	Yes, how many? ___<br>No→HM14	If HM1=No, then HM14
HM2	What the name of the person got injured or killed in an accident?	Identify the ID code and show initial<br>Not in the list, initial:____	
HM3	Could you tell me in what type of accident (NAME) was injured or killed?	Landmine/unexploded bomb (UXO)<br>Gunshot/weapon<br>Road accident<br>Fire/burning<br>Snake/animal bite<br>Fall from tree/building<br>Drowning	

Zhang M, *et al. BMJ Open* 2024; 14:e081079. doi: 10.1136/bmjopen-2023-081079

		Poisoning (chemical)<br>Violence/assault<br>Other, specify________<br>Don't know	
HM4	Is (NAME) still alive?	Yes<br>No→HM6	If HM4 =No, then HM6
HM5	In your opinion, was (NAME'S) injury serious, moderate, or slight?	Serious<br>Moderate<br>Slight<br>DON'T KNOW	
HM6	Was (NAME's) death due to accident?	Yes<br>No → HM7	→HM7
HM7	Did the accident happen in the past 30 days?	Yes<br>No→HM14	If No then HM 14
HM8	Was advice or treatment sought for [Name] after the accident?	Yes<br>No→ HM14	If No then HM14
HM9	Where was advice or treatment first sought for [NAME] after the accident?	Public sector<br>Primary care unit/Primary care centers/Community Health centers<br>Community hospital<br>General hospital/Central hospital<br>University hospital<br>Government hospital<br>Private medical sector<br>Poly private clinic/private clinic<br>Private hospital<br>Others<br>source<br>Traditional/herbal medicine<br>Traditional healer<br>Self-bought medicine<br>Relatives/ Friends<br>, specify:________	

Zhang M, et al. BMJ Open 2024; 14:e081079. doi: 10.1136/bmjopen-2023-081079

HM10	Where did the money come from to pay for transportation and treatment for the members of your household who had an injury over the past 30 days?<br><br>Record all that mentioned<br><br>*The payment method for transportation and medical care can be different. If the respond provided one method, the interviewer should probe and confirm if both payment for transportation and treatment are from the same method.*<br><br>*If any out-of-pocket payment method is selected, then proceed to HM11.*	Subsidy or exemption→ HM14<br>Insurance scheme →HM14<br>Universal coverage card (UC)<br>Social Security/ worker compensated fund (SSSS)<br>civil servant medical benefit scheme (CSMBS)<br>State enterprise<br>Independent agency of the state<br>Local government<br>Private health insurance<br>Health insurance covered by employer<br>Other health insurance, specify____________<br><br>Out of pocket → HM11<br>Wage/income<br>Loan/borrowed money<br>Sale of assets<br>Gift from relatives/neighbours/friends<br>Saving/ family funds<br><br>Other, specify________→HM14<br>Don't know→HM14	
HM11	How much in total was spent on [NAME]'s treatment at (name of the place)?	_______amount of money<br>Don't know	If HM10 checked out of pocket payment.
HM12	How much in total was spent on transport to go to and return from (name of the place)?	_____amount of money<br>Don't know	If HM10 checked out of pocket payment.
HM13	Has the debt been repaid?<br>*(If HM10= Loan/borrowed money)*	Yes<br>No<br>Don't know	If HM10= Loan/borrowed money
HM14	Please tell me if any member of your household (including you) is sick, has an illness now or at any time in the last 30 days?	Yes, how many? ________<br>No → End of the household head section	If HM14=No, then end of the section

Zhang M, *et al. BMJ Open* 2024; 14:e081079. doi: 10.1136/bmjopen-2023-081079

	It can be **acute** disease or previous diagnosed **chronic** disease.<br>An acute illness is a condition that appears suddenly: the person did not have it immediately before becoming ill.<br>A chronic disease is an illness that will not go away or takes a long time to go away, even when treated.<br><br>Prompt if nobody:<br>Is there anyone had fever?<br>Any infant or child in your family was ill?		
HM15	Now I would like to ask you some questions about each person who is sick now or at any time in the <u>last 30 days</u>.<br><br>Could you tell me his/her/their name(s)? Then we will talk about one person at a time.	Identify all that from the list of the family member (ID number)<br><br>No body→ end of the household head section	
HM16	What type of health problems or symptoms did [name] have or is having during this illness?<br><br>DO NOT READ. TICK ONE BOX FOR EACH GROUP OF SYMPTOMS MENTIONED.<br><br>*If the respondent provide a diagnosis directly, especially for an acute condition, please still probe for the symptoms. Check all the symptoms that apply, and check "other acute condition, specify: ____ and write down the diagnosis.*<br>*For example: if the respondent answer " Covid", check "other acute condition, specify: COVID". Then continue probe what did the symptoms the person have, and check all the symptoms that are described.*	<u>Acute conditions</u><br>Fever<br>Persistent headaches<br>Persistent cough<br>Running nose<br>Difficulty breathing, fast breathing<br>Difficulty swallowing/ throat pain<br>Difficulty seeing or other eye complaint<br>Stomach pain, nausea, vomiting, could not eat<br>Thirst/ sweating<br>Watery diarrhoea<br>Bloody stools<br>Skin disease, such as rush, irritation, open sores<br>gyn-obstetrics<br>Could not sleep<br>Feeling tired<br>Constipation<br>Convulsion/ seizure	

Zhang M, *et al. BMJ Open* 2024; 14:e081079. doi: 10.1136/bmjopen-2023-081079

		Musculoskeletal pain (general body pain, back and joint pain)<br><br><u>Chronic disease</u><br>Hypertension, high blood pressure<br>Heart disease, heart attack consequence<br>Diabetes, high blood sugar<br>Asthma, wheezing, chronic difficulty breathing<br>HIV infection, AIDS<br>Arthritis, chronic body pain<br>High cholesterol<br>Ulcer, chronic stomach pain<br>Stroke consequence<br>Epilepsy, seizures, fits<br>Cancer<br>Tuberculosis<br>Liver disease<br>Depression<br><br>Other acute illness, specify: ______<br>Other chronic illness, specify:_______<br>Don't know	
HM17	In your opinion, was [NAME]'s illness serious, moderate, or slight?	Serious<br>Moderate<br>Slight<br>Don't know	
HM18	Was advice or treatment sought for [Name]'s illness?	Yes<br>No→End of the household head section	If HM18=No, then end of the section
HM19	Where was advice or treatment first sought for [NAME]'s illness?	<u>Public sector</u><br>Government hospital<br>Government health centre<br>Government health post	

	[insert Country specific list]	Mobile clinic<br>Community health worker/field worker<br>Other public sector<br>Private medical sector<br>Private hospital<br>Private clinic<br>Pharmacy<br>Private doctor<br>Mobile clinic<br>Community health worker/field worker<br>Other private medical sector<br>NGO medical sector<br>NGO hospital<br>NGO clinic<br>Other NGO medical<br><br>Other source<br>Shop<br>Traditional practitioner<br>Market<br>Itinerant drug seller<br><br>Other, specify:________	
HM20	Where did the money come from to pay for transportation and medical care for the members of your household who had an illness over the past 30 days? (record all that mentioned)<br><br>*The payment for transportation and medical care can be different. If the respond provided one method, the interviewer should probe and confirm if both payment for transportation and treatment are from the same method.*	Subsidy or exemption→end of the section<br><br>Insurance scheme→ end of the section<br>Mutual Health Organization/Community-Based Health Insurance<br>Health Insurance through Employer<br>Social Security<br><br><br>Out of pocket → HM21<br>Wage/income	

	If any out-of-pocket method is recorded, proceed to HM21.<br><br>[Insert locally relevant insurance schemes]	Loan/borrowed money<br>Sale of assets<br>Gift from relatives/neighbours/friends<br>Saving/ family funds<br>specify__________--> End of the household head section<br>Don't know → End of the household head section	
HM21	How much in total was spent on [NAME]'s treatment at (name of the place)?	_______amount of money<br>Don't know	If HM20 checked any out of pocket payment.
HM22	How much in total was spent on transport to go to and return from (name of the place)?	_____amount of money<br>Don't know<br><br>➔ HM23, if HM20=loan/borrowed money.<br>➔ Else, end of the household head section	HM23, if HM20=loan/borrowed money.<br>Else, end of the household head section
HM23	Have this debt been repaid?	Yes<br>No<br>Don't know	If HM20=Loan/borrowed money

Zhang M, *et al. BMJ Open* 2024; 14:e081079. doi: 10.1136/bmjopen-2023-081079

	Adult section		
Code	Question	Answers	Skip logic
	**Respondent background**		
A1	How old were you at your last birthday?	Age in complete years __	
A2	Have you ever attended school?	Yes<br>No → A4	If no, then A4
A3	What is the highest level of school you completed?	No formal schooling<br>Less than primary school<br>Primary school uncompleted<br>Primary school completed<br>Secondary school completed<br>High school/ vocational school completed<br>College/university completed<br>Postgraduate degree	
A4	What is your marital status?	Never married<br>Currently married<br>Separated<br>Divorced<br>Widowed<br>Cohabitating<br>Refuse to answer	

Zhang M, *et al. BMJ Open* 2024; 14:e081079. doi: 10.1136/bmjopen-2023-081079

A5	Which of the following best describes your main work status over the past 12 months?	Government employee<br>Non-government employee<br>State enterprise officer<br>Private company employee<br><br>Business<br>Business owner<br>Household business assistance<br>Agriculture<br>Agriculture (landowner and farmer)<br>Agriculture labourer (other's land)<br>Labourer<br>Industrial labourer<br>Day labourer<br>Transport labourer<br>Paid domestic worker<br>Blacksmith/ Goldsmith/other skilled labour<br>Other self-employed<br>Student<br>Home maker/household work<br>Retired<br>Unemployed<br>Unemployed (able to work, e.g. unwilling to work)<br>Unemployed (unable to work, e.g. too sick, disabled)<br><br>Other, specify: ________<br>Refuse to answer	
	Now, I'm going to ask you some questions about your behaviour when you are in or around your household, and when you are working, in the past 12 months.		
ST3	In the past 12 months, how often do you sit or lying directly on the floor in your house or lawn?	Everyday (7 days/week)<br>Almost everyday (5-6 days/week)	

		Every other day (3-4 days/week)<br>Weekly (1-2 days/week)<br>Monthly (1-3 days/month)<br>Scarcely (a few times/ year)<br>Never	
ST4	In the past 12 months, have you worked in forest or high hill (for collecting wood/ bamboo/ mushroom), or rice field, fruit/tea/coffee plantation, or dry farm field for other agriculture products (e.g. vegetable, corn, pineapple)?<br><br>*Prompt: the participant might have worked in different areas throughout the year, and worked different length in different area, prompt to record all areas that the participate have worked in the past 12 months.*	Yes<br>No→WHS1	If no, then WHS1
ST5	Which one of them have you worked in?<br>*Check all that apply*	Forest or high hill<br>Rice field<br>Fruit/Coffee/tea plantation<br>Dry field	Completing ST6-ST8 for each area checked
ST6	Do you work in the [areas checked above] all year or only when in some seasons?<br><br>*Check "all year" if worked every month in the past 12 months. The respondent might work with different frequency in different month, but if he/she has worked in the area in each month in the past 12 month, this option should be checked.*<br>*Check "in season" if not every month in the past 12 month.*	All year→ST8<br>In season →ST7	

ST7	How many months have you worked in [area] in the past 12 months?<br><br>*The number of months should be cumulative including all the months in the past 12months the respondent has worked in the area. E.g. if the participants worked in March-June, and November, the total months he/she worked in the area is 4 months, "3-5 months" should be checked.*	9-11 months<br>6-8 months/year<br>3-5 months/year<br>1-2 months/year or less	IfST6= In season
ST8	How often do you work in [area] (when it was the months you worked there)?<br><br>*Ask the question accordingly: if the respondent answered only work in the area in some seasons, then read the words in "(when it was the months you worked there)". If different frequency in different month, please use an average.*	Everyday (7 days/week)<br>Almost everyday (5-6 days/week)<br>Every other day (3-4 days/week)<br>Weekly (1-2 days/week)<br>Monthly (1-3 days/month)<br>Scarcely (a few times/ year)<br><br>➔ ST6, until completed all the areas the participants have listed	ST6 until completed the frequency for all the fields the respondent has worked in the past 12 months.
ST9a	The following questions we will ask you some things you do when you were at work in the area(s) you mentioned before. When you answer them, you need to consider: (all)the area(s) you have mentioned, and the total time you have worked in those area(s) in the past 12 months.<br><br>*Repeat the area(s) and the frequencies of working in the area from ST5-ST8*	Show the area(s) and frequency from ST5-ST8	
ST9	At the time when you work in the area(s), how often do you sit or lay directly on the ground, haystacks, grass or other places in the forest or high hill, or farm field without a mat?	Every time<br>Most times<br>Sometimes<br>Rarely<br>Never	

Zhang M, *et al. BMJ Open* 2024; 14:e081079. doi: 10.1136/bmjopen-2023-081079

ST10	How often do you wear long sleeves?	Every time<br>Most times<br>Sometimes<br>Rarely<br>Never	
ST11	How often do you wear gloves?	Every time<br>Most of times<br>Sometimes<br>Rarely<br>Never	
ST12	How often do you wear shoes with socks or boots without or without socks?	Every time<br>Most of times<br>Sometimes<br>Rarely<br>Never	
ST13	How often do you wash your body after work?	Every time<br>Most of times<br>Sometimes<br>Rarely<br>Never	
ST14	How often do you change your clothes wore at work to clean clothes immediately (i.e., within 30 minutes) when you arrive home?	Every time<br>Most of times<br>Sometimes<br>Rarely<br>Never	
WHS1	I'm going to ask you now your overall health, including both your physical and your mental health.<br><br>In general, how would you rate your health today? Would you say it's very good, good, moderate, bad, or very bad?	Very good<br>Good<br>Moderate<br>Bad<br>Very Bad	

Zhang M, *et al. BMJ Open* 2024; 14:e081079. doi: 10.1136/bmjopen-2023-081079

WHS2	The following questions, I'm going to ask you how much difficulty you have when doing some activities. You can give me answers, such as you have no difficulty, mild difficulty, moderate difficulty, or extreme difficulty, or cannot do it at all.<br><br>Overall in the last 30 days, how much difficulty did you have with work or household activities?	None<br>Mild<br>Moderate<br>Severe<br>Extreme/ Cannot do	
WHS3	Overall in the last 30 days, how much difficulty did you have with moving around, such as walking or climbing steps	None<br>Mild<br>Moderate<br>Severe<br>Extreme/ Cannot do	
WHS4	Overall in the last 30 days, how much difficulty did you have with selfcare, such as washing or dressing yourself?	None<br>Mild<br>Moderate<br>Severe<br>Extreme/ Cannot do	
WHS5	Overall in the last 30 days, how much of bodily aches or pains did you have?	None<br>Mild<br>Moderate<br>Severe<br>Extreme	
WHS6	Overall in the last 30 days, how much difficulty did you have with concentrating or remembering things?	None<br>Mild<br>Moderate<br>Severe<br>Extreme	
WHS7	Do you wear glasses or contact lenses?	Yes<br>No	

Zhang M, *et al. BMJ Open* 2024; 14:e081079. doi: 10.1136/bmjopen-2023-081079

WHS8	In the last 30 days, how much difficulty did you have in seeing (IF WHS7=Yes, even when you wear glasses or contact lenses)?	None<br>Mild<br>Moderate<br>Severe<br>Extreme	
WHS9	Overall in the last 30 days, how much of a problem did you have with sleeping, such as falling asleep, waking up frequently during the night or<br>waking up too early in the morning?	None<br>Mild<br>Moderate<br>Severe<br>Extreme	
WHS10	Overall in the last 30 days, how much of a problem did you have with feeling sad, low, or depressed?	None<br>Mild<br>Moderate<br>Severe<br>Extreme	
	**Substance use**		
SU1	Now I would like to ask you some questions on smoking and tobacco use.<br>Do you currently smoke any tobacco products, such as cigarettes, cigar, or pipes?	Yes<br>No→S6	If No, then S6
SU2	Do you currently smoke tobacco product daily?	Yes<br>No	
SU3	How old were you when you first started smoking?	Age ______years→ S5<br>Don't know→ S4	If S3=Don't know, then S4, else S5
SU4	Do you remember how long ago it was?	__________in year/month/week<br>Don't know	
SU5	What type of tobacco do you currently smoke?<br>(Check all that apply)	Manufactured cigarettes<br>Hand-rolled cigarettes<br>Kreteks<br>Pipes full of tobacco<br>Cigars, cheroots, or cigarillos<br>Water pipe (e.g. Baraku/ Hookah/ Shisha)<br>Other, specify:____	Jump to S10

Zhang M, *et al. BMJ Open* 2024; 14:e081079. doi: 10.1136/bmjopen-2023-081079

		→ Select any option, move to S10	
SU6	In the past, did you ever smoke any tobacco products?	Yes<br>No→ S10	If S6=No, then S10
SU7	In the past, did you ever smoke tobacco product daily?	Yes<br>No	
SU8	How old were you when you first stopped smoking	Age ______years→S10<br>Don't know→S9	If S8=Don't know, then S9, else S10
SU9	Do you remember how long ago it was?	__________in year/month/week<br>Don't know	
SU10	Now I would like to ask you some questions about drinking alcohol.<br>Have you ever consumed any alcohol, such as beer, wine, spirits, or [ADD OTHER LOCAL EXAMPLES]?	Yes<br>No→S18	If No, then S18
SU11	We count one drink of alcohol as one can or small bottle of beer, one glass of wine, one shot of spirits, or [insert local example].<br><br>(Show card)<br><br>During the past 12 months, how frequently have you had at least one standard alcoholic drink?<br><br>*Refer to locally relevant examples of one standard drink.*	Daily<br>5-6 days per week<br>3-4 days per week<br>1-2 days per week<br>1-3 days per month<br>Less than once a month<br>Never	If S11=Never, then AS18
SU12	Have you consumed any alcohol in the past 30 days?	Yes<br>No→S18	If S12=No then S18
SU13	During the past 30 days, on how many occasions did you have at least one standard alcoholic drink?	Number_____<br>Don't know	

SU14	In the past 30 days, how many standard drinks on average did you have during one drinking occasion?<br><br>(Showcard)<br>*If the respondent is not able to reply by standard drink, ask on average the amount and type of alcohol he/she consume, and convert by yourself to number of standard drinks.*	Number of drinks_____<br>Don't know	
SU15	In the past 30 days, how many occasions did you have six or more standard alcoholic drinks in a single drinking occasion?<br><br>(Showcard)<br><br>*Prompt: Six standard drinks is roughly about: 6 cans of beer/3 big bottles of beer/ 6 shots of spirit/ a bottle of spirit shared among 3-4 people [insert local examples].*	Number of occasions____<br>Don't know	
SU16	In the past 30 days, did you consume any home brewed alcohol, any alcohol brought over the border/from another country, any alcohol not intended for drinking or other untaxed alcohol?	Yes<br>No→S18	If S16=No then S18
SU17	How many occasions did you consume any home brewed alcohol, any alcohol brought over the border/from another country, any alcohol not intended for drinking or other untaxed alcohol?	Number of occasions___<br>Don't know	
SU18	Do you currently chew betel nuts?	Yes<br>No	If S18=No then S20
SU19	In the last 24 hours, many pieces did you chew?	___pieces	
SU20	Have your ever used heroin, Yama/ice, Ecstasy, Glue, Marijuana, Cocaine, Histamine, Pantazogon, Valium/diazepam, [locally popular drug]?	Yes<br>No<br>Refuse to answer	If No or refuse to answer then D1

Zhang M, *et al. BMJ Open* 2024; 14:e081079. doi: 10.1136/bmjopen-2023-081079

SU21	In the past 12 months have you used any of the drugs?	Yes<br>No<br>Refuse to answer	
S22	Have you ever injected drugs?	No<br>Yes, in past 12 months<br>Yes, before past 12 months<br>Refuse to answer	
	**Disease and symptom**		
D1	Have you ever been diagnosed with chronic lung disease (emphysema, bronchitis, COPD) by a doctor or other health worker?	Yes<br>No→D3	If No, then D3
D2	In the last 30 days, have you been taking any medication or other treatment (like oxygen) for it prescribed by a doctor or other health worker?	Yes<br>No	
D3	In the last 12 months, have you had a tuberculosis (TB) test? I mean, has a doctor examined your sputum (taken a sample of the substance spit out from a deep cough and sent it to a laboratory for analysis) or made an x-ray of your chest?	Yes<br>No→D5	If no, then D5
D4	Have you been taking any medication or treatment during the last 30 days prescribed by a doctor or other health worker?	Yes<br>No	
D5	Have you had fever in the past 30 days?	Yes<br>No→D7	If No, then D7
D6	How long have you had fever?	One week or more<br>No or less than one week	
D7	Are you currently coughing?<br><br>Prompt:<br>If participants answer induced coughing due to environmental factor or substance use, prompt: I mean	Yes<br>No→10	If No, then D10

Zhang M, *et al. BMJ Open* 2024; 14:e081079. doi: 10.1136/bmjopen-2023-081079

	coughing throughout the day, even if you are not smoking or in the environment.		
D8	What is the duration of coughing continuously?	Two weeks or more<br>Less than two weeks	
D9	Are you coughing up blood?	Yes<br>No	
D10	Have you lost body weight unintentional/ suddenly?	Yes<br>No	
D11	In the past 30 days, have you experienced drenching night sweats?	Yes<br>No	If D5-D11 checked 2 or more symptoms, or coughing up blood, then refer to TB care
D12	Have you been diagnosed with Asthma (an allergy respiratory disease) by a doctor or other health worker?	Yes<br>No→D14	If No, then D14
D13	In the last 30 days, have you been taking any medication or treatment prescribed by a doctor or other health worker?	Yes<br>No	
D14	Have you ever had your blood pressure measured by a doctor or other health worker?	Yes<br>No->D17	If No, then D17
D15	Have you ever been told by a doctor or other health worker that you have raised blood pressure or hypertension?	Yes<br>No→D17	If No, then D17
D16	In the past 30 days, have you taken any drugs (medication) for raised blood pressure prescribed by a doctor or other health worker?	Yes<br>No	

Zhang M, *et al. BMJ Open* 2024; 14:e081079. doi: 10.1136/bmjopen-2023-081079

D17	Have you ever had your blood sugar measured by a doctor or other health worker?	Yes<br>No→D20	If No, then D20
D18	Have you ever been told by a doctor or other health worker that you have raised blood sugar or diabetes?	Yes<br>No→D20	If No, then D20
D19	In the past 30 days, have your taken any drugs (medication) or insulin injection for diabetes prescribed by a doctor or other health workers?	Yes<br>No	
D20	Have you ever had your cholesterol (fat levels in your blood) measured by a doctor or other health worker?	Yes<br>No→D23	If No, then D23
D21	Have you ever been told by a doctor or other health worker that you have raised cholesterol?	Yes<br>No→D23	If No, then D23
D22	In the past 30 days, have you taken any oral treatment (medication) for raised total cholesterol prescribed by a doctor or other health worker?	Yes<br>No	
D23	Have you ever had a heart attack or chest pain from heart disease (angina) or a stroke (cerebrovascular accident or incident)?	Yes<br>No	
D24	Are you currently taking medications regularly to prevent or treat heart disease?<br>(for example: aspirin, statins- Lovastatin/ Simvastatin/ Atorvastatin/ any other statin)	Yes<br>No	
D25	Besides the health conditions we have just spoken about, do you currently have other chronic diseases that have been diagnosed by a doctor or health care provider?<br><br>(check all that apply)	No other disease<br>Cancer<br>Liver cancer<br>bronchial and lung cancer<br>Colon and rectal cancer	If mention the disease or symptoms above, go back to the relevant question

Zhang M, *et al. BMJ Open* 2024; 14:e081079. doi: 10.1136/bmjopen-2023-081079

	If diseases that are mentioned are not in the list, but you know in which system it belongs, check "other, specify" in that system and fill the name of the disease.<br><br>*If you do not know which system the disease should be categorized, then check "Other unknow system disease, specify:_______" on the bottom of the list, and fill in the name of the disease.*	Breast cancer<br>Cervical cancer<br>Leukaemia/lymphoma<br>Other, specify:<br>Endocrine system and metabolic disorder<br>thyroid/goitre<br>Other, specify:________<br>Sensor disorders<br>Glaucoma<br>Cataract<br>Hearing loss<br>Other, specify<br><br>Cardiovascular / circulatory disease<br>Enlarged heart<br>congestive heart<br>heart fluttering<br>Cardiac arteriosclerosis<br>Severe cerebrovascular disease<br>Other, specify:____<br><br>Haematological disease<br>Thalassemia<br>Chronic respiratory disease<br>Chronic emphysema<br>Other, specify:_____________<br><br>Digestive disorders<br>Gastritis<br>Acid reflux<br>Liver cirrhosis<br>Other, specify:______<br><br>Genito-urinary disease	

		Kidney failure/kidney disabilities<br>enlarged prostate/inflammation<br>kidney stone/ bladder stone<br>Other, specify:___________<br><br><u>Muscular-skeleton disease</u><br>Gout<br>rheumatoid/ rheumatoid arthritis<br>Chronic knee pain / chronic back pain/ chronic neck pain<br>Paralysis<br>Other, specify:___________<br><br><u>Chronic infection or defective by infection disease</u><br>HIV<br>Polio<br>Leprosy<br>Chronic HBV infection<br>Chronic HCV infection<br>Other, specify:___________<br><br><u>Congenital anomalies</u><br>abnormal intelligence (Down syndrome)<br>Other, specify:___________<br><br><u>Oral disorders</u><br>Periodontitis (gingivitis)<br>Other, specify:___________<br><br><u>Immune system disorder</u><br>Allergy<br>Other, specify:___________	

Zhang M, *et al. BMJ Open* 2024; 14:e081079. doi: 10.1136/bmjopen-2023-081079

		Neurological disorder<br>Parkinson's disease<br>Epilepsy/epilepsy<br>Dementia<br>Other, specify:___________<br><br>Mental health disorder<br>Alcoholism<br>Depression<br>Other, specify:__________<br><br><br>Other unknow system disease, specify:________<br><br>Unknown______	
CC0	You have told us the symptoms and diseases affect your health currently.<br>Now I'd like to ask you about the concerns you have related with health. It can be from the experience of yourself, your family, or your community.<br>I'm going to ask your concerns from different perspective of health, and in the end, I will ask you to rank the ones that concern you the most.		
CC1	The first 3 questions about different dimensions of health services.<br><br>I'm going to ask first if you have concerns about accessing health services, which means the service is provided somewhere, but you cannot receive it<br>Do you have any concerns about health service accessibility?	- Affordability- Cost of care<br>- Geographic accessibility- Travel to service provider (too far, take too much time, no good transportation methods, too sick to travel…)<br>- Language barrier<br>- Culture barrier<br>- Other, specify____________<br>- No concern	

Zhang M, et al. BMJ Open 2024; 14:e081079. doi: 10.1136/bmjopen-2023-081079

	Prompt:<br>If the participant said no concern, then prompt service accessibility can be the cost, going to the place, or problem with communication, or something related with culture that create problem for people to get health care.<br><br>*Please record all that the participant mentioned.*		
CC2	The next question is about concerns of health service availability, which means the service is not provided, or some elements required for providing the service is absent or not enough<br>Do you have any concerns related with heath service availability?<br><br>Prompt:<br>If the participant expressed there is no concern, prompt- is there some health care service they want to use but cannot find the service? Or the service is not fully available because of health facility, health care professionals, equipment- laboratory, bed, ultrasound, or medication are absent or not enough.<br><br>*Please record all that the participant mentioned.*	- Physical presence of service (the service is not provided, or not provided all the time or at all. E.g. no dental care in the primary care unit)<br>- Health facility (health centre or hospital)<br>- Health care professionals (doctors/nurses/paramedical professionals)<br>- Beds or equipment<br>- Medication<br>- Other, specify:______<br>- No concern	
CC3	The next question is about your concerns on quality of health service, which means the service is available, and you are able to receive the service, but you are not satisfied with the service<br>Do you have concerns in the quality of health service?<br><br>Prompt:	- Safety: quality care should avoid harms to people whom the care is intended<br>- Timely: waiting time and delays<br>- Efficient: maximize the be benefit of available resources<br>- Other, specify______<br>- No concern	

Zhang M, *et al. BMJ Open* 2024; 14:e081079. doi: 10.1136/bmjopen-2023-081079

	If the participant expressed no concern, prompt it can be related with safety, timely, or efficiency of the service provided.<br><br>*Please record all that the participant mentioned.*		
CC4	We have spoken about concerns related with health services, now Is like you to think about the environment around your household or at work that can potentially harm yours or others' health.<br><br>Do you have concerns related with your house, around your house, or things/ animals belong to your household, that could harm your health or other's health?<br><br>Prompt:<br>If the participant expressed no concern, prompt- it can be part of the structure of the house like wall, floor material; or something inside of the house related with health like toilet, drinking water source; or animals the household own; in the yard, garden of the household.<br><br>*Please record all that the participant mentioned.*	- House (e.g. wall, roof, floor)<br>- Amenities (e.g. toilet, cooking stove, mosquito net, drinking water)<br>- Animals (e.g. free range pigs in the yard)<br>- Other factors in and around the house, specify______<br>- No concern	
CC5	Do you think there are some risks to your or others' health related with work?<br><br>Prompt:<br>If the participant expressed no concern, prompt: for example, you often risk to get injured when you work, certain things/ products used at your work can harm your health.<br>If you work in the forest or in the rice farm, there are some insects or wild animal can harm you too.	- Risk to physical injuries<br>- Risk of chemical exposures<br>- Risk of exposure to nature environment (e.g. mosquito, snake bite)<br>- Other work-related risk factors, specify________<br>- No concern	

Zhang M, *et al. BMJ Open* 2024; 14:e081079. doi: 10.1136/bmjopen-2023-081079

	Please record all that the participant mentioned.		
CC6	Do you have other concerns about health of yours or people around you?<br>*Please record all that the participant mentioned.*	Presence of disease or symptoms (respondent's or other people)<br>Other, specify________<br>No other concerns	
CC7	Thank you very much for sharing your concerns. You have told me your concerns are: [show results of CC1-CC6]<br>Now, could you (pick the biggest 3 concerns and) rank them? Start from the biggest concern.<br><br>*If the participant expressed less than 3 concerns, then rank the concern(s) mentioned, and select "NA" in the unused field.*	**Top1:________**<br>**Top2:________**<br>**Top3:________**	Automatic showing a list of options selected for CC1-CC6, and pick from the options.

Zhang M, *et al. BMJ Open* 2024; 14:e081079. doi: 10.1136/bmjopen-2023-081079

	Adult women section		
Code	Question	Answers	Skip logic
W1	Now I would like to talk about family planning - the various ways or methods that a couple can use to delay or avoid a pregnancy. Have you ever heard of (METHOD)? (Check all that applied)	Condom<br>Pill<br>IUD<br>Injectables<br>Implants<br>Female condom<br>Emergency contraception<br>Standard days method<br>Lactational amenorrhea method<br>Rhythm method<br>Withdrawal<br>Female sterilization<br>Male sterilization<br>Other modern method, specify: ______<br>Other traditional method, Specify: ___________<br>None<br>Refuse to answer	
W2	Are you currently pregnant?	Yes→W5<br>No or unsure	If yes, W5
W3	Are you or your partner currently doing something or using any method to delay or avoid getting pregnant?<br><br>Prompt: are you or your partner doing any of the following to avoid pregnancy: deliberately avoiding sex on certain days, using a condom, using withdrawal or using emergency contraception?	Yes<br>No→W5<br>Refuse to answer→W5	If No or Refuse to answer, then W5
W4	What are you currently using?	None	

Zhang M, *et al. BMJ Open* 2024; 14:e081079. doi: 10.1136/bmjopen-2023-081079

	**<u>Do not read the answer, check all that applied.</u>**	Female sterilization<br>Male sterilization<br>IUD<br>Injectables<br>Implants<br>Pill<br>Condom<br>Female condom<br>Emergency contraception<br>Standard days method<br>Lactational amenorrhea method<br>Rhythm method<br>Withdrawal<br>Other modern method, specify:____<br>Other traditional method, specify:____	
W5	Now I would like to ask about all the birth you have had during your life. Have you ever given birth?<br>(if No prompt: including the times that a child was born alive but later died, or born dead)	Yes<br>No→ End of the section	If No, then end of the section.
W6	Now I would like to ask some questions about your last birth		
W7	How long ago it was?	_______years →W9<br>Don't know→W8	
W8	How old is the child now?	Age _____years<br>Don't know	If W7=don't know
W9	Did you see anyone for antenatal care for this pregnancy?	Yes<br>No→W12	If No, then W12
W10	Where did you receive antenatal care for this pregnancy?<br><br>Anywhere else?	<u>Home</u><br>Her home→W11<br>Other home→W11<br><br><u>Public sector →W12</u>	If W10= Home/ others then W11

	Check all that apply<br><br>[Insert locally relevant list of health facilities]	Government hospital<br>Government health centre<br>Government health post<br>Other public sector<br><br>Private medical sector –>W12<br>Private hospitalPrivate clinic<br>Other private medical facility<br><br>NGO medical sector→ W12<br>NGO hospital<br>NGO clinic<br>Other NGO medical<br><br>Other, specify: ______-->W11	
W11	Whom did you see when you received the antenatal care at [her home/ other home]? Anyone else?<br><br>*Check all that apply*<br>Prompt: probe to have information on the health care worker's professional background. Distinguish among different health care professionals.<br><br>[insert locally relevant list]	Health personnel<br>Doctor<br>Nurse/midwife<br>Auxiliary midwife<br><br>Other person<br>Traditional birth attendant<br>Community health worker/<br>Field worker<br><br>Other, specify: ___________	
W12	Where did you give birth to this child?	Home<br>Her home →W13<br>Other home→W13<br><br>Public sector →End of the section<br>Government hospital	

Zhang M, *et al. BMJ Open* 2024; 14:e081079. doi: 10.1136/bmjopen-2023-081079

		Government health centre<br>Government health post<br>Other public sector<br><br>Private medical sector →End of the section<br>Private hospital<br>Private clinic<br>Other private medical sector<br><br>NGO medical sector<br>NGO hospital<br>NGO clinic<br>Other NGO medical<br>Other, specify: ______ -->W13	
W13	Who assisted with the delivery?<br><br>Prompt: probe to have information on the health care worker's professional background. Distinguish among different health care professionals.<br><br>Check all that apply	Health personnel<br>Doctor<br>Nurse/midwife<br>Auxiliary midwife<br><br>Other person<br>Traditional birth attendant<br>Community health worker/<br>Field worker<br><br>Other, specify: ________	If W12= Home

	Children's section		

Code	Question	Answers	Skip logic
C1	We would like to ask you question about the [NAME]' health, including breastfeeding, vaccination history.<br>Are you willing to answer the questions?<br>Do you think you are able to answer the questions?	No, I don't want to/ or cannot answer the questions → C2<br>Yes, I'm willing to and able to answer the questions→C3	If No, then C2
C2	Could you tell us who is the best person to answer the questions about [Name] health?	Identify the member list, and ID	Restart from C1 with the person pointed
C3	Relationship of the person answering the question to the child	Mother<br>Father<br>Adopted/foster/ stepmother<br>adopted/foster/ stepfather<br>Grandmother<br>Grandfather<br>Sister<br>Brother<br>Other, specify:________	
C4	Has [NAME] ever been breastfed?	Yes<br>No→C7	If No, then C7
C5	Is [NAME] still breastfed?	Yes<br>No→7	If No, then C7
C6	Did (NAME) drink or eat anything other than breast milk yesterday or last night? Including plain water, other milk, non-milk liquid, or complementary food)	Yes<br>No	
	**Vaccination history**		**Only ask if 0-35 months child→ C7-C17, else C18**
C7	Now I would like to ask some questions about vaccinations received by [NAME].<br><br>Do you have a card or other document where (NAME)'s vaccinations are written down?	Yes, has only a card<br>Yes, has only another document<br>Yes, has card and other document<br>No, no card and no other document→C15	

C8	May I see the card or other document where (NAME)'s vaccinations are written down?	Yes, only card seen<br>Yes, only other document seen<br>Yes, card and other document seen<br>No card and no other document seen→C15	If NO then C16
C9	*Don't need to ask the question, compare the record in the vaccination card or document with vaccination schedule of Thailand. Check appropriate vaccines according to the child's age, and choose the option.*<br><br>*(show card)*<br>*Use the vaccination schedule in the show card to compare with the child's vaccination card*	Completed all age-appropriate vaccines→C12<br>Missing 1 dose age-appropriate vaccine<br>Missing more doses age-appropriate vaccines	If completed all age-appropriate vaccines, then C12
C10	Were age-appropriate doses of hepatitis B vaccination given according to the vaccination card or other document?	Yes, completed all age-appropriate doses – C12<br>Received the birth dose, but some doses missing according to children's age →C12<br>There is no record showing hepatitis B vaccination was given	
C11	At or soon after birth, did (NAME) receive a Hepatitis B vaccination, that is, an injection in the thigh to prevent Hepatitis B?	Yes<br>No<br>Don't know	If C11= There is no record
C12	Was a dose of vitamin A supplement given in the past 6 month according to the vaccination card or other document?	Yes →C14<br>No	
C13	In the past 6 months, did [Name] receive a vitamin A dose. (Show common type of ampules/capsules/syrups)	Yes<br>No<br>Don't know	If C13=No
C14	Ask the respondent for permission to photograph vaccination card or other document where vaccinations are written. If permission is granted, photograph card.	Photograph taken<br>Photograph not taken, permission not received<br>Photograph not taken, other reason<br><br>➔ End of the section	

C15	Did (NAME) ever receive any vaccinations to prevent (NAME) from getting diseases, including vaccinations received in campaigns or immunization days or child health days?	Yes<br>No → C18<br>Don’t know	If No/ don’t know, then C18
C16	How many times did [Name] receive vaccination?	____times<br>Reported completed all age-appropriate vaccine<br>Don’t know	
C17	At or soon after birth, did (NAME) receive a Hepatitis B vaccination, that is, an injection in the thigh to prevent Hepatitis B?	Yes<br>No<br>Don’t know	
C18	In the past 6 months, did [Name] received vitamin A dose.<br>Show common type of ampules/capsules/syrups)	Yes<br>No<br>Don’t know	All under 5 years

The end of questionnaire

Zhang M, *et al. BMJ Open* 2024; 14:e081079. doi: 10.1136/bmjopen-2023-081079
