# Supplementary material for: Defining the hidden burden of disease in rural communities in Bangladesh, Cambodia and Thailand: a cross-sectional household health survey protocol
Source: BMJ Open. 2024 Mar 23;14(3):e081079. doi: 10.1136/bmjopen-2023-081079 (PMC10961499; doi:10.1136/bmjopen-2023-081079)
Supplement: Author's manuscript [file bmjopen-2023-081079.draft_revisions.pdf]

BMJ Open is committed to open peer review. As part of this commitment we make the peer review history of every article we publish publicly available.

When an article is published we post the peer reviewers' comments and the authors' responses online. We also post the versions of the paper that were used during peer review. These are the versions that the peer review comments apply to.

The versions of the paper that follow are the versions that were submitted during the peer review process. They are not the versions of record or the final published versions. They should not be cited or distributed as the published version of this manuscript.

BMJ Open is an open access journal and the full, final, typeset and author-corrected version of record of the manuscript is available on our site with no access controls, subscription charges or pay-per-view fees (http://bmjopen.bmj.com).

If you have any questions on BMJ Open's open peer review process please email info.bmjopen@bmj.com

BMJ Open

DEFINING THE HIDDEN BURDEN OF DISEASE IN RURAL COMMUNITIES IN BANGLADESH, CAMBODIA AND THAILAND: A CROSS-SECTIONAL HOUSEHOLD HEALTH SURVEY PROTOCOL

Journal:	*BMJ Open*
Manuscript ID	bmjopen-2023-081079
Article Type:	Protocol
Date Submitted by the Author:	18-Oct-2023

Complete List of Authors:	Zhang, Meiwen; Mahidol Oxford Tropical Medicine Research Unit; University of Oxford, Centre for Tropical Medicine and Global Health, Nuffield Department of Medicine<br>Htun, Nan Shwe Nwe; Mahidol Oxford Tropical Medicine Research Unit<br>Islam, Shayla; BRAC, Communicable Diseases Program<br>Sen, Aninda; BRAC, Communicable Diseases Program<br>Islam, Akramul; BRAC, Communicable Diseases Program<br>Neogi, Amit Kumer; BRAC, Communicable Diseases Program<br>Tripura, Rupam; Mahidol University, Mahidol-Oxford Tropical Medicine Research Unit, Faculty of Tropical Medicine<br>Dysoley, Lek; Cambodian National Malaria Control Program<br>Perrone, Carlo; Mahidol Oxford Tropical Medicine Research Unit<br>Chew, Rusheng; Mahidol Oxford Tropical Medicine Research Unit; University of Oxford, Centre for Tropical Medicine and Global Health, Nuffield Department of Medicine<br>Batty, Elizabeth M.; Mahidol Oxford Tropical Medicine Research Unit; University of Oxford, Nuffield Department of Medicine<br>Thongpiam, Watcharintorn; Mahidol Oxford Tropical Medicine Research Unit<br>Wongsantichon, Jantana; Mahidol Oxford Tropical Medicine Research Unit<br>Menggred, Chonticha; Mahidol Oxford Tropical Medicine Research Unit<br>Zaman, Sazid Ibna; Mahidol Oxford Tropical Medicine Research Unit; University of Oxford, Centre for Tropical Medicine and Global Health, Nuffield Department of Medicine<br>Waithira, Naomi; Mahidol Oxford Tropical Medicine Research Unit; University of Oxford, Centre for Tropical Medicine and Global Health, Nuffield Department of Medicine<br>Blacksell , Stuart; Mahidol Oxford Tropical Medicine Research Unit; University of Oxford, Centre for Tropical Medicine and Global Health, Nuffield Department of Medicine<br>Liverani, Marco; London School of Hygiene & Tropical Medicine, Department of Global Health and Development; Mahidol University, Faculty of Public Health<br>Lee, Sue; Mahidol Oxford Tropical Medicine Research Unit; University of Oxford, Centre for Tropical Medicine and Global Health, Nuffield Department of Medicine<br>Maude, Richard; Mahidol Oxford Tropical Medicine Research Unit; University of Oxford, Centre for Tropical Medicine and Global Health,

Nuffield Department of Medicine
Day, Nicholas; Mahidol Oxford Tropical Medicine Research Unit; University of Oxford, Centre for Tropical Medicine and Global Health, Nuffield Department of Medicine
Lubell, Yoel ; Mahidol Oxford Tropical Medicine Research Unit; University of Oxford, Centre for Tropical Medicine and Global Health, Nuffield Department of Medicine
Peto, Thomas; Mahidol Oxford Tropical Medicine Research Unit; University of Oxford, Centre for Tropical Medicine and Global Health, Nuffield Department of Medicine

Keywords: INFECTIOUS DISEASES, Risk Factors, Chronic Disease, Quality of Life, EPIDEMIOLOGY

SCHOLARONE™
Manuscripts

DEFINING THE HIDDEN BURDEN OF DISEASE IN RURAL COMMUNITIES IN BANGLADESH, CAMBODIA AND THAILAND: A CROSS-SECTIONAL HOUSEHOLD HEALTH SURVEY PROTOCOL

Meiwen Zhang[1,2], Nan Shwe Nwe Htun[1], Shayla Islam[3], Aninda Sen[3], Md Akramul Islam[3], Amit Kumer Neogi[3], Rupam Tripura[1], Lek Dysoley[4], Carlo Perrone[1], Rusheng Chew[1,2,8], Elizabeth M. Batty[1,2], Watcharintorn Thongpiam[1], Jantana Wongsantichon[1], Chonticha Menggred[1], Sazid Ibna Zaman[1,2], Naomi Waithira[1,2], Stuart D. Blacksell[1,2], Marco Liverani[5-7], Sue J Lee[1,2,9], Richard J. Maude[1,2,10], Nicholas P.J. Day[1,2], Yoel Lubell[1,2], Thomas J. Peto[1,2]

1. Mahidol Oxford Tropical Medicine Research Unit, Bangkok, Thailand
2. Centre for Tropical Medicine and Global Health, Nuffield Department of Medicine, University of Oxford, Oxford, UK
3. Communicable Diseases Program, BRAC, Dhaka, Bangladesh
4. Cambodian National Malaria Control Program, Phnom Penh, Cambodia
5. Department of Global Health and Development, London School of Hygiene & Tropical Medicine, London, UK
6. Faculty of Public Health, Mahidol University, Bangkok, Thailand
7. School of Tropical Medicine and Global Health, Nagasaki University, Nagasaki, Japan
8. Faculty of Medicine, University of Queensland, Brisbane, Australia
9. Monash University and Alfred Hospital, Department of Infectious Diseases, Melbourne, Australia
10. The Open University, Milton Keynes, UK

For peer review only

Abstract

Introduction

In low- and middle-income countries in Southeast Asia, the majority of the population resides in rural areas where the burden of diseases remains poorly understood, posing a challenge for effective healthcare prioritization and resource allocation. Addressing this knowledge gap, the South and Southeast Asia Community-based Trials Network (SEACTN) will undertake a prevalence survey, as one of the key initiatives of its first project- the Rural Febrile Illness project (RFI). This survey, alongside other RFI studies that explore fever etiology, leading causes of mortality, and establishing village and health facility maps and profiles, aims to provide an updated epidemiological background of the rural areas where the network is operational.

Methods and analysis

During 2022-2023, a cross-sectional household survey will be conducted across three SEACTN sites in Bangladesh, Cambodia, and Thailand. Using a two-stage cluster-sampling approach, we will employ a probability-proportional-to-size sample method for village, and a simple random sample for household, selection, enrolling all members from the selected households. Approximately 1500 participants will be enrolled per country. Participants will undergo questionnaire interview, physical examination and hemoglobin point-of-care testing. Blood samples will be collected and sent to central laboratories for

analysis, to test for chronic and acute infectious diseases, and biomarkers associated with cardiovascular disease and diabetes. Prevalences will be presented as an overall estimate by country, and stratified and compared across sites and participants' sociodemographic characteristics. Associations between disease status, risk factors, and other characteristics will be explored.

Ethics and dissemination

This study protocol has been approved by the Oxford Tropical Research Ethics Committee, National Research Ethics Committee of Bangladesh Medical Research Council, the Cambodian National Ethics Committee for Health Research, the Chiang Rai Provincial Public Health Research Ethical Committee. The results will be disseminated via the local health authorities and partners, peer-reviewed journals, and conference presentations.

297/300

Trial registration

Clinicaltrials.gov, NCT05389540.

Strengths and limitations

- The study will utilize diverse methods (interviews, physical examinations, and laboratory tests) to gather extensive data on the prevalence, risk factors, healthcare utilization patterns of both communicable and non-communicable diseases
- The use of two-stage cluster sampling enables the sample to be representative of each study area.
- As an integrated component of the Rural Febrile Illness project, the findings will be synergized with outcomes of concurrent studies in these specified regions, offering a multidimensional overview of health and healthcare provision.
- The findings are specific to the selected rural areas, and should be interpreted with caution when considering broader implications for rural populations in the study countries.

Introduction

Life expectancy in Southeast Asia increased from 63 to 71 years between 2000 and 2019 (1). These positive changes can be attributed, in part, to advancements in the region's health systems, which have addressed traditional high-burden diseases, such as infectious diseases, maternal and neonatal health, and under 5 mortality (2). Despite these improvements, the burden of traditionally significant diseases persists, particularly in rural areas. Furthermore, there is a transition in disease epidemiology due to rapidly changing environments, growing economies, and ageing populations (3). The shift is marked by a rising burden of non-communicable diseases and injuries, along with emerging infectious diseases (e.g. COVID-19 and chikungunya) (4,5).

The knowledge of current disease epidemiology in the region, however, is limited, constraining health systems' ability to identify healthcare priorities and direct future resource allocation (6). While the Global Burden of Disease study provides insights into disease burdens across countries and diseases, the accuracy of its results is impeded by the scarcity of recent epidemiological data (6). For instance, less than 10% of

For peer review only

data sources from Cambodia, Myanmar and Laos were collected since 2010, from a pool of about four to five hundred publications and datasets from each of these countries(7). Given the limited coverage of disease reporting and surveillance systems in the region, research data is pivotal in bridging this information gap. Such data not only offers up-to-date information for modeling studies, but is also critical for optimizing resource allocation by including factors such as disease severity, the magnitude of the population affected, and equity (8).

Moreover, the marked systemic disparities in healthcare between rural and urban areas in the region highlight the need for rural-specific epidemiological data(3). Despite 50%- 85% of the region's population residing in rural areas, this large population group remains relatively understudied compared to countrywide estimates (7,9–13). In this context, cross-sectional surveys are an effective research method to provide timely estimates of disease prevalence in rural communities, through cost-effective approaches combining questionnaire interviews, clinical examinations, and laboratory tests.

The newly-formed South and Southeast Asia Community-based Trials Network (SEACTN) aims to establish a network of community-based healthcare providers and facilities capable of implementing interventions designed to triage, diagnose, and treat patients within rural communities across five South and Southeast Asian countries (Bangladesh, Cambodia, Laos, Myanmar, and Thailand) (14). The first project is the Rural Febrile Illness Project (RFI), dedicated to delineating the epidemiological baseline of febrile illness in remote and underserved regions, where febrile illness and access to healthcare pose significant health challenges (14). RFI encompasses diverse initiatives designed to gain a multifaceted understanding of the health dynamics within these communities to effectively facilitate the identification of interventions for future studies across SEACTN. Recognizing the gaps in understanding of disease prevalence, a cross-sectional household health survey has been planned as one of the key initiatives of the RFI.

This survey aims to define the prevalence of a broad spectrum of communicable and non-communicable causes of health conditions in areas where the network operates. Alongside the survey, other key initiatives of RFI include a fever etiology study to determine the incidence, causes, and outcomes of febrile illness; a verbal autopsy study to identify common causes of mortality and the circumstances surrounding death; and a village and health facility mapping study creating a detailed profile of the study villages, estimating travel time to health facilities, and identifying health service provision gaps. Each initiative holds significant value as an independent study, and their results will be complementary providing a thorough comprehension of healthcare needs and enabling more equitable resource allocation.

Methods and analysis

The SEACTN household health survey is a community-based cross-sectional survey aiming to provide an overview of the burden of disease in selected rural areas where the network is operational. The first participant was enrolled on October 3, 2022, and data collection is expected to continue until December, 2023.

Primary objectives and outcomes

1. To determine the prevalences of selected diseases and exposure to locally prevalent or important pathogens, including, but not limited to:

For peer review only

a. Immunoglobulin G (IgG) against selected common pathogens causing fever.
b. Hepatitis B and C
    i. Hepatitis B virus (HBV) surface antigen
    ii. IgG against Hepatitis C virus (HCV) and presence of HCV
c. Selected common non-communicable diseases (e.g. diabetes, hypertension, stroke) according to self-reported disease history and laboratory tests.
d. Self-reported illness or injury in the past 30 days.
    i. Any acute conditions (e.g. fever, persistent cough, watery diarrhea)
    ii. Injury or death caused by an accident

2. To determine the point prevalence of different self-perceived health statuses.
3. To determine the prevalences of major risk factors for common non-communicable diseases (e.g. smoking, alcohol consumption, overweight).

Study population and setting

All SEACTN operational areas were selected by partner organizations as they represent poor, remote, and rural communities with limited access to formal healthcare (14). This study will be conducted in three areas, consisting of 391 villages in Bangladesh, Cambodia and Thailand (Figure 1). The other sites will not take part of the study due to operational constraints.

Two-stage cluster sampling will be used to obtain a representative sample from each study site. In the first stage, 75 villages will be selected using the probability-proportional-to-size sample method (15–17). For the second stage, within each village, a simple random sample of five to seven households (adjusted to the average household size) will be selected using computer-generated random sampling from the village household list.

Inclusion and exclusion criteria

For each selected household, all usual household members or visitors who stayed overnight in the households before the survey are eligible to be enrolled (18).

Sample size

This study is adequately powered to determine the prevalences of all key indicators within each age group, with a design effect of 1.5 and a range of 5-10% degrees of precision, while considering a maximum of 1500 participants per study country (Table 1). To account for a 10% non-response rate, assuming an average household size of 4.5 persons, and enrolling all household members, the final sample size was adjusted to 1667 participants per country (19–21).

The minimal sample size required was calculated based on the estimated prevalences of key indicators overall and within each population age group (Table 1). Prevalence estimates, population proportions within each age group, and average household size, were derived from previously conducted national health surveys and published studies, preferably from the study country, or from countries with a similar epidemiological context (19–29).

For peer review only - http://bmjopen.bmj.com/site/about/guidelines.xhtml

Study procedures

Standard operating procedures (SOPs) were developed for field procedures, sample collection and transportation. Training materials were developed and training sessions were delivered to all the study staff directly, or using a train-the-trainer model where field supervisors and managers will be trained first and then subsequently train other field staff. All research staff are experienced in community-based research or interventions. Experienced phlebotomists were recruited and trained in specimen collection and processing in the field. A monitoring and evaluation plan was devised to maximize the research quality.

Patient and Public Involvement Statement

The potential challenges for the study include comprehensibility of the questionnaires, and willingness of selected households to participate. We have proactively addressed these concerns by involving the target population in the questionnaire validation process (section: Questionnaire interviews), and planning community mobilization events before the survey, tailored to the local context.

Collaborators will identify contact points from local stakeholders and communities, such as village leaders, local health center staff, or community health workers, and determine the most suitable methods for establishing contact. Main events may involve community engagement meetings to explain the study purpose, organization and procedures, and the plan for dissemination of study results. Prior to the survey day for each village, the study team will meet, or call if physical meetings are not feasible, the household head or members of the selected households. With the support of the key contact persons, this interaction will create an opportunity to address questions or concerns, enabling the survey teams to work with each community and selected household to plan an effective data collection schedule.

Survey schedule

On the scheduled survey day for each village, stations will be set up at a convenient location within or near the village. Household members will arrive at the survey location following their appointment to complete the procedures (Figure 2).

Informed Consent

Participants must sign the informed consent form before any study-specific procedures are performed. The informed consent will be available in the official local language. It will be presented to prospective participants by trained study staff detailing the study procedures and implications of study participation. It will be made explicit that participation is voluntary and participants are free to withdraw from the study at any time. If necessary, the informed consent process will be interpreted to dialect when participants do not speak the official local language.

Adequate time will be given to study participants to consider the information and ask questions. Written consent will then be obtained from the participants, or the caretakers of participants, in compliance with local legal age requirements, using the participant's dated signature or thumbprint (if unable to write) and dated signature of a person who presented and obtained the informed consent. A copy of the signed Informed Consent document will be given to the participant. Children aged as dictated by local legislation and regulations will be required to sign the written informed assent form in addition to their parent or guardian signing a consent form.

For peer review only

Questionnaire interviews

The questionnaire consists mostly of questions adapted from well-validated and widely implemented tools (Appendix A). This approach enables direct comparison of results with previously conducted surveys by incorporating questions from the Demographic and Health Surveys (DHS), World Health Organization (WHO) STEPWise surveys for Non-communicable Disease Risk Factors (STEP), and the World Health Survey (30–32).

The questionnaire underwent adjustments and validation through pretests using behavior coding and participant debriefing (33,34). During mock interviews, the observer of each interview pair recorded the interaction problems for each question, such as “major change in wording”, “question reading interrupted by the respondent”, or “additional prompt is provided”. All interviewers, interviewees, and the observers participated in the debriefing. The pretest was conducted initially among peer researchers in English. Subsequently, the adjusted version was translated into Thai and tested among the target population in Thailand. This ensured the questionnaire’s suitability, comprehensibility, and appropriate length for administration. The final questionnaire was then translated into the official languages of each study country (Bangla, Khmer, and Thai) by professional translators or dedicated staff from partner organizations, and validated by the local research teams. When necessary, during face-to-face interviews, questions will be interpreted to dialect for participants who do not speak the official local language.

The questionnaire consists of four sections answered by household members based on their sex and age (Figure 2, top).

- The household section is completed by the household head, providing information on household characteristics, and a list of all household members along with any illnesses in the past 4 weeks and health seeking behavior.
- The adult section is for household members aged 15 years or older and includes questions on socio-demographic characteristics (e.g., age, sex, education, marital status, and occupation), disease history (e.g. tuberculosis, diabetes, cardiovascular disease), self-perceived health status, health concerns, substance use (e.g., tobacco and alcohol), and other disease risk factors.
- The women’s health section is for female household members aged 15 years or older, and includes questions on contraception use, utilization of antenatal care and delivery services.
- The children’s health section is answered by caretakers of children under 5 years, providing information on each child’s vaccination and breastfeeding history.

Physical examination

All household members will undergo weight, height, and tympanic temperature measurements. Children under 5 years will also have their mid-upper arm circumference (MUAC) measured, and adult household members ($\geq$15 years) will undergo blood pressure measurement (Figure 2, middle). Weight will be measured with a digital scale, preferably with a taring function allowing a child's weight to be measured while being held by an adult. Height measurements will be carried out with measuring scales/boards. Children younger than 24 months will be measured lying down on the board, while standing height will be measured for older children. Tympanic temperature will be measured with infrared thermometers.

All measures will be taken by experienced and trained staff. Consistency and comparability will be ensured by using the same validated devices previously used in studies in similar settings, whenever possible, across study sites.

For peer review only

Blood sample collection and point-of-care hemoglobin test

Blood samples will be collected from participants aged six months or older (Figure 2, bottom). For participants aged 6 months to under five years, four dried blood spots (DBS) will be collected via finger or heel prick. Participants aged five years or older will provide venous blood samples, with four milliliters collected from those aged five to under 15 years, and six milliliters from participants aged 15 years or older (Figure 2, bottom). After blood sample collection, appropriate samples will be tested for hemoglobin using HemoCue® Hb 301 system (HemoCue®AB, Ängelholm, Sweden) to detect anemia.

The venous blood samples will be temporarily stored in cool boxes between 4°C and 8°C, for a maximum of 24 hours before centrifugation in the field laboratories. Aliquots of whole blood, serum, plasma, and packed red blood cells of these samples will be stored at a minimum of -20°C or below without freeze-thaw until analysis. DBS will be air-dried in the laboratory for at least six hours, after which they will be placed in a plastic zip-lock bag and stored at room temperature or at 4°C or below.

Information, consultation and referral

Upon completion of the field procedures, individual participants will receive the results of their hemoglobin test and physical examinations. Participants with abnormal results will be referred to a study nurse who will provide recommendations and, if the participant wishes, further referrals in accordance with local guidelines.

Analysis of blood samples

Sample aliquots and DBS will be shipped to the central laboratories in Bangkok, Thailand where they will be analyzed. All laboratory activities will be performed by experienced and trained staff. All laboratory procedures will be performed using validated SOPs. Multiplex serology tests will be developed and validated for measures of IgG and immunoglobulin M (IgM) of common pathogens causing fever; all other methods have already been published in peer-reviewed journals.

Malaria polymerase chain reaction (PCR) will be performed for all participants to identify individuals with malaria parasites. The PCR methods typically have a detection level in the range of 100-1000 parasites per milliliter(35). Plasmodium species will also be identified.

Serology tests for selected common pathogens causing fever including IgG and IgM for Dengue, Chikungunya, Japanese encephalitis, Zika, SARS-CoV-2, *Plasmodium vivax*, *Plasmodium falciparum*, *Orientia tsutsugamushi*, *Rickettsia spp*., and *Leptospira spp*. will be performed for all participants.

Tests for hepatitis B and C: all participants will be tested for HBV surface antigen; participants 15 years or older will also be tested for IgG for HCV, and the positive samples will undergo HCV PCR.

Non-communicable disease-related markers: participants 15 years or older will be tested for Hemoglobin A1c, total cholesterol and high-density lipoprotein cholesterol.

Additionally, the remaining samples will be tested for exposure to, or presence of, pathogens associated with selected diseases that are regionally or locally prevalent or important. The final list of diseases, pathogens, and sample selection will be adjusted based on findings from other SEACTN studies exploring diagnostics and prognostics in febrile patients, and other available evidence.

For peer review only

Results feedback

Individual-level data collected from physical examinations and hemoglobin tests will be provided on the same day once all field procedures are concluded for an individual participant (Section: Study procedure-Information, consultation and referral). Additionally, participants will be thoroughly informed about the implications of the laboratory tests to be performed during the consent process. They will be presented with the option to receive the results of hepatitis B and C tests. If they wish to receive them, the results, once available, will be placed in a sealed envelope and will then be delivered by the local health providers or village leaders to the participants. The decision on which results to provide to individual participants is made in consultation with the site research teams and local clinicians, considering factors such as their clinical relevance, the potential harm that could result from a lack of consultation upon results distribution, and the accessibility of treatment.

Planned analysis

All data obtained through questionnaire interviews, physical examinations, and hemoglobin point-of-care tests will be collected on tablets using an electronic data collection platform - Open Data Kit (36). The electronic case report form has built-in validation rules to identify missing or potentially incorrect data. Stringent checks are applied to variables used to estimate the study outcomes, such as recent fever history, diabetes history, and weight. Survey interviewers cannot progress to the next question if these data are missing or outside the defined ranges. Throughout the survey, data will be continuously updated and monitored for quality. Data queries and quality reports will be generated every two to four weeks for data verification and correction, and to identify areas needing additional training and support, ensuring data integrity.

For descriptive summaries, means and standard deviations (SD) or medians with interquartile range (IQR) for continuous variables, and proportions for categorical variables will be calculated. Outcomes will be presented as an overall estimate by country, and with 95% confidence intervals, stratified and compared across sites and participants' sociodemographic characteristics using appropriate tests, such as the Student's t-test, Mann-Whitney U tests or chi-squared tests. Associations between disease status, risk factors, self-perceived health status, and other characteristics will be explored through univariate and multivariate analyses, and measures of effect will be reported with 95% confidence intervals. Sampling weights will be accounted for, as needed, in the analysis.

For peer review only

Discussion

This prevalence survey conducted across three rural SEACTN study sites in Bangladesh, Cambodia, and Thailand aims to provide a comprehensive epidemiological description of the study areas. Employing a multi-faceted approach, including questionnaire interviews, laboratory tests, and physical examinations, the study will yield extensive data on the prevalence, risk factors, healthcare utilization patterns of both communicable and non-communicable diseases. The findings will contribute to improved estimates of the burden of disease, and deepen the comprehension of findings from other RFI initiatives, offering a thorough overview of health and healthcare provision in these areas. The survey will be invaluable for setting healthcare priorities and directing resource allocation for health system development.

As a core component of RFI, this survey, alongside other initiatives, adopts an integrated approach to address health challenges more effectively, particularly in the context of the "double burden of disease", characterized by the rising burden of non-communicable diseases, alongside ongoing challenges posed by infectious diseases and sub-optimal maternal and child health (2). This survey will encompass various dimensions of health, allowing for further exploration of associations among disease occurrence, risk factors, and self-perceived health. Key data from the survey, such as household wealth status, and community members' perception of their health and health concerns, will contextualize the findings from the other three RFI studies, and provide insights into potential health disparities and inequalities. By integrating disease prevalence with fever etiology and causes of death, a multi-layered understanding of health dynamics in the study area could be attained. Additionally, knowledge from the survey will play a vital role for estimating incidence. This involves assimilating data on proportion of the population with fever who visited various healthcare providers from the survey, with the number of fever patients recruited from the village health workers or health facilities, captured from the fever etiology studies.

The study will also bridge the gap in community-based seroprevalence estimates for common pathogens causing fever, minimizing biases related to health-seeking behavior in facility-based studies. The data can also to be used for population-based incidence estimates (37,38). Furthermore, the updated prevalences of hepatitis B and C can be instrumental in evaluating the coverage and effectiveness of HBV vaccination programs, and inform HCV screening and treatment strategies in the region in the era of direct-acting antiviral agents (39,40). This study also extends its scope to gather insights into the health perspectives and concerns of community members, facilitating the alignment of future interventions with the needs and expectations of the population. Specifically, for self-perceived status, while existing studies primarily focus on the elderly population, this study encompasses a broader age range(41–44). This can inform tailored interventions to improve the quality of life for a more extensive population (45–47).

This study encourages regional collaboration by applying consistent research methods and seeking representative samples across study sites, allowing effective cross-country comparisons and fostering collaborative approaches to address shared health challenges (3).

The findings of this study are limited in their generalizability and should be interpreted with caution when considering broader implications for rural populations in the study countries. However, the study was specifically designed for and conducted within the selected rural areas of Bangladesh, Cambodia, and Thailand, where SEACTN is operational. This focused approach allows for insights into health challenges and opportunities within these areas.

In conclusion, this planned household prevalence survey will significantly contribute to disease prevention and control in the region by offering a comprehensive understanding of health conditions of the rural populations. Complementing the results from other RFI initiatives, it will serve as a foundation for evidence-based interventions, shaping future research and disease control priorities. The study findings will not only serve the next phase of SEACTN but also extend to the broader context, ultimately leading to improved health outcomes in rural communities.

For peer review only

Ethics and dissemination

This study protocol was approved by the Oxford Tropical Research Ethics Committee (OxTREC Ref: 6-22), National Research Ethics Committee of Bangladesh Medical Research Council (BMRC/NREC/2022-2025/69), the Cambodian National Ethics Committee for Health Research (23rd December 2022 NECHR), the Chiang Rai Provincial Public Health Research Ethical Committee (CRPPHO 75/2565).

Safety considerations

Participants may experience slight discomfort during the blood draw, however, our experienced clinical staff performing the phlebotomy will take special attention to minimize this discomfort. While some interview questions may be personal, all data will be handled confidentially, and linked only to study IDs. Participants will also be informed of their right to skip questions or end the interview if they feel uncomfortable.

Data deposition and curation

The database and all electronic data will be stored on secure servers that are backed up daily, with weekly off-site storage. Paper records, if they exist, will be kept in secure storage such as locked cabinets; if necessary, the record will be scanned and stored electronically. Study data will be archived in accordance with Mahidol Oxford Tropical Medicine Research Unit (MORU) SOPs. Electronic data will be de-identified and preserved indefinitely. Paper records will be preserved for five years after study completion. With participant's consent, electronic data can be shared according to the terms defined in the MORU data sharing policy with other researchers for future use (48).

Dissemination

The results of the study will be summarized in plain language in both English and the official local language. These summaries will be disseminated via the local health authorities and partners. The findings will also be disseminated internationally through conference presentations and peer-reviewed academic journal publications, aligning with Wellcome Trust policy and guidelines.

For peer review only

References

1. Life expectancy at birth (years) [Internet]. [cited 2023 Aug 23]. Available from: https://www.who.int/data/gho/data/indicators/indicator-details/GHO/life-expectancy-at-birth-(years)

2. Bygbjerg IC. Double Burden of Noncommunicable and Infectious Diseases in Developing Countries. Science. 2012 Sep 21;337(6101):1499–501.

3. Chongsuvivatwong V, Phua KH, Yap MT, Pocock NS, Hashim JH, Chhem R, et al. Health and health-care systems in southeast Asia: diversity and transitions. The Lancet. 2011 Jan 29;377(9763):429–37.

4. Coker RJ, Hunter BM, Rudge JW, Liverani M, Hanvoravongchai P. Emerging infectious diseases in southeast Asia: regional challenges to control. The Lancet. 2011 Feb 12;377(9765):599–609.

5. Dans A, Ng N, Varghese C, Tai ES, Firestone R, Bonita R. The rise of chronic non-communicable diseases in southeast Asia: time for action. The Lancet. 2011 Feb 19;377(9766):680–9.

6. Murray CJL. The Global Burden of Disease Study at 30 years. Nat Med. 2022 Oct;28(10):2019–26.

7. Global Burden of Disease Study 2019 (GBD 2019) Data Input Sources Tool | GHDx [Internet]. [cited 2023 Aug 23]. Available from: https://ghdx.healthdata.org/gbd-2019/data-input-sources

8. Voigt K, King NB. Out of Alignment? Limitations of the Global Burden of Disease in Assessing the Allocation of Global Health Aid. Public Health Ethics. 2017 Nov;10(3):244–56.

9. World Health Organization. Regional Office for the Western Pacific. UHC and SDG country profiles : Cambodia [Internet]. Manila: WHO Regional Office for the Western Pacific; 2018 [cited 2021 Oct 6]. Available from: https://apps.who.int/iris/handle/10665/272307

10. UHC and SDG country profiles : Lao People's Democratic Republic [Internet]. [cited 2023 Jan 20]. Available from: https://apps.who.int/iris/handle/10665/272312

11. World Health Organization. Regional Office for South-East Asia. 2019 Health SDG profile: Thailand [Internet]. World Health Organization. Regional Office for South-East Asia; 2019 [cited 2021 Oct 6]. Available from: https://apps.who.int/iris/handle/10665/327767

12. World Health Organization. Regional Office for South-East Asia. 2019 Health SDG profile: Bangladesh [Internet]. World Health Organization. Regional Office for South-East Asia; 2019 [cited 2021 Oct 6]. Available from: https://apps.who.int/iris/handle/10665/327747

13. World Health Organization. Regional Office for South-East Asia. 2019 Health SDG profile: Myanmar [Internet]. World Health Organization. Regional Office for South-East Asia; 2019 [cited 2021 Oct 6]. Available from: https://apps.who.int/iris/handle/10665/327762

14. Chandna A, Chew R, Shwe Nwe Htun N, Peto TJ, Zhang M, Liverani M, et al. Defining the burden of febrile illness in rural South and Southeast Asia: an open letter to announce the launch of the Rural Febrile Illness project. Wellcome Open Res. 2022 Mar 10;6:64.

15. Department of Economic and Social Affairs, United Nation. Designing Household Survey Samples: Practical Guidelines [Internet]. United Nation Publication; 2008. Available from: chrome-

For peer review only

extension://efaidnbmnnnibpcajpcglclefindmkaj/https://unstats.un.org/unsd/publication/seriesf/seriesf_98e.pdf

16. The World Health Organization. The WHO STEPwise approach to noncommunicable disease risk factor surveillance [Internet]. 2020 [cited 2023 May 25]. Available from: https://www.who.int/teams/noncommunicable-diseases/surveillance/systems-tools/steps/manuals

17. Milligan P, Njie A, Bennett S. Comparison of two cluster sampling methods for health surveys in developing countries. Int J Epidemiol. 2004 Jun 1;33(3):469–76.

18. ICF. Demographic and Health Survey Interviewer's Manual [Internet]. Rockville, Maryland, USA: ICF; 2020 [cited 2023 Sep 22]. Available from: https://dhsprogram.com/publications/publication-dhsm1-dhs-questionnaires-and-manuals.cfm

19. MoHS/Myanmar M of H and S, ICF. Myanmar Demographic and Health Survey 2015-16 [Internet]. Nay Pyi Taw, Myanmar, and Rockville, Maryland USA: Ministry of Health and Sports and ICF; 2017 Mar [cited 2021 Oct 28]. Available from: https://dhsprogram.com/publications/publication-fr324-dhs-final-reports.cfm

20. Niport NI of PR and T, ICF. Bangladesh Demographic and Health Survey 2017-18 [Internet]. Dhakar, Bangladesh, and Rockville, Maryland, USA: NIPORT and ICF; 2020 Oct [cited 2021 Oct 28]. Available from: https://dhsprogram.com/publications/publication-FR344-DHS-Final-Reports.cfm

21. Statistics/Cambodia NI of, Health/Cambodia DG for, International ICF. Cambodia Demographic and Health Survey 2014. 2015 Sep 1 [cited 2021 Oct 28]; Available from: https://dhsprogram.com/publications/publication-fr312-dhs-final-reports.cfm

22. National Institute of Preventive and Social medicine (NIPSOM). National STEPS survey for non-communicable diseases risk factors in Bangladesh 2018 [Internet]. Dhakar, Bangladesh: National Institute of Preventive and Social medicine (NIPSOM); 2018 [cited 2021 Oct 28]. Available from: https://apps.who.int/iris/handle/10665/332886

23. University of Health Science, Preventive Medicine Department of the Ministry of Health. Prevalence of non-communicable disease risk factors in Cambodia, STEPS Survey Country report 2010 [Internet]. Phnom Penh, Cambodia: University of Health Science, Preventive Medicine Department of the Ministry of Health; 2010 [cited 2021 Oct 28]. Available from: https://extranet.who.int/ncdsmicrodata/index.php/catalog/378

24. Lao People's Democratic Republic - STEPS 2013 [Internet]. [cited 2021 Oct 28]. Available from: https://extranet.who.int/ncdsmicrodata/index.php/catalog/588

25. National Statistical Office of Thailand. Thailand Multiple Indicator Cluster Survey 2019, Survey finding report [Internet]. Bangkok, Thailand: National Statistical Office of Thailand; 2020 [cited 2021 Oct 28]. Available from: https://www.unicef.org/thailand/reports/thailand-multiple-indicator-cluster-survey-2019

For peer review only

26. Lynch E, Falq G, Sun C, Bunchhoeung PDT, Huerga H, Loarec A, et al. Hepatitis C viraemic and seroprevalence and risk factors for positivity in Northwest Cambodia: a household cross-sectional serosurvey. BMC Infect Dis. 2021 Feb 26;21(1):223.

27. Komada K, Sugiyama M, Vongphrachanh P, Xeuatvongsa A, Khamphaphongphane B, Kitamura T, et al. Seroprevalence of chronic hepatitis B, as determined from dried blood spots, among children and their mothers in central Lao People's Democratic Republic: a multistage, stratified cluster sampling survey. Int J Infect Dis IJID Off Publ Int Soc Infect Dis. 2015 Jul;36:21–6.

28. Ashraf H, Alam NH, Rothermundt C, Brooks A, Bardhan P, Hossain L, et al. Prevalence and risk factors of hepatitis B and C virus infections in an impoverished urban community in Dhaka, Bangladesh. BMC Infect Dis. 2010 Jul 15;10:208.

29. Aye Aye Lwin, Khin Saw Aye, Moh Moh Htun, Yi Yi Kyaw, Ko Ko Zaw, Toe Thiri Aung, et al. Sero-prevalence of Hepatitis B and C Viral Infections in Myanmar: National and Regional Survey in 2015. Myanmar Health Sci Res J. 2017;29(3):167–75.

30. The EquityTool [Internet]. Equity Tool. [cited 2021 Oct 28]. Available from: https://www.equitytool.org/the-equity-tool-2/

31. Chakraborty NM, Fry K, Behl R, Longfield K. Simplified Asset Indices to Measure Wealth and Equity in Health Programs: A Reliability and Validity Analysis Using Survey Data From 16 Countries. Glob Health Sci Pract. 2016 Mar 21;4(1):141–54.

32. World Health Survey (WHS) [Internet]. [cited 2022 Apr 20]. Available from: https://apps.who.int/healthinfo/systems/surveydata/index.php/catalog/whs/about

33. van der Zouwen J, Smit JH. Evaluating Survey Questions by Analyzing Patterns of Behavior Codes and Question–Answer Sequences: A Diagnostic Approach. In: Methods for Testing and Evaluating Survey Questionnaires [Internet]. John Wiley & Sons, Ltd; 2004 [cited 2023 Sep 22]. p. 109–30. Available from: https://onlinelibrary.wiley.com/doi/abs/10.1002/0471654728.ch6

34. Presser S, Couper MP, Lessler JT, Martin E, Martin J, Rothgeb JM, et al. Methods for Testing and Evaluating Survey Questions. Public Opin Q. 2004 Mar 1;68(1):109–30.

35. Alemayehu S, Feghali KC, Cowden J, Komisar J, Ockenhouse CF, Kamau E. Comparative evaluation of published real-time PCR assays for the detection of malaria following MIQE guidelines. Malar J. 2013 Aug 8;12:277.

36. Hartung C, Lerer A, Anokwa Y, Tseng C, Brunette W, Borriello G. Open data kit: tools to build information services for developing regions. In: Proceedings of the 4th ACM/IEEE International Conference on Information and Communication Technologies and Development [Internet]. New York, NY, USA: Association for Computing Machinery; 2010 [cited 2023 Aug 23]. p. 1–12. (ICTD '10). Available from: https://doi.org/10.1145/2369220.2369236

For peer review only

37. Andrews JR, Barkume C, Yu AT, Saha SK, Qamar FN, Garrett D, et al. Integrating Facility-Based Surveillance With Healthcare Utilization Surveys to Estimate Enteric Fever Incidence: Methods and Challenges. J Infect Dis. 2018 Nov 10;218(suppl_4):S268–76.

38. Aiemjoy K, Seidman JC, Saha S, Munira SJ, Sajib MSI, Sium SMA, et al. Estimating typhoid incidence from community-based serosurveys: a multicohort study. Lancet Microbe. 2022 Aug 1;3(8):e578–87.

39. Falade-Nwulia O, Suarez-Cuervo C, Nelson DR, Fried MW, Segal JB, Sulkowski MS. Oral Direct-Acting Agent Therapy for Hepatitis C Virus Infection. Ann Intern Med. 2017 May 2;166(9):637–48.

40. Hepatitis B (HepB3) immunization coverage among 1-year-olds (%) [Internet]. [cited 2023 Aug 3]. Available from: https://www.who.int/data/gho/data/indicators/indicator-details/GHO/hepatitis-b-(hepb3)-immunization-coverage-among-1-year-olds-(-)

41. Thet Ssoe H. Myanmar Aging Survey. In: Gu D, Dupre ME, editors. Encyclopedia of Gerontology and Population Aging [Internet]. Cham: Springer International Publishing; 2021 [cited 2023 Aug 23]. p. 3375–8. Available from: https://doi.org/10.1007/978-3-030-22009-9_973

42. "The situation of Thailand's older population: An update based on the 2" by John KNODEL, Bussarawan TEERAWICHITCHAINAN et al. [Internet]. [cited 2023 Aug 23]. Available from: https://ink.library.smu.edu.sg/soss_research/1948/

43. Factors related to quality of life among older adults in Bangladesh: A cross sectional survey - Uddin - 2017 - Nursing & Health Sciences - Wiley Online Library [Internet]. [cited 2023 Aug 23]. Available from: https://onlinelibrary.wiley.com/doi/full/10.1111/nhs.12385

44. Jankhotkaew J, Chaiyasong S, Waleewong O, Siengsounthone L, Sengngam K, Douangvichit D, et al. The impact of heavy drinkers on others' health and well-being in Lao PDR and Thailand. J Subst Use. 2017;22(6):617–23.

45. DeSalvo KB, Fan VS, McDonell MB, Fihn SD. Predicting Mortality and Healthcare Utilization with a Single Question. Health Serv Res. 2005 Aug;40(4):1234–46.

46. Benyamini Y, Idler EL. Community Studies Reporting Association between Self-Rated Health and Mortality: Additional Studies, 1995 to 1998. Res Aging. 1999 May 1;21(3):392–401.

47. Makovski TT, Schmitz S, Zeegers MP, Stranges S, van den Akker M. Multimorbidity and quality of life: Systematic literature review and meta-analysis. Ageing Res Rev. 2019 Aug;53:100903.

48. MORU Tropical Network Policy on Sharing Data and Other Outputs [Internet]. [cited 2023 Oct 17]. Available from: https://www.tropmedres.ac/units/moru-bangkok/bioethics-engagement/data-sharing/moru-tropical-network-policy-on-sharing-data-and-other-outputs

For peer review only

Declarations

Authors' contribution

MZ, NSNH, TJP contributed to conception and design of the study, and drafted the manuscript.

SI, AS, MAI, AKN, RT, LD, CP, RC, EMB, WT, JW, SDB, SJL contributed to the design of the study, and provided critical review of the manuscript.

NW, CM contributed to the design of the study, data collection platform development, and provided critical review of the manuscript.

SIZ contributed to the design of the study, provided the study sites' map and critical review of the manuscript.

ML, RJM, NPJD, YL contributed to the conception of the study, and provided critical review of the manuscript.

Funding statement

This research was funded in whole, or in part, by the Wellcome Trust [215604/Z/19/Z]. For the purpose of Open Access, the author has applied a CC BY public copyright license to any Author Accepted Manuscript version arising from this submission.

Competing interests

Nothing to declare

Acknowledgements

The authors are grateful for the support from the local authorities in the participating countries. We acknowledge all staff from our partners and collaborators for their devoted contributions to the survey.

Tables and figures

Table 1. Required sample size tested against the estimated prevalence of key indicators of the highest prevalence, overall and by age groups of interests.

	**Expected number of participants**	**Indicator (estimated prevalence)**	**Required sample size ***
**Overall**	1500	Overweight (20%)	369
**Age groups (years)**			
≥15	1050	Current drinker (55%)	570
<5	150	Anemia (30%)	122
15-49	750	Injury or death caused by recent accident (20%)	414

For peer review only - http://bmjopen.bmj.com/site/about/guidelines.xhtml

≥50	300	Hypertension (20%)	257

*The formula used for calculating sample size is shown below. Assessing the sufficiency of the sample size for the key indicators of the highest prevalences ensures sufficient statistical power for indicators with lower prevalence. A degree of precision of 5% is applied for the sample size calculation overall and across age groups, except for the under 5 years and the 50 years or older groups, for which degrees of precision of 10% and 6% are applied, respectively.

$$n = DEFF \times \frac{Np(1-p)}{\frac{d^2}{1.96^2}(N-1) + p(1-p)}$$

n: Sample size.

DEFF: Design effect. Design effect of 1.5 is used to account for a slightly expected increase in variance due to clustering.

N: population size

p: expected prevalence of key indicators.

d: degree of precision.

For peer review only

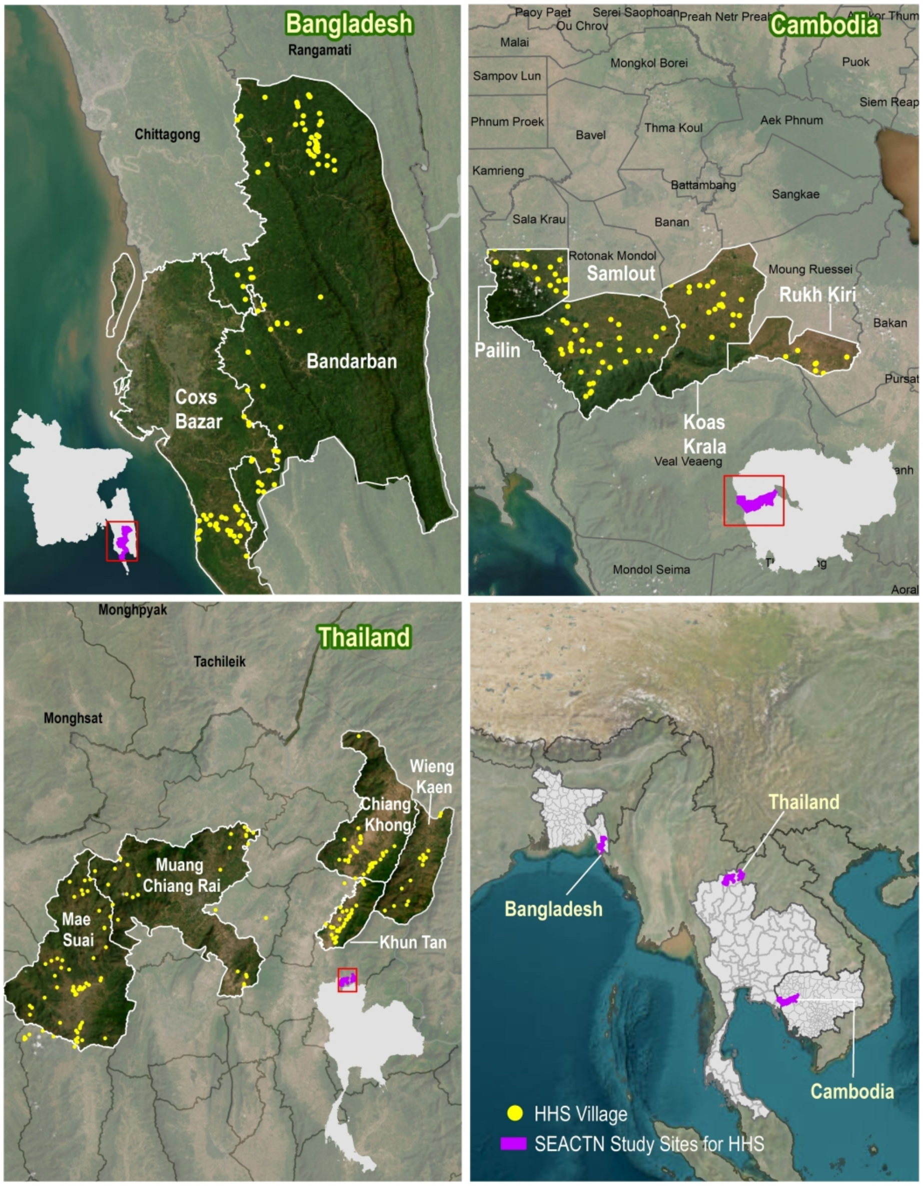

Figure 1. The study sites and population of the Cross-sectional Household Health Survey conducted by the South and Southeast Asia Community-based Trials Network (SEACTN).
The Bangladesh site includes 130 villages from Cox's Bazar and Bandarban districts; the Cambodia site includes 82 villages from Battambang and Pailin provinces; and the Thailand site includes 179 villages located in Chiang Rai province.
HHS: The Household Health Survey; SEACTN: the South and Southeast Asia Community-based Trials Network

218x279mm (300 x 300 DPI)

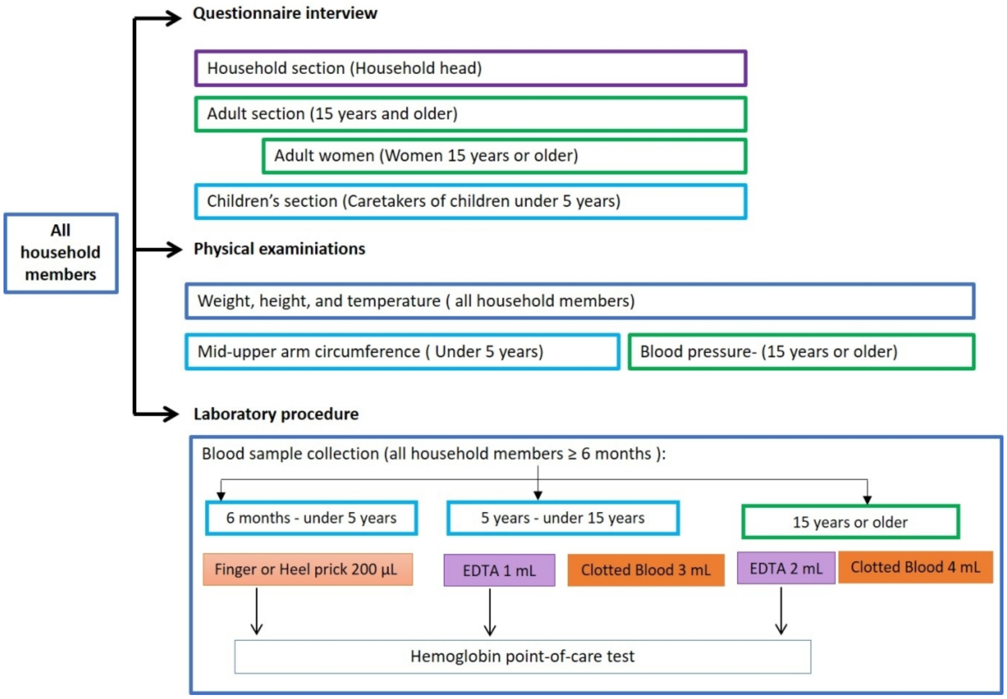

Figure 2. Questionnaire interviews, physical examinations, and laboratory procedures administrated to study participants according to age and sex.
EDTA: Blood sample collection tubes containing ethylenediaminetetraacetic acid.

230x161mm (150 x 150 DPI)

Appendix A. Questionnaire

DEFINING THE HIDDEN BURDEN OF DISEASE IN RURAL COMMUNITIES IN BANGLADESH, CAMBODIA AND THAILAND: A CROSS-SECTIONAL HOUSEHOLD HEALTH SURVEY PROTOCOL

Study site:

Village:

Household ID:

	Household head section		
Code	Question	Answer	Skip logic
S1a	(Household head or adult member of the household) Please give me the names of the persons who usually live in your household starting from the household head.<br><br>*Prompt: Have you told me the names of all the household members, even the ones are not here now?*<br><br>*Record the full name with the study ID in the paper-based household checklist*	Initials, and assign study ID automatically	

For peer review only - http://bmjopen.bmj.com/site/about/guidelines.xhtml

S1b	Could you now give me the names of the guests of the household who stayed here last night?		
S2	What is the relationship of [NAME] to the head of the household?	Head→S3<br>Wife or husband→S4<br>Son or daughter→S4<br>Son-in-law or daughter-in-law→S4<br>Grandchild→S4<br>Parent→S4<br>Parent-in-law→S4<br>Brother or sister→S4<br>Other relative→S4<br>Adopted/ Foster/ Stepchild →S4<br>Not related→S4<br>Don't know→S4	If S2=Head then S3, else skip to S4
S3	What is your ethnic group?	Ethnic group1<br>Ethnic group 2<br>[insert names of locally relevant ethnic groups]	
S4	Is [NAME] male or female?	Male<br>Female	
S5	Does [NAME] usually live here in the past month?	Yes<br>No	
S6	Did [NAME] stay here last night?	Yes<br>No	
S7	How old is [NAME]?	_______ in year<br><br>If age 5-14 years→S8-S10<br><br>Else→S1, until completing the information of all the mentioned household members→ S11	Go back to S1 until completing the information of household members.

For peer review only - http://bmjopen.bmj.com/site/about/guidelines.xhtml

S8	Did [NAME] attend school or any early childhood education program at any time during the [2021-2022] school year?	Yes<br>No→S10	If S8=No, then S10
S9	During [this/that] school year, what level [is/was] (NAME) attending?	Early childhood<br>Primary<br>Secondary<br>Higher<br>Don't know<br><br>→ S1, until the information of all household members is taken →S11	
S10	What Is the highest level of school [NAME] has attended?	Early childhood<br>Primary<br>Secondary<br>Higher<br>Don't know<br><br>→ S1, until the information of all household members is taken→ S11	Go back to S1 until completing the list

	Household Schedule summary		
Code	Question	Answers	Skip logic
S11	Total persons in household	Auto calculation	
S12	Number of household member >=18 years	Auto calculation	
S13	Number of household member 15-17 years	Auto calculation	
S14	Number of women household member>=15 years	Auto calculation	

For peer review only - http://bmjopen.bmj.com/site/about/guidelines.xhtml

S15	Number of school age children 5-14 years old	Auto calculation	
S16	Number of children under 5 years old	Auto calculation	
S17	Just to make sure that I have a complete listing: are there any other people such as small children or infants that we have not listed?	Yes →S1<br>No	If yes, go to S1
S18	Are there any other people who may not be members of your family, such as domestic servants, lodgers, or friends who usually live here?	Yes→S1<br>No	If yes, go to S1
S19	Are there any guests or temporary visitors staying here, or anyone else who stayed here last night, who have not been listed?	Yes→S1<br>No	If yes, go to S1
S20	Is there anybody, who is a household member or a guest who stayed in your household last night, is not here for the survey now?	Yes →S1<br>No →EQ1	
S21	Can you tell me the names of the people who are not here?	Choose from the Initials and study IDs	
S22	What are the reasons [name] is not here now?	Not available at the time (work or school)<br>Physical illness or disability<br>Mental illness or disability<br>Other reason, specify:______<br>Don't know	

	**Household section**		
Code	Question	Answers	Skip logic
	Now I'm going to ask you some questions about your household amenities and possessions.<br>[use Equity Tool of the country. Example Cambodia]		
	Does your household have		
EQ1	… Electricity	Yes<br>No	
EQ2	… a television?	Yes<br>No	
EQ3	… refrigerator?	Yes	

For peer review only - http://bmjopen.bmj.com/site/about/guidelines.xhtml

		No	
EQ4	…. CD or DVD player	Yes<br>No	
EQ5	… wardrobe?	Yes<br>No	
EQ6	…Generator or Battery or Solar panel	Yes<br>No	
EQ7	Does any member of your household own a motorcycle or scooter?	Yes<br>No	
EQ8	… A watch?	Yes<br>No	
EQ9	Does any member of this household have a bank account?	Yes<br>No	
EQ10	What is the main material of the floor?	Ceramic tiles<br>Wood planks<br>Other material	
EQ11	What is the main material of the exterior walls?	Cement blocks<br>Palm / bamboo / thatch<br>Other material	
	**Housing**		
HH1	What is the main source of drinking water for members of your household?<br><br>*(show card)*<br>*The showcard provided the definitions of the water source categories. Based on the description of the respondent, choose from the categories.*	Piped water<br>Piped into dwelling<br>Piped to yard/plot<br>Piped to neighbour<br>Public tap/standpipe<br>Tube well or borehole<br>Dug well<br>Protected well<br>Unprotected well<br>Water from spring<br>Protected spring<br>Unprotected spring	If "bottled water", then HH4

For peer review only - http://bmjopen.bmj.com/site/about/guidelines.xhtml

		Rainwater<br>Tanker truck<br>Cart with small tank<br>Surface water (river/dam/Lake/pond/stream/canal/Irrigation channel)<br>Bottled water→HH4<br><br>Other, specify:____	
HH2	Do you do anything to the water to make it safer to drink?<br><br>*Prompt: Participants might not know it's for making the water safer to drink. The interviewer can prompt if they do anything to the water before drinking.*	Yes<br>No → HH4<br>Don't know	
HH3	What do you usually do to make the water safer to drink? Anything else? (check all that apply)	Boil<br>Add bleach/chlorine<br>Strain through a cloth<br>Use water filter (ceramic/sand/composite/etc)<br>Solar disinfection<br>Let it stand and settle<br>Other, specify______<br>Don't know	
HH4	What kind of toilet facility do members of your household usually use?<br><br>*If the respondent answers in general terms such as "flush toilet," probe to determine where the toilet flushes to; likewise, if the respondent answers "latrine," probe to determine the type of latrine.*	Flush or pour flush toilet<br>Flush to piped sewer system<br>Flush to septic tank<br>Flush to pit latrine<br>Flush to somewhere else<br>Flush, don't know where<br><br>Pit latrine<br>Ventilated improved pit latrine	

For peer review only - http://bmjopen.bmj.com/site/about/guidelines.xhtml

	(show card)<br>*The show card provides you definitions of types of toilet, according to the description from the respondent choose the appropriate option.*<br>*If you cannot choose according to the participants description, show them the pictures on the showcard.*	Pit latrine with slab<br>Pit latrine without slab/open pit<br><br>Composting toilet<br>Bucket toilet<br>Hanging toilet/hanging latrine<br>No facility/bush/field<br><br>Other, specify _____________	
HH5	What type of fuel does your household mainly use for cooking?	Electricity<br>Liquefied Petroleum Gas (LPG)<br>Charcoal<br>Wood<br>No food cooked in household<br>Other (specify) ____________________	
HH6	Does the stove have a chimney or fan?	Yes<br>No<br>Don't know	
HH7	Is the cooking usually done in the house, in a separate building, or outdoors?	In the house<br>In a separate building<br>Outdoors<br>Other, specify:______	
HH8	Does your household have any mosquito nets?	Yes<br>No→ HH10	If HH8=No, then HH10
HH9	How many mosquito nets does your household have?	Number of net_______	
HH10	Do all of your household windows have mosquito/fly screens?	Yes<br>No	

For peer review only - http://bmjopen.bmj.com/site/about/guidelines.xhtml

ST1	How often is your house floor being cleaned (e.g. swept)?	Everyday (7 days/week)<br>Almost everyday (5-6 days/week)<br>Every other day (3-4 days/week)<br>Weekly (1-2 days/week)<br>Monthly (1-3 days/month)<br>Scarcely (a few times/ year)<br>Never	
ST2	How often do you or other members of your household perform activities to maintain grass or weed in or around your household, or work on a vegetable garden?<br><br>Prompt: For example, cut or trim weeds, plant or pick vegetables in the yard, garden, by the walls or fences of your household?	Everyday (7 days/week)<br>Almost everyday (5-6 days/week)<br>Every other day (3-4 days/week)<br>Weekly (1-2 days/week)<br>Monthly (1-3 days/month)<br>Scarcely (a few times/ year)<br>Never	
	**Household member check**		
	Now I'm going to asking you some questions about the members of your household including you, if anyone have had an accident, or had illness in the past 30 days.		
	**Accident and illness in the past 30 days**		
HM1	Was any person of your household injured or killed in an accident?<br>Prompt: anyone injured or killed in an accident any time before the interview.	Yes, how many? ___<br>No→HM14	If HM1=No, then HM14
HM2	What the name of the person got injured or killed in an accident?	Identify the ID code and show initial<br>Not in the list, initial:____	
HM3	Could you tell me in what type of accident (NAME) was injured or killed?	Landmine/unexploded bomb (UXO)<br>Gunshot/weapon<br>Road accident<br>Fire/burning<br>Snake/animal bite<br>Fall from tree/building<br>Drowning	

For peer review only - http://bmjopen.bmj.com/site/about/guidelines.xhtml

		Poisoning (chemical)<br>Violence/assault<br>Other, specify________<br>Don't know	
HM4	Is (NAME) still alive?	Yes<br>No→HM6	If HM4 =No, then HM6
HM5	In your opinion, was (NAME'S) injury serious, moderate, or slight?	Serious<br>Moderate<br>Slight<br>DON'T KNOW	
HM6	Was (NAME's) death due to accident?	Yes ⎤→ HM7<br>No ⎦	→HM7
HM7	Did the accident happen in the past 30 days?	Yes<br>No→HM14	If No then HM 14
HM8	Was advice or treatment sought for [Name] after the accident?	Yes<br>No→ HM14	If No then HM14
HM9	Where was advice or treatment first sought for [NAME] after the accident?	Public sector<br>Primary care unit/Primary care centers/Community Health centers<br>Community hospital<br>General hospital/Central hospital<br>University hospital<br>Government hospital<br>Private medical sector<br>Poly private clinic/private clinic<br>Private hospital<br>Others<br>source<br>Traditional/herbal medicine<br>Traditional healer<br>Self-bought medicine<br>Relatives/ Friends<br>, specify:________	

For peer review only - http://bmjopen.bmj.com/site/about/guidelines.xhtml

HM10	Where did the money come from to pay for transportation and treatment for the members of your household who had an injury over the past 30 days?<br><br>Record all that mentioned<br><br>*The payment method for transportation and medical care can be different. If the respond provided one method, the interviewer should probe and confirm if both payment for transportation and treatment are from the same method.*<br><br>*If any out-of-pocket payment method is selected, then proceed to HM11.*	Subsidy or exemption→ HM14<br>Insurance scheme →HM14<br>Universal coverage card (UC)<br>Social Security/ worker compensated fund (SSSS)<br>civil servant medical benefit scheme (CSMBS)<br>State enterprise<br>Independent agency of the state<br>Local government<br>Private health insurance<br>Health insurance covered by employer<br>Other health insurance, specify_____________<br><br>Out of pocket → HM11<br>Wage/income<br>Loan/borrowed money<br>Sale of assets<br>Gift from relatives/neighbours/friends<br>Saving/ family funds<br><br>Other, specify________→HM14<br>Don't know→HM14	
HM11	How much in total was spent on [NAME]'s treatment at (name of the place)?	________amount of money<br>Don't know	If HM10 checked out of pocket payment.
HM12	How much in total was spent on transport to go to and return from (name of the place)?	_____amount of money<br>Don't know	If HM10 checked out of pocket payment.
HM13	Has the debt been repaid?<br>*(If HM10= Loan/borrowed money)*	Yes<br>No<br>Don't know	If HM10= Loan/borrowed money
HM14	Please tell me if any member of your household (including you) is sick, has an illness now or at any time in the last 30 days?	Yes, how many? _________<br>No → End of the household head section	If HM14=No, then end of the section

For peer review only - http://bmjopen.bmj.com/site/about/guidelines.xhtml

	It can be **acute** disease or previous diagnosed **chronic** disease.<br>An acute illness is a condition that appears suddenly: the person did not have it immediately before becoming ill.<br>A chronic disease is an illness that will not go away or takes a long time to go away, even when treated.<br><br>Prompt if nobody:<br>Is there anyone had fever?<br>Any infant or child in your family was ill?		
HM15	Now I would like to ask you some questions about each person who is sick now or at any time in the <u>last 30 days</u>.<br><br>Could you tell me his/her/their name(s)? Then we will talk about one person at a time.	Identify all that from the list of the family member (ID number)<br><br>No body→ end of the household head section	
HM16	What type of health problems or symptoms did [name] have or is having during this illness?<br><br>DO NOT READ. TICK ONE BOX FOR EACH GROUP OF SYMPTOMS MENTIONED.<br><br>*If the respondent provide a diagnosis directly, especially for an acute condition, please still probe for the symptoms. Check all the symptoms that apply, and check "other acute condition, specify: ____ and write down the diagnosis.*<br>*For example: if the respondent answer " Covid", check "other acute condition, specify: COVID". Then continue probe what did the symptoms the person have, and check all the symptoms that are described.*	<u>Acute conditions</u><br>Fever<br>Persistent headaches<br>Persistent cough<br>Running nose<br>Difficulty breathing, fast breathing<br>Difficulty swallowing/ throat pain<br>Difficulty seeing or other eye complaint<br>Stomach pain, nausea, vomiting, could not eat<br>Thirst/ sweating<br>Watery diarrhoea<br>Bloody stools<br>Skin disease, such as rush, irritation, open sores<br>gyn-obstetrics<br>Could not sleep<br>Feeling tired<br>Constipation<br>Convulsion/ seizure	

For peer review only - http://bmjopen.bmj.com/site/about/guidelines.xhtml

		Musculoskeletal pain (general body pain, back and joint pain)<br><br><u>Chronic disease</u><br>Hypertension, high blood pressure<br>Heart disease, heart attack consequence<br>Diabetes, high blood sugar<br>Asthma, wheezing, chronic difficulty breathing<br>HIV infection, AIDS<br>Arthritis, chronic body pain<br>High cholesterol<br>Ulcer, chronic stomach pain<br>Stroke consequence<br>Epilepsy, seizures, fits<br>Cancer<br>Tuberculosis<br>Liver disease<br>Depression<br><br>Other acute illness, specify: _______<br>Other chronic illness, specify:________<br>Don't know	
HM17	In your opinion, was [NAME]'s illness serious, moderate, or slight?	Serious<br>Moderate<br>Slight<br>Don't know	
HM18	Was advice or treatment sought for [Name]'s illness?	Yes<br>No→End of the household head section	If HM18=No, then end of the section
HM19	Where was advice or treatment first sought for [NAME]'s illness?	<u>Public sector</u><br>Government hospital<br>Government health centre<br>Government health post	

For peer review only - http://bmjopen.bmj.com/site/about/guidelines.xhtml

	[insert Country specific list]	Mobile clinic<br>Community health worker/field worker<br>Other public sector<br>Private medical sector<br>Private hospital<br>Private clinic<br>Pharmacy<br>Private doctor<br>Mobile clinic<br>Community health worker/field worker<br>Other private medical sector<br>NGO medical sector<br>NGO hospital<br>NGO clinic<br>Other NGO medical<br><br>Other source<br>Shop<br>Traditional practitioner<br>Market<br>Itinerant drug seller<br><br>Other, specify:_______	
HM20	Where did the money come from to pay for transportation and medical care for the members of your household who had an illness over the past 30 days? (record all that mentioned)<br><br>*The payment for transportation and medical care can be different. If the respond provided one method, the interviewer should probe and confirm if both payment for transportation and treatment are from the same method.*	Subsidy or exemption→end of the section<br><br>Insurance scheme→ end of the section<br>Mutual Health Organization/Community-Based Health Insurance<br>Health Insurance through Employer<br>Social Security<br><br><br>Out of pocket → HM21<br>Wage/income	

For peer review only - http://bmjopen.bmj.com/site/about/guidelines.xhtml

	If any out-of-pocket method is recorded, proceed to HM21.<br><br>[Insert locally relevant insurance schemes]	Loan/borrowed money<br>Sale of assets<br>Gift from relatives/neighbours/friends<br>Saving/ family funds<br>specify___________--> End of the household head section<br>Don't know → End of the household head section	
HM21	How much in total was spent on [NAME]'s treatment at (name of the place)?	________amount of money<br>Don't know	If HM20 checked any out of pocket payment.
HM22	How much in total was spent on transport to go to and return from (name of the place)?	_____amount of money<br>Don't know<br><br>➔ HM23, if HM20=loan/borrowed money.<br>➔ Else, end of the household head section	HM23, if HM20=loan/borrowed money.<br>Else, end of the household head section
HM23	Have this debt been repaid?	Yes<br>No<br>Don't know	If HM20=Loan/borrowed money

For peer review only - http://bmjopen.bmj.com/site/about/guidelines.xhtml

	**Adult section**		
Code	Question	Answers	Skip logic
	**Respondent background**		
A1	How old were you at your last birthday?	Age in complete years __	
A2	Have you ever attended school?	Yes<br>No → A4	If no, then A4
A3	What is the highest level of school you completed?	No formal schooling<br>Less than primary school<br>Primary school uncompleted<br>Primary school completed<br>Secondary school completed<br>High school/ vocational school completed<br>College/university completed<br>Postgraduate degree	
A4	What is your marital status?	Never married<br>Currently married<br>Separated<br>Divorced<br>Widowed<br>Cohabitating<br>Refuse to answer	

For peer review only - http://bmjopen.bmj.com/site/about/guidelines.xhtml

A5	Which of the following best describes your main work status over the past 12 months?	Government employee<br>Non-government employee<br>State enterprise officer<br>Private company employee<br><br>Business<br>Business owner<br>Household business assistance<br>Agriculture<br>Agriculture (landowner and farmer)<br>Agriculture labourer (other's land)<br>Labourer<br>Industrial labourer<br>Day labourer<br>Transport labourer<br>Paid domestic worker<br>Blacksmith/ Goldsmith/other skilled labour<br>Other self-employed<br>Student<br>Home maker/household work<br>Retired<br>Unemployed<br>Unemployed (able to work, e.g. unwilling to work)<br>Unemployed (unable to work, e.g. too sick, disabled)<br><br>Other, specify:_________<br>Refuse to answer	
	Now, I'm going to ask you some questions about your behaviour when you are in or around your household, and when you are working, in the past 12 months.		
ST3	In the past 12 months, how often do you sit or lying directly on the floor in your house or lawn?	Everyday (7 days/week)<br>Almost everyday (5-6 days/week)	

For peer review only - http://bmjopen.bmj.com/site/about/guidelines.xhtml

		Every other day (3-4 days/week)<br>Weekly (1-2 days/week)<br>Monthly (1-3 days/month)<br>Scarcely (a few times/ year)<br>Never	
ST4	In the past 12 months, have you worked in forest or high hill (for collecting wood/ bamboo/ mushroom), or rice field, fruit/tea/coffee plantation, or dry farm field for other agriculture products (e.g. vegetable, corn, pineapple)?<br><br>*Prompt: the participant might have worked in different areas throughout the year, and worked different length in different area, prompt to record all areas that the participate have worked in the past 12 months.*	Yes<br>No→WHS1	If no, then WHS1
ST5	Which one of them have you worked in?<br>*Check all that apply*	Forest or high hill<br>Rice field<br>Fruit/Coffee/tea plantation<br>Dry field	Completing ST6-ST8 for each area checked
ST6	Do you work in the [areas checked above] all year or only when in some seasons?<br><br>*Check "all year" if worked every month in the past 12 months. The respondent might work with different frequency in different month, but if he/she has worked in the area in each month in the past 12 month, this option should be checked.*<br>*Check "in season" if not every month in the past 12 month.*	All year→ST8<br>In season →ST7	

For peer review only - http://bmjopen.bmj.com/site/about/guidelines.xhtml

ST7	How many months have you worked in [area] in the past 12 months?<br><br>*The number of months should be cumulative including all the months in the past 12months the respondent has worked in the area. E.g. if the participants worked in March-June, and November, the total months he/she worked in the area is 4 months, "3-5 months" should be checked.*	9-11 months<br>6-8 months/year<br>3-5 months/year<br>1-2 months/year or less	IfST6= In season
ST8	How often do you work in [area] (when it was the months you worked there)?<br><br>*Ask the question accordingly: if the respondent answered only work in the area in some seasons, then read the words in "(when it was the months you worked there)". If different frequency in different month, please use an average.*	Everyday (7 days/week)<br>Almost everyday (5-6 days/week)<br>Every other day (3-4 days/week)<br>Weekly (1-2 days/week)<br>Monthly (1-3 days/month)<br>Scarcely (a few times/ year)<br><br>➔ ST6, until completed all the areas the participants have listed	ST6 until completed the frequency for all the fields the respondent has worked in the past 12 months.
ST9a	The following questions we will ask you some things you do when you were at work in the area(s) you mentioned before. When you answer them, you need to consider: (all)the area(s) you have mentioned, and the total time you have worked in those area(s) in the past 12 months.<br><br>*Repeat the area(s) and the frequencies of working in the area from ST5-ST8*	Show the area(s) and frequency from ST5-ST8	
ST9	At the time when you work in the area(s), how often do you sit or lay directly on the ground, haystacks, grass or other places in the forest or high hill, or farm field without a mat?	Every time<br>Most times<br>Sometimes<br>Rarely<br>Never	

For peer review only - http://bmjopen.bmj.com/site/about/guidelines.xhtml

ST10	How often do you wear long sleeves?	Every time<br>Most times<br>Sometimes<br>Rarely<br>Never	
ST11	How often do you wear gloves?	Every time<br>Most of times<br>Sometimes<br>Rarely<br>Never	
ST12	How often do you wear shoes with socks or boots without or without socks?	Every time<br>Most of times<br>Sometimes<br>Rarely<br>Never	
ST13	How often do you wash your body after work?	Every time<br>Most of times<br>Sometimes<br>Rarely<br>Never	
ST14	How often do you change your clothes wore at work to clean clothes <u>immediately (i.e., within 30 minutes)</u> when you arrive home?	Every time<br>Most of times<br>Sometimes<br>Rarely<br>Never	
WHS1	I'm going to ask you now your overall health, including both your physical and your mental health.<br><br>In general, how would you rate your health <u>today</u>? Would you say it's very good, good, moderate, bad, or very bad?	Very good<br>Good<br>Moderate<br>Bad<br>Very Bad	

For peer review only - http://bmjopen.bmj.com/site/about/guidelines.xhtml
For peer review only

WHS2	The following questions, I'm going to ask you how much difficulty you have when doing some activities. You can give me answers, such as you have no difficulty, mild difficulty, moderate difficulty, or extreme difficulty, or cannot do it at all.<br><br>Overall in the last 30 days, how much difficulty did you have with work or household activities?	None<br>Mild<br>Moderate<br>Severe<br>Extreme/ Cannot do	
WHS3	Overall in the last 30 days, how much difficulty did you have with moving around, such as walking or climbing steps	None<br>Mild<br>Moderate<br>Severe<br>Extreme/ Cannot do	
WHS4	Overall in the last 30 days, how much difficulty did you have with selfcare, such as washing or dressing yourself?	None<br>Mild<br>Moderate<br>Severe<br>Extreme/ Cannot do	
WHS5	Overall in the last 30 days, how much of bodily aches or pains did you have?	None<br>Mild<br>Moderate<br>Severe<br>Extreme	
WHS6	Overall in the last 30 days, how much difficulty did you have with concentrating or remembering things?	None<br>Mild<br>Moderate<br>Severe<br>Extreme	
WHS7	Do you wear glasses or contact lenses?	Yes<br>No	

For peer review only - http://bmjopen.bmj.com/site/about/guidelines.xhtml

WHS8	In the last 30 days, how much difficulty did you have in seeing (IF WHS7=Yes, even when you wear glasses or contact lenses)?	None<br>Mild<br>Moderate<br>Severe<br>Extreme	
WHS9	Overall in the last 30 days, how much of a problem did you have with sleeping, such as falling asleep, waking up frequently during the night or<br>waking up too early in the morning?	None<br>Mild<br>Moderate<br>Severe<br>Extreme	
WHS10	Overall in the last 30 days, how much of a problem did you have with feeling sad, low, or depressed?	None<br>Mild<br>Moderate<br>Severe<br>Extreme	
	**Substance use**		
SU1	Now I would like to ask you some questions on smoking and tobacco use.<br>Do you currently smoke any tobacco products, such as cigarettes, cigar, or pipes?	Yes<br>No→S6	If No, then S6
SU2	Do you currently smoke tobacco product daily?	Yes<br>No	
SU3	How old were you when you first started smoking?	Age ______years→ S5<br>Don't know→ S4	If S3=Don't know, then S4, else S5
SU4	Do you remember how long ago it was?	__________in year/month/week<br>Don't know	
SU5	What type of tobacco do you currently smoke? (Check all that apply)	Manufactured cigarettes<br>Hand-rolled cigarettes<br>Kreteks<br>Pipes full of tobacco<br>Cigars, cheroots, or cigarillos<br>Water pipe (e.g. Baraku/ Hookah/ Shisha)<br>Other, specify:____	Jump to S10

For peer review only - http://bmjopen.bmj.com/site/about/guidelines.xhtml

		→ Select any option, move to S10	
SU6	In the past, did you ever smoke any tobacco products?	Yes<br>No→ S10	If S6=No, then S10
SU7	In the past, did you ever smoke tobacco product daily?	Yes<br>No	
SU8	How old were you when you first stopped smoking	Age ______years→S10<br>Don't know→S9	If S8=Don't know, then S9, else S10
SU9	Do you remember how long ago it was?	__________in year/month/week<br>Don't know	
SU10	Now I would like to ask you some questions about drinking alcohol.<br>Have you ever consumed any alcohol, such as beer, wine, spirits, or [ADD OTHER LOCAL EXAMPLES]?	Yes<br>No→S18	If No, then S18
SU11	We count one drink of alcohol as one can or small bottle of beer, one glass of wine, one shot of spirits, or [insert local example].<br><br>(Show card)<br><br>During the past 12 months, how frequently have you had at least one standard alcoholic drink?<br><br>*Refer to locally relevant examples of one standard drink.*	Daily<br>5-6 days per week<br>3-4 days per week<br>1-2 days per week<br>1-3 days per month<br>Less than once a month<br>Never	If S11=Never, then AS18
SU12	Have you consumed any alcohol in the past 30 days?	Yes<br>No→S18	If S12=No then S18
SU13	During the past 30 days, on how many occasions did you have at least one standard alcoholic drink?	Number_____<br>Don't know	

For peer review only

For peer review only - http://bmjopen.bmj.com/site/about/guidelines.xhtml

SU14	In the past 30 days, how many standard drinks on average did you have during one drinking occasion?<br><br>(Showcard)<br>*If the respondent is not able to reply by standard drink, ask on average the amount and type of alcohol he/she consume, and convert by yourself to number of standard drinks.*	Number of drinks_____<br>Don't know	
SU15	In the past 30 days, how many occasions did you have six or more standard alcoholic drinks in a single drinking occasion?<br><br>(Showcard)<br><br>*Prompt: Six standard drinks is roughly about: 6 cans of beer/3 big bottles of beer/ 6 shots of spirit/ a bottle of spirit shared among 3-4 people [insert local examples].*	Number of occasions____<br>Don't know	
SU16	In the past 30 days, did you consume any home brewed alcohol, any alcohol brought over the border/from another country, any alcohol not intended for drinking or other untaxed alcohol?	Yes<br>No→S18	If S16=No then S18
SU17	How many occasions did you consume any home brewed alcohol, any alcohol brought over the border/from another country, any alcohol not intended for drinking or other untaxed alcohol?	Number of occasions___<br>Don't know	
SU18	Do you currently chew betel nuts?	Yes<br>No	If S18=No then S20
SU19	In the last 24 hours, many pieces did you chew?	___pieces	
SU20	Have your ever used heroin, Yama/ice, Ecstasy, Glue, Marijuana, Cocaine, Histamine, Pantazogon, Valium/diazepam, [locally popular drug]?	Yes<br>No<br>Refuse to answer	If No or refuse to answer then D1

For peer review only - http://bmjopen.bmj.com/site/about/guidelines.xhtml

SU21	In the past 12 months have you used any of the drugs?	Yes<br>No<br>Refuse to answer	
S22	Have you ever injected drugs?	No<br>Yes, in past 12 months<br>Yes, before past 12 months<br>Refuse to answer	
	**Disease and symptom**		
D1	Have you ever been diagnosed with chronic lung disease (emphysema, bronchitis, COPD) by a doctor or other health worker?	Yes<br>No→D3	If No, then D3
D2	In the last 30 days, have you been taking any medication or other treatment (like oxygen) for it prescribed by a doctor or other health worker?	Yes<br>No	
D3	In the last 12 months, have you had a tuberculosis (TB) test? I mean, has a doctor examined your sputum (taken a sample of the substance spit out from a deep cough and sent it to a laboratory for analysis) or made an x-ray of your chest?	Yes<br>No→D5	If no, then D5
D4	Have you been taking any medication or treatment during the last 30 days prescribed by a doctor or other health worker?	Yes<br>No	
D5	Have you had fever in the past 30 days?	Yes<br>No→D7	If No, then D7
D6	How long have you had fever?	One week or more<br>No or less than one week	
D7	Are you currently coughing?<br><br>Prompt:<br>If participants answer induced coughing due to environmental factor or substance use, prompt: I mean	Yes<br>No→10	If No, then D10

For peer review only - http://bmjopen.bmj.com/site/about/guidelines.xhtml

	coughing throughout the day, even if you are not smoking or in the environment.		
D8	What is the duration of coughing continuously?	Two weeks or more<br>Less than two weeks	
D9	Are you coughing up blood?	Yes<br>No	
D10	Have you lost body weight unintentional/ suddenly?	Yes<br>No	
D11	In the past 30 days, have you experienced drenching night sweats?	Yes<br>No	If D5-D11 checked 2 or more symptoms, or coughing up blood, then refer to TB care
D12	Have you been diagnosed with Asthma (an allergy respiratory disease) by a doctor or other health worker?	Yes<br>No→D14	If No, then D14
D13	In the last 30 days, have you been taking any medication or treatment prescribed by a doctor or other health worker?	Yes<br>No	
D14	Have you ever had your blood pressure measured by a doctor or other health worker?	Yes<br>No->D17	If No, then D17
D15	Have you ever been told by a doctor or other health worker that you have raised blood pressure or hypertension?	Yes<br>No→D17	If No, then D17
D16	In the past 30 days, have you taken any drugs (medication) for raised blood pressure prescribed by a doctor or other health worker?	Yes<br>No	

For peer review only - http://bmjopen.bmj.com/site/about/guidelines.xhtml

D17	Have you ever had your blood sugar measured by a doctor or other health worker?	Yes<br>No→D20	If No, then D20
D18	Have you ever been told by a doctor or other health worker that you have raised blood sugar or diabetes?	Yes<br>No→D20	If No, then D20
D19	In the past 30 days, have your taken any drugs (medication) or insulin injection for diabetes prescribed by a doctor or other health workers?	Yes<br>No	
D20	Have you ever had your cholesterol (fat levels in your blood) measured by a doctor or other health worker?	Yes<br>No→D23	If No, then D23
D21	Have you ever been told by a doctor or other health worker that you have raised cholesterol?	Yes<br>No→D23	If No, then D23
D22	In the past 30 days, have you taken any oral treatment (medication) for raised total cholesterol prescribed by a doctor or other health worker?	Yes<br>No	
D23	Have you ever had a heart attack or chest pain from heart disease (angina) or a stroke (cerebrovascular accident or incident)?	Yes<br>No	
D24	Are you currently taking medications regularly to prevent or treat heart disease?<br>(for example: aspirin, statins- Lovastatin/ Simvastatin/ Atorvastatin/ any other statin)	Yes<br>No	
D25	Besides the health conditions we have just spoken about, do you currently have other chronic diseases that have been diagnosed by a doctor or health care provider?<br><br>(check all that apply)	No other disease<br>Cancer<br>Liver cancer<br>bronchial and lung cancer<br>Colon and rectal cancer	If mention the disease or symptoms above, go back to the relevant question

For peer review only - http://bmjopen.bmj.com/site/about/guidelines.xhtml

	If diseases that are mentioned are not in the list, but you know in which system it belongs, check "other, specify" in that system and fill the name of the disease.<br><br>*If you do not know which system the disease should be categorized, then check "Other unknow system disease, specify:_______" on the bottom of the list, and fill in the name of the disease.*	Breast cancer<br>Cervical cancer<br>Leukaemia/lymphoma<br>Other, specify:<br>Endocrine system and metabolic disorder<br>thyroid/goitre<br>Other, specify:________<br>Sensor disorders<br>Glaucoma<br>Cataract<br>Hearing loss<br>Other, specify<br><br>Cardiovascular / circulatory disease<br>Enlarged heart<br>congestive heart<br>heart fluttering<br>Cardiac arteriosclerosis<br>Severe cerebrovascular disease<br>Other, specify:____<br><br>Haematological disease<br>Thalassemia<br>Chronic respiratory disease<br>Chronic emphysema<br>Other, specify:_____________<br><br>Digestive disorders<br>Gastritis<br>Acid reflux<br>Liver cirrhosis<br>Other, specify:______<br><br>Genito-urinary disease	

For peer review only - http://bmjopen.bmj.com/site/about/guidelines.xhtml

		Kidney failure/kidney disabilities<br>enlarged prostate/inflammation<br>kidney stone/ bladder stone<br>Other, specify:___________<br><br><u>Muscular-skeleton disease</u><br>Gout<br>rheumatoid/ rheumatoid arthritis<br>Chronic knee pain / chronic back pain/ chronic neck pain<br>Paralysis<br>Other, specify:___________<br><br><u>Chronic infection or defective by infection disease</u><br>HIV<br>Polio<br>Leprosy<br>Chronic HBV infection<br>Chronic HCV infection<br>Other, specify:___________<br><br><u>Congenital anomalies</u><br>abnormal intelligence (Down syndrome)<br>Other, specify:___________<br><br><u>Oral disorders</u><br>Periodontitis (gingivitis)<br>Other, specify:___________<br><br><u>Immune system disorder</u><br>Allergy<br>Other, specify:___________	

For peer review only - http://bmjopen.bmj.com/site/about/guidelines.xhtml

For peer review only

		Neurological disorder<br>Parkinson's disease<br>Epilepsy/epilepsy<br>Dementia<br>Other, specify:___________<br><br>Mental health disorder<br>Alcoholism<br>Depression<br>Other, specify:__________<br><br><br>Other unknow system disease, specify:________<br><br>Unknown______	
CC0	You have told us the symptoms and diseases affect your health currently.<br>Now I'd like to ask you about the concerns you have related with health. It can be from the experience of yourself, your family, or your community.<br>I'm going to ask your concerns from different perspective of health, and in the end, I will ask you to rank the ones that concern you the most.		
CC1	The first 3 questions about different dimensions of health services.<br><br>I'm going to ask first if you have concerns about accessing health services, which means the service is provided somewhere, but you cannot receive it<br>Do you have any concerns about health service accessibility?	- Affordability- Cost of care<br>- Geographic accessibility- Travel to service provider (too far, take too much time, no good transportation methods, too sick to travel…)<br>- Language barrier<br>- Culture barrier<br>- Other, specify____________<br>- No concern	

For peer review only - http://bmjopen.bmj.com/site/about/guidelines.xhtml

	Prompt:<br>If the participant said no concern, then prompt service accessibility can be the cost, going to the place, or problem with communication, or something related with culture that create problem for people to get health care.<br><br>*Please record all that the participant mentioned.*		
CC2	The next question is about concerns of health service availability, which means the service is not provided, or some elements required for providing the service is absent or not enough<br>Do you have any concerns related with heath service availability?<br><br>Prompt:<br>If the participant expressed there is no concern, prompt- is there some health care service they want to use but cannot find the service? Or the service is not fully available because of health facility, health care professionals, equipment- laboratory, bed, ultrasound, or medication are absent or not enough.<br><br>*Please record all that the participant mentioned.*	- Physical presence of service (the service is not provided, or not provided all the time or at all. E.g. no dental care in the primary care unit)<br>- Health facility (health centre or hospital)<br>- Health care professionals (doctors/nurses/paramedical professionals)<br>- Beds or equipment<br>- Medication<br>- Other, specify:______<br>- No concern	
CC3	The next question is about your concerns on quality of health service, which means the service is available, and you are able to receive the service, but you are not satisfied with the service<br>Do you have concerns in the quality of health service?<br><br>Prompt:	- Safety: quality care should avoid harms to people whom the care is intended<br>- Timely: waiting time and delays<br>- Efficient: maximize the be benefit of available resources<br>- Other, specify______<br>- No concern	

For peer review only - http://bmjopen.bmj.com/site/about/guidelines.xhtml

	If the participant expressed no concern, prompt it can be related with safety, timely, or efficiency of the service provided.<br><br>*Please record all that the participant mentioned.*		
CC4	We have spoken about concerns related with health services, now Is like you to think about the environment around your household or at work that can potentially harm yours or others' health.<br><br>Do you have concerns related with your house, around your house, or things/ animals belong to your household, that could harm your health or other's health?<br><br>Prompt:<br>If the participant expressed no concern, prompt- it can be part of the structure of the house like wall, floor material; or something inside of the house related with health like toilet, drinking water source; or animals the household own; in the yard, garden of the household.<br><br>*Please record all that the participant mentioned.*	- House (e.g. wall, roof, floor)<br>- Amenities (e.g. toilet, cooking stove, mosquito net, drinking water)<br>- Animals (e.g. free range pigs in the yard)<br>- Other factors in and around the house, specify______<br>- No concern	
CC5	Do you think there are some risks to your or others' health related with work?<br><br>Prompt:<br>If the participant expressed no concern, prompt: for example, you often risk to get injured when you work, certain things/ products used at your work can harm your health.<br>If you work in the forest or in the rice farm, there are some insects or wild animal can harm you too.	- Risk to physical injuries<br>- Risk of chemical exposures<br>- Risk of exposure to nature environment (e.g. mosquito, snake bite)<br>- Other work-related risk factors, specify________<br>- No concern	

For peer review only - http://bmjopen.bmj.com/site/about/guidelines.xhtml

	Please record all that the participant mentioned.		
CC6	Do you have other concerns about health of yours or people around you?<br>*Please record all that the participant mentioned.*	Presence of disease or symptoms (respondent's or other people)<br>Other, specify________<br>No other concerns	
CC7	Thank you very much for sharing your concerns. You have told me your concerns are: [show results of CC1-CC6]<br>Now, could you (pick the biggest 3 concerns and) rank them? Start from the biggest concern.<br><br>*If the participant expressed less than 3 concerns, then rank the concern(s) mentioned, and select "NA" in the unused field.*	**Top1:________**<br>**Top2:________**<br>**Top3:________**	Automatic showing a list of options selected for CC1-CC6, and pick from the options.

For peer review only

For peer review only - http://bmjopen.bmj.com/site/about/guidelines.xhtml

	**Adult women section**		
Code	Question	Answers	Skip logic
W1	Now I would like to talk about family planning - the various ways or methods that a couple can use to delay or avoid a pregnancy. Have you ever heard of (METHOD)? (Check all that applied)	Condom<br>Pill<br>IUD<br>Injectables<br>Implants<br>Female condom<br>Emergency contraception<br>Standard days method<br>Lactational amenorrhea method<br>Rhythm method<br>Withdrawal<br>Female sterilization<br>Male sterilization<br>Other modern method, specify: _____<br>Other traditional method, Specify: __________<br>None<br>Refuse to answer	
W2	Are you currently pregnant?	Yes→W5<br>No or unsure	If yes, W5
W3	Are you or your partner currently doing something or using any method to delay or avoid getting pregnant?<br><br>Prompt: are you or your partner doing any of the following to avoid pregnancy: deliberately avoiding sex on certain days, using a condom, using withdrawal or using emergency contraception?	Yes<br>No→W5<br>Refuse to answer→W5	If No or Refuse to answer, then W5

For peer review only - http://bmjopen.bmj.com/site/about/guidelines.xhtml

W4	What are you currently using?<br><br>**<u>Do not read the answer, check all that applied.</u>**	None<br>Female sterilization<br>Male sterilization<br>IUD<br>Injectables<br>Implants<br>Pill<br>Condom<br>Female condom<br>Emergency contraception<br>Standard days method<br>Lactational amenorrhea method<br>Rhythm method<br>Withdrawal<br>Other modern method, specify:____<br>Other traditional method, specify:____	
W5	Now I would like to ask about all the birth you have had during your life. Have you ever given birth?<br>(if No prompt: including the times that a child was born alive but later died, or born dead)	Yes<br>No→ End of the section	If No, then end of the section.
W6	Now I would like to ask some questions about your last birth		
W7	How long ago it was?	_______years →W9<br>Don't know→W8	
W8	How old is the child now?	Age _____years<br>Don't know	If W7=don't know
W9	Did you see anyone for antenatal care for this pregnancy?	Yes<br>No→W12	If No, then W12
W10	Where did you receive antenatal care for this pregnancy?<br><br>Anywhere else?	<u>Home</u><br>Her home→W11<br>Other home→W11	If W10= Home/ others then W11

For peer review only - http://bmjopen.bmj.com/site/about/guidelines.xhtml

	Check all that apply<br><br>[Insert locally relevant list of health facilities]	Public sector →W12<br>Government hospital<br>Government health centre<br>Government health post<br>Other public sector<br><br>Private medical sector –>W12<br>Private hospitalPrivate clinic<br>Other private medical facility<br><br>NGO medical sector→ W12<br>NGO hospital<br>NGO clinic<br>Other NGO medical<br><br>Other, specify:_____-->W11	
W11	Whom did you see when you received the antenatal care at [her home/ other home]? Anyone else?<br><br>*Check all that apply*<br>Prompt: probe to have information on the health care worker's professional background. Distinguish among different health care professionals.<br><br>[insert locally relevant list]	Health personnel<br>Doctor<br>Nurse/midwife<br>Auxiliary midwife<br><br>Other person<br>Traditional birth attendant<br>Community health worker/<br>Field worker<br><br>Other, specify:__________	
W12	Where did you give birth to this child?	Home<br>Her home →W13<br>Other home→W13<br><br>Public sector →End of the section	

For peer review only - http://bmjopen.bmj.com/site/about/guidelines.xhtml
For peer review only

		Government hospital<br>Government health centre<br>Government health post<br>Other public sector<br><br>Private medical sector →End of the section<br>Private hospital<br>Private clinic<br>Other private medical sector<br><br>NGO medical sector<br>NGO hospital<br>NGO clinic<br>Other NGO medical<br>Other, specify:_____ -->W13	
W13	Who assisted with the delivery?<br><br>Prompt: probe to have information on the health care worker's professional background. Distinguish among different health care professionals.<br><br>Check all that apply	Health personnel<br>Doctor<br>Nurse/midwife<br>Auxiliary midwife<br><br>Other person<br>Traditional birth attendant<br>Community health worker/<br>Field worker<br><br>Other, specify:________	If W12= Home

For peer review only - http://bmjopen.bmj.com/site/about/guidelines.xhtml

	**Children's section**		
Code	Question	Answers	Skip logic
C1	We would like to ask you question about the [NAME]' health, including breastfeeding, vaccination history.<br>Are you willing to answer the questions?<br>Do you think you are able to answer the questions?	No, I don't want to/ or cannot answer the questions → C2<br>Yes, I'm willing to and able to answer the questions→C3	If No, then C2
C2	Could you tell us who is the best person to answer the questions about [Name] health?	Identify the member list, and ID	Restart from C1 with the person pointed
C3	Relationship of the person answering the question to the child	Mother<br>Father<br>Adopted/foster/ stepmother<br>adopted/foster/ stepfather<br>Grandmother<br>Grandfather<br>Sister<br>Brother<br>Other, specify:________	
C4	Has [NAME] ever been breastfed?	Yes<br>No→C7	If No, then C7
C5	Is [NAME] still breastfed?	Yes<br>No→7	If No, then C7
C6	Did (NAME) drink or eat anything other than breast milk yesterday or last night? Including plain water, other milk, non-milk liquid, or complementary food)	Yes<br>No	
	**Vaccination history**		**Only ask if 0-35 months child→ C7-C17, else C18**
C7	Now I would like to ask some questions about vaccinations received by [NAME].	Yes, has only a card<br>Yes, has only another document<br>Yes, has card and other document	

For peer review only - http://bmjopen.bmj.com/site/about/guidelines.xhtml

	Do you have a card or other document where (NAME)'s vaccinations are written down?	No, no card and no other document→C15	
C8	May I see the card or other document where (NAME)'s vaccinations are written down?	Yes, only card seen<br>Yes, only other document seen<br>Yes, card and other document seen<br>No card and no other document seen→C15	If NO then C16
C9	*Don't need to ask the question, compare the record in the vaccination card or document with vaccination schedule of Thailand. Check appropriate vaccines according to the child's age, and choose the option.*<br><br>*(show card)*<br>*Use the vaccination schedule in the show card to compare with the child's vaccination card*	Completed all age-appropriate vaccines→C12<br>Missing 1 dose age-appropriate vaccine<br>Missing more doses age-appropriate vaccines	If completed all age-appropriate vaccines, then C12
C10	Were age-appropriate doses of hepatitis B vaccination given according to the vaccination card or other document?	Yes, completed all age-appropriate doses – C12<br>Received the birth dose, but some doses missing according to children's age →C12<br>There is no record showing hepatitis B vaccination was given	
C11	At or soon after birth, did (NAME) receive a Hepatitis B vaccination, that is, an injection in the thigh to prevent Hepatitis B?	Yes<br>No<br>Don't know	If C11= There is no record
C12	Was a dose of vitamin A supplement given in the past 6 month according to the vaccination card or other document?	Yes →C14<br>No	
C13	In the past 6 months, did [Name] receive a vitamin A dose. (Show common type of ampules/capsules/syrups)	Yes<br>No<br>Don't know	If C13=No
C14	Ask the respondent for permission to photograph vaccination card or other document where vaccinations are written. If permission is granted, photograph card.	Photograph taken<br>Photograph not taken, permission not received<br>Photograph not taken, other reason	

For peer review only - http://bmjopen.bmj.com/site/about/guidelines.xhtml

		→ End of the section	
C15	Did (NAME) ever receive any vaccinations to prevent (NAME) from getting diseases, including vaccinations received in campaigns or immunization days or child health days?	Yes<br>No → C18<br>Don't know	If No/ don't know, then C18
C16	How many times did [Name] receive vaccination?	_____times<br>Reported completed all age-appropriate vaccine<br>Don't know	
C17	At or soon after birth, did (NAME) receive a Hepatitis B vaccination, that is, an injection in the thigh to prevent Hepatitis B?	Yes<br>No<br>Don't know	
C18	In the past 6 months, did [Name] received vitamin A dose.<br>Show common type of ampules/capsules/syrups)	Yes<br>No<br>Don't know	All under 5 years

The end of questionnaire

For peer review only - http://bmjopen.bmj.com/site/about/guidelines.xhtml

BMJ Open

DEFINING THE HIDDEN BURDEN OF DISEASE IN RURAL COMMUNITIES IN BANGLADESH, CAMBODIA AND THAILAND: A CROSS-SECTIONAL HOUSEHOLD HEALTH SURVEY PROTOCOL

Journal:	*BMJ Open*
Manuscript ID	bmjopen-2023-081079.R1
Article Type:	Protocol
Date Submitted by the Author:	21-Feb-2024

Complete List of Authors:	Zhang, Meiwen; Mahidol Oxford Tropical Medicine Research Unit, ; University of Oxford, Centre for Tropical Medicine and Global Health, Nuffield Department of Medicine<br>Htun, Nan Shwe Nwe; Mahidol Oxford Tropical Medicine Research Unit<br>Islam, Shayla; BRAC, Communicable Diseases Program<br>Sen, Aninda; BRAC, Communicable Diseases Program<br>Islam, Akramul; BRAC, Communicable Diseases Program<br>Neogi, Amit Kumer; BRAC, Communicable Diseases Program<br>Tripura, Rupam; Mahidol University, Mahidol-Oxford Tropical Medicine Research Unit, Faculty of Tropical Medicine<br>Dysoley, Lek; Cambodian National Malaria Control Program<br>Perrone, Carlo; Mahidol Oxford Tropical Medicine Research Unit<br>Chew, Rusheng; Mahidol Oxford Tropical Medicine Research Unit; University of Oxford, Centre for Tropical Medicine and Global Health, Nuffield Department of Medicine<br>Batty, Elizabeth M.; Mahidol Oxford Tropical Medicine Research Unit; University of Oxford, Nuffield Department of Medicine<br>Thongpiam, Watcharintorn; Mahidol Oxford Tropical Medicine Research Unit<br>Wongsantichon, Jantana; Mahidol Oxford Tropical Medicine Research Unit<br>Menggred, Chonticha; Mahidol Oxford Tropical Medicine Research Unit<br>Zaman, Sazid Ibna; Mahidol Oxford Tropical Medicine Research Unit; University of Oxford, Centre for Tropical Medicine and Global Health, Nuffield Department of Medicine<br>Waithira, Naomi; Mahidol Oxford Tropical Medicine Research Unit; University of Oxford, Centre for Tropical Medicine and Global Health, Nuffield Department of Medicine<br>Blacksell , Stuart; Mahidol Oxford Tropical Medicine Research Unit; University of Oxford, Centre for Tropical Medicine and Global Health, Nuffield Department of Medicine<br>Liverani, Marco; London School of Hygiene & Tropical Medicine, Department of Global Health and Development; Mahidol University, Faculty of Public Health<br>Lee, Sue; Mahidol Oxford Tropical Medicine Research Unit; University of Oxford, Centre for Tropical Medicine and Global Health, Nuffield Department of Medicine<br>Maude, Richard; Mahidol Oxford Tropical Medicine Research Unit; University of Oxford, Centre for Tropical Medicine and Global Health,

Nuffield Department of Medicine
Day, Nicholas; Mahidol Oxford Tropical Medicine Research Unit; University of Oxford, Centre for Tropical Medicine and Global Health, Nuffield Department of Medicine
Lubell, Yoel ; Mahidol Oxford Tropical Medicine Research Unit; University of Oxford, Centre for Tropical Medicine and Global Health, Nuffield Department of Medicine
Peto, Thomas; Mahidol Oxford Tropical Medicine Research Unit; University of Oxford, Centre for Tropical Medicine and Global Health, Nuffield Department of Medicine

**Primary Subject Heading**:	Epidemiology
Secondary Subject Heading:	Global health, Public health
Keywords:	INFECTIOUS DISEASES, Risk Factors, Chronic Disease, Quality of Life, EPIDEMIOLOGY

SCHOLARONE™
Manuscripts

DEFINING THE HIDDEN BURDEN OF DISEASE IN RURAL COMMUNITIES IN BANGLADESH, CAMBODIA AND THAILAND: A CROSS-SECTIONAL HOUSEHOLD HEALTH SURVEY PROTOCOL

Meiwen Zhang[1,2], Nan Shwe Nwe Htun[1], Shayla Islam[3], Aninda Sen[3], Md Akramul Islam[3], Amit Kumer Neogi[3], Rupam Tripura[1], Lek Dysoley[4], Carlo Perrone[1], Rusheng Chew[1,2,8], Elizabeth M. Batty[1,2], Watcharintorn Thongpiam[1], Jantana Wongsantichon[1], Chonticha Menggred[1], Sazid Ibna Zaman[1,2], Naomi Waithira[1,2], Stuart D. Blacksell[1,2], Marco Liverani[5-7], Sue J Lee[1,2,9], Richard J. Maude[1,2,10], Nicholas P.J. Day[1,2], Yoel Lubell[1,2], Thomas J. Peto[1,2]

1. Mahidol Oxford Tropical Medicine Research Unit, Bangkok, Thailand
2. Centre for Tropical Medicine and Global Health, Nuffield Department of Medicine, University of Oxford, Oxford, UK
3. Communicable Diseases Program, BRAC, Dhaka, Bangladesh
4. Cambodian National Malaria Control Program, Phnom Penh, Cambodia
5. Department of Global Health and Development, London School of Hygiene & Tropical Medicine, London, UK
6. Faculty of Public Health, Mahidol University, Bangkok, Thailand
7. School of Tropical Medicine and Global Health, Nagasaki University, Nagasaki, Japan
8. Faculty of Medicine, University of Queensland, Brisbane, Australia
9. Monash University and Alfred Hospital, Department of Infectious Diseases, Melbourne, Australia
10. The Open University, Milton Keynes, UK

Corresponding author: Meiwen Zhang

Corresponding author's email address: meiwen@tropmedres.ac

Abstract

Introduction

In low- and middle-income countries in Southeast Asia, the burden of diseases among rural population remains poorly understood, posing a challenge for effective healthcare prioritization and resource allocation. Addressing this knowledge gap, the South and Southeast Asia Community-based Trials Network (SEACTN) will undertake a survey that aim to determine the prevalence of a wide range of non-communicable and communicable diseases, as one of the key initiatives of its first project- the Rural Febrile Illness project (RFI). This survey, alongside other RFI studies that explore fever etiology, leading causes of mortality, and establishing village and health facility maps and profiles, will provide an updated epidemiological background of the rural areas where the network is operational.

Methods and analysis

During 2022-2023, a cross-sectional household survey will be conducted across three SEACTN sites in Bangladesh, Cambodia, and Thailand. Using a two-stage cluster-sampling approach, we will employ a probability-proportional-to-size sample method for village, and a simple random sample for household,

For peer review only - http://bmjopen.bmj.com/site/about/guidelines.xhtml

selection, enrolling all members from the selected households. Approximately 1500 participants will be enrolled per country. Participants will undergo questionnaire interview, physical examination and hemoglobin point-of-care testing. Blood samples will be collected and sent to central laboratories to test for chronic and acute infections, and biomarkers associated with cardiovascular disease, and diabetes. Prevalences will be presented as an overall estimate by country, and stratified and compared across sites and participants' sociodemographic characteristics. Associations between disease status, risk factors, and other characteristics will be explored.

Ethics and dissemination

This study protocol has been approved by the Oxford Tropical Research Ethics Committee, National Research Ethics Committee of Bangladesh Medical Research Council, the Cambodian National Ethics Committee for Health Research, the Chiang Rai Provincial Public Health Research Ethical Committee. The results will be disseminated via the local health authorities and partners, peer-reviewed journals, and conference presentations.

300/300

Trial registration

Clinicaltrials.gov, NCT05389540.

Strengths and limitations

- The study will utilize diverse methods (interviews, physical examinations, and laboratory tests) to gather extensive data on the prevalence, risk factors, healthcare utilization patterns of both communicable and non-communicable diseases
- The use of two-stage cluster sampling enables the sample to be representative of each study area.
- As an integrated component of the Rural Febrile Illness project, the findings will be synergized with outcomes of concurrent studies in these specified regions, offering a multidimensional overview of health and healthcare provision.
- The findings are specific to the selected rural areas, and should be interpreted with caution when considering broader implications for rural populations in the study countries.

Introduction

Life expectancy in Southeast Asia increased from 63 to 71 years between 2000 and 2019 (1). These positive changes can be attributed, in part, to advancements in the region's health systems, which have addressed traditional high-burden diseases, such as infectious diseases, maternal and neonatal health, and under 5 mortality (2). Despite these improvements, the burden of traditionally significant diseases persists, particularly in rural areas. Furthermore, there is a transition in disease epidemiology due to rapidly changing environments, growing economies, and ageing populations (3). The shift is marked by a rising burden of non-communicable diseases and injuries, along with emerging infectious diseases (e.g. COVID-19 and chikungunya) (4,5).

For peer review only

The knowledge of current disease epidemiology in the region, however, is limited, constraining health systems' ability to identify healthcare priorities and direct future resource allocation (6). While the Global Burden of Disease study provides insights into disease burdens across countries and diseases, the accuracy of its results is impeded by the scarcity of recent epidemiological data (6).. Given the limited coverage of disease reporting and surveillance systems in the region, research data is pivotal in bridging this information gap. Such data not only offers up-to-date information for modeling studies, but is also critical for optimizing resource allocation by including factors such as disease severity, the magnitude of the population affected, and equity (7).

Moreover, the marked systemic disparities in healthcare between rural and urban areas in the region highlight the need for rural-specific epidemiological data(3). Despite 50%- 85% of the region's population residing in rural areas, this large population group remains relatively understudied compared to countrywide estimates (8–13). In this context, cross-sectional surveys are an effective research method to provide timely estimates of disease prevalence in rural communities, through cost-effective approaches combining questionnaire interviews, clinical examinations, and laboratory tests.

The newly-formed South and Southeast Asia Community-based Trials Network (SEACTN) aims to establish a network of community-based healthcare providers and facilities capable of implementing interventions designed to triage, diagnose, and treat patients within rural communities across five South and Southeast Asian countries (Bangladesh, Cambodia, Laos, Myanmar, and Thailand) (14). The first project is the Rural Febrile Illness Project (RFI), dedicated to delineating the epidemiological baseline of febrile illness in remote and underserved regions, where febrile illness and access to healthcare pose significant health challenges (14). RFI encompasses diverse initiatives designed to gain a multifaceted understanding of the health dynamics within these communities to effectively facilitate the identification of interventions for future studies across SEACTN. Recognizing the gaps in understanding of disease prevalence, a cross-sectional household health survey has been planned as one of the key initiatives of the RFI.

This survey aims to define the prevalence of a broad spectrum of communicable and non-communicable causes of health conditions in areas where the network operates. Alongside the survey, other key initiatives of RFI include a fever etiology study to determine the incidence, causes, and outcomes of febrile illness; a verbal autopsy study to identify common causes of mortality and the circumstances surrounding death; and a village and health facility mapping study creating a detailed profile of the study villages, estimating travel time to health facilities, and identifying health service provision gaps. Each initiative holds significant value as an independent study, and their results will be complementary providing a thorough comprehension of healthcare needs and enabling more equitable resource allocation.

Methods and analysis

The SEACTN household health survey is a community-based cross-sectional survey aiming to provide an overview of the burden of disease in selected rural areas where the network is operational. The first participant was enrolled on October 3, 2022, and data collection is expected to continue until December, 2023.

For peer review only - http://bmjopen.bmj.com/site/about/guidelines.xhtml

Primary objectives and outcomes

1. To determine the prevalences of selected diseases and exposure to locally prevalent or important pathogens, including, but not limited to:
    a. Immunoglobulin G (IgG) against selected common pathogens causing fever.
    b. Hepatitis B and C
        i. Hepatitis B virus (HBV) surface antigen
        ii. IgG against Hepatitis C virus (HCV) and presence of HCV
    c. Selected non-communicable diseases (e.g. diabetes, raised blood cholesterol, hypertension, stroke) according to self-reported disease history, and/or physical examinations, and/or laboratory tests.
    d. Self-reported illness or injury in the past 30 days.
        i. Any acute conditions (e.g. fever, persistent cough, watery diarrhea)
        ii. Injury or death caused by an accident
2. To determine the point prevalence of different self-perceived health statuses.
3. To determine the prevalences of major risk factors for common non-communicable diseases (e.g. smoking, alcohol consumption, overweight).

Study population and setting

All SEACTN operational areas were selected by partner organizations as they represent poor, remote, and rural communities with limited access to formal healthcare (14). This study will be conducted in three areas, consisting of 391 villages in Bangladesh, Cambodia and Thailand (Figure 1). The other sites will not take part of the study due to operational constraints.

Two-stage cluster sampling will be used to obtain a representative sample from each study site. In the first stage, 75 villages will be selected using the probability-proportional-to-size sample method (15–17). For the second stage, within each village, a simple random sample of five to seven households (adjusted to the average household size) will be selected using computer-generated random sampling from the village household list.

Inclusion and exclusion criteria

For each selected household, all usual household members or visitors who stayed overnight in the households before the survey are eligible to be enrolled (18).

Sample size

This study is adequately powered to determine the prevalences of all key indicators within each age group, with a design effect of 1.5 and a range of 5-10% degrees of precision, while considering a maximum of 1500 participants per study country (Table 1). To account for a 10% non-response rate, assuming an average household size of 4.5 persons, and enrolling all household members, the final sample size was adjusted to 1667 participants per country (19–21).

The minimal sample size required was calculated based on the estimated prevalences of key indicators overall and within each population age group (Table 1). Prevalence estimates, population proportions within each age group, and average household size, were derived from previously conducted national health surveys and published studies, preferably from the study country, or from countries with a similar

For peer review only

epidemiological context (19–29). When the estimated prevalence of an indicator varied across study country, the highest prevalence estimates were applied.

Study procedures

Standard operating procedures (SOPs) were developed for field procedures, sample collection and transportation. Training materials were developed and training sessions were delivered to all the study staff directly, or using a train-the-trainer model where field supervisors and managers will be trained first and then subsequently train other field staff. All research staff are experienced in community-based research or interventions. Experienced phlebotomists were recruited and trained in specimen collection and processing in the field. A monitoring and evaluation plan was devised to maximize the research quality.

Patient and Public Involvement Statement

The potential challenges for the study include comprehensibility of the questionnaires, and willingness of selected households to participate. We have proactively addressed these concerns by involving the target population in the questionnaire validation process (section: Questionnaire interviews), and planning community mobilization events before the survey, tailored to the local context.

Collaborators will identify contact points from local stakeholders and communities, such as village leaders, local health center staff, or community health workers, and determine the most suitable methods for establishing contact. Main events may involve community engagement meetings to explain the study purpose, organization and procedures, and the plan for dissemination of study results. Prior to the survey day for each village, the study team will meet, or call if physical meetings are not feasible, the household head or members of the selected households. With the support of the key contact persons, this interaction will create an opportunity to address questions or concerns, enabling the survey teams to work with each community and selected household to plan an effective data collection schedule.

Survey schedule

On the scheduled survey day for each village, stations will be set up at a convenient location within or near the village. Household members will arrive at the survey location following their appointment to complete the procedures (Figure 2). If household members do not attend the data collection at the appointment time, they will be contacted and encouraged to join the survey at another time during the day, while the research team were in the village.

Informed Consent

Participants must sign the informed consent form before any study-specific procedures are performed. The informed consent will be available in the official local language. It will be presented to prospective participants by trained study staff detailing the study procedures and implications of study participation. It will be made explicit that participation is voluntary and participants are free to withdraw from the study at any time. If necessary, the informed consent process will be interpreted to dialect when participants do not speak the official local language.

Adequate time will be given to study participants to consider the information and ask questions. Written consent will then be obtained from the participants, or the caretakers of participants, in compliance with

For peer review only

local legal age requirements, using the participant's dated signature or thumbprint (if unable to write) and dated signature of a person who presented and obtained the informed consent. A copy of the signed Informed Consent document will be given to the participant. Children aged as dictated by local legislation and regulations will be required to sign the written informed assent form in addition to their parent or guardian signing a consent form.

Questionnaire interviews

The questionnaire consists mostly of questions adapted from well-validated and widely implemented tools (Appendix A). This approach enables direct comparison of results with previously conducted surveys by incorporating questions from the Demographic and Health Surveys (DHS), World Health Organization (WHO) STEPWise surveys for Non-communicable Disease Risk Factors (STEP), and the World Health Survey (30–32).

The questionnaire underwent adjustments and validation through pretests using behavior coding and participant debriefing (33,34). During mock interviews, the observer of each interview pair recorded the interaction problems for each question, such as "major change in wording", "question reading interrupted by the respondent", or "additional prompt is provided". All interviewers, interviewees, and the observers participated in the debriefing. The pretest was conducted initially among peer researchers in English. Subsequently, the adjusted version was translated into Thai and tested among the target population in Thailand. This ensured the questionnaire's suitability, comprehensibility, and appropriate length for administration. The final questionnaire was then translated into the official languages of each study country (Bangla, Khmer, and Thai) by professional translators or dedicated staff from partner organizations, and validated by the local research teams. When necessary, during face-to-face interviews, questions will be interpreted to dialect for participants who do not speak the official local language.

The questionnaire consists of four sections answered by household members based on their sex and age (Figure 2, top).

- The household section is completed by the household head, providing information on household characteristics, and a list of all household members along with any illnesses in the past 4 weeks and health seeking behavior.
- The adult section is for household members aged 15 years or older and includes questions on socio-demographic characteristics (e.g., age, sex, education, marital status, and occupation), disease history (e.g. tuberculosis, diabetes, cardiovascular disease), self-perceived health status, health concerns, substance use (e.g., tobacco and alcohol), and other disease risk factors.
- The women's health section is for female household members aged 15 years or older, and includes questions on contraception use, utilization of antenatal care and delivery services.
- The children's health section is answered by caretakers of children under 5 years, providing information on each child's vaccination and breastfeeding history.

Physical examination

All household members will undergo weight, height, and tympanic temperature measurements. Children under 5 years will also have their mid-upper arm circumference (MUAC) measured, and adult household members ($\geq$15 years) will undergo blood pressure measurement (Figure 2, middle). Weight will be measured with a digital scale, preferably with a taring function allowing a child's weight to be measured while being held by an adult. Height measurements will be carried out with measuring scales/boards.

For peer review only

Children younger than 24 months will be measured lying down on the board, while standing height will be measured for older children. Tympanic temperature will be measured with infrared thermometers.

All measures will be taken by experienced and trained staff. Consistency and comparability will be ensured by using the same validated devices previously used in studies in similar settings, whenever possible, across study sites.

Blood sample collection and point-of-care hemoglobin test

Blood samples will be collected from participants aged six months or older (Figure 2, bottom). For participants aged 6 months to under five years, four dried blood spots (DBS) will be collected via finger or heel prick. Participants aged five years or older will provide venous blood samples, with four milliliters collected from those aged five to under 15 years, and six milliliters from participants aged 15 years or older (Figure 2, bottom). After blood sample collection, appropriate samples will be tested for hemoglobin using HemoCue® Hb 301 system (HemoCue®AB, Ängelholm, Sweden) to detect anemia.

The venous blood samples will be temporarily stored in cool boxes between 4°C and 8°C, for a maximum of 24 hours before centrifugation in the field laboratories. Aliquots of whole blood, serum, plasma, and packed red blood cells of these samples will be stored at a minimum of -20°C or below without freeze-thaw until analysis. DBS will be air-dried in the laboratory for at least six hours, after which they will be placed in a plastic zip-lock bag and stored at room temperature or at 4°C or below.

Information, consultation and referral

Upon completion of the field procedures, individual participants will receive the results of their hemoglobin test and physical examinations. Participants with abnormal results will be referred to a study nurse who will provide recommendations and, if the participant wishes, further referrals in accordance with local guidelines.

Non-responders

Household members who fail to attend the survey (who are absent, refuse participation, or for other reasons) will have their demographic characteristics (e.g., sex, age) summarized from the information obtained from the household head in the household section of the questionnaire, to determine whether systematic differences exist between responders and non-responders.

Analysis of blood samples

Sample aliquots and DBS will be shipped to the central laboratories in Bangkok, Thailand where they will be analyzed. All laboratory activities will be performed by experienced and trained staff. All laboratory procedures will be performed using validated SOPs. Multiplex serology tests based on Luminex xMAP Intelliflex® System will be developed and validated for measures of IgG and immunoglobulin M (IgM) of common pathogens causing fever; all other methods have already been published in peer-reviewed journals.

Malaria quantitative polymerase chain reaction (qPCR) will be performed for all participants to identify individuals with malaria parasites. The PCR methods typically have a detection level in the range of 100-1000 parasites per milliliter(35). Genus-specific qPCR targeting Plasmodium *18S rRNA* genes will first be

performed to screen for positive individuals, and then species-specific assays will be performed to differentiate the parasite species (*Plasmodium falciparum*, *Plasmodium vivax*, *Plasmodium malariae*, *Plasmodium ovale*, and *Plasmodium knowlesi*) (36–39).

Serology tests for selected common pathogens causing fever including IgG and IgM for Dengue, Chikungunya, Japanese encephalitis, Zika, SARS-CoV-2, *Plasmodium vivax*, *Plasmodium falciparum*, *Orientia tsutsugamushi*, *Rickettsia spp*., and *Leptospira spp*. will be performed for all participants.

Tests for hepatitis B and C: all participants will be tested for HBV surface antigen; participants 15 years or older will also be tested for IgG for HCV, and the positive samples will undergo HCV PCR.

Non-communicable disease-related markers: participants 15 years or older will be tested for Hemoglobin A1c, total cholesterol and high-density lipoprotein cholesterol.

Additionally, the remaining samples will be tested for exposure to, or presence of, pathogens associated with selected diseases that are regionally or locally prevalent or important. The final list of diseases, pathogens, and sample selection will be adjusted based on findings from other SEACTN studies exploring diagnostics and prognostics in febrile patients, and other available evidence.

Results feedback

Individual-level data collected from physical examinations and hemoglobin tests will be provided on the same day once all field procedures are concluded for an individual participant (Section: Study procedure-Information, consultation and referral). Additionally, participants will be thoroughly informed about the implications of the laboratory tests to be performed during the consent process. They will be presented with the option to receive the results of hepatitis B and C tests. If they wish to receive them, the results, once available, will be placed in a sealed envelope and will then be delivered by the local health providers or village leaders to the participants. It is anticipated that there will be a delay of approximately six months to one-year from sample collection to results feedback. The timeframe encompasses the completion of data collection at the entire site, transportation of samples to Bangkok, sample management, analysis, and the subsequent return of results. The decision on which results to provide to individual participants is made in consultation with the site research teams and local clinicians, considering factors such as their clinical relevance, the potential harm that could result from a lack of consultation upon results distribution, and the accessibility of treatment.

Planned analysis

All data obtained through questionnaire interviews, physical examinations, and hemoglobin point-of-care tests will be collected on tablets using an electronic data collection platform - Open Data Kit (40). The electronic case report form has built-in validation rules to identify missing or potentially incorrect data. Stringent checks are applied to variables used to estimate the study outcomes, such as recent fever history, diabetes history, and weight. Survey interviewers cannot progress to the next question if these data are missing or outside the defined ranges. Throughout the survey, data will be continuously updated and monitored for quality. Data queries and quality reports will be generated every two to four weeks for data verification and correction, and to identify areas needing additional training and support, ensuring data integrity.

For peer review only

For descriptive summaries, means and standard deviations (SD) or medians with interquartile range (IQR) for continuous variables, and proportions for categorical variables will be calculated. Outcomes will be presented as an overall estimate by country, and with 95% confidence intervals, stratified and compared across sites and participants' sociodemographic characteristics using appropriate tests, such as the Student's t-test, Mann-Whitney U tests or chi-squared tests. Associations between disease status, risk factors, self-perceived health status, and other characteristics will be explored through univariate and multivariate analyses, and measures of effect will be reported with 95% confidence intervals. Sampling weights will be accounted for, as needed, in the analysis.

Discussion

This prevalence survey conducted across three rural SEACTN study sites in Bangladesh, Cambodia, and Thailand aims to provide a comprehensive epidemiological description of the study areas. Employing a multi-faceted approach, including questionnaire interviews, laboratory tests, and physical examinations, the study will yield extensive data on the prevalence, risk factors, healthcare utilization patterns of both communicable and non-communicable diseases. The findings will contribute to improved estimates of the burden of disease, and deepen the comprehension of findings from other RFI initiatives, offering a thorough overview of health and healthcare provision in these areas. The survey will be invaluable for setting healthcare priorities and directing resource allocation for health system development.

As a core component of RFI, this survey, alongside other initiatives, adopts an integrated approach to address health challenges more effectively, particularly in the context of the "double burden of disease", characterized by the rising burden of non-communicable diseases, alongside ongoing challenges posed by infectious diseases and sub-optimal maternal and child health (2). This survey will encompass various dimensions of health, allowing for further exploration of associations among disease occurrence, risk factors, and self-perceived health. Key data from the survey, such as household wealth status, and community members' perception of their health and health concerns, will contextualize the findings from the other three RFI studies, and provide insights into potential health disparities and inequalities. By integrating disease prevalence with fever etiology and causes of death, a multi-layered understanding of health dynamics in the study area could be attained. Additionally, knowledge from the survey will play a vital role for estimating incidence. This involves assimilating data on proportion of the population with fever who visited various healthcare providers from the survey, with the number of fever patients recruited from the village health workers or health facilities, captured from the fever etiology studies.

The study will also bridge the gap in community-based seroprevalence estimates for common pathogens causing fever, minimizing biases related to health-seeking behavior in facility-based studies. The data can also to be used for population-based incidence estimates (41,42). Furthermore, the updated prevalences of hepatitis B and C can be instrumental in evaluating the coverage and effectiveness of HBV vaccination programs, and inform HCV screening and treatment strategies in the region in the era of direct-acting antiviral agents (43,44). This study also extends its scope to gather insights into the health perspectives and concerns of community members, facilitating the alignment of future interventions with the needs and expectations of the population. Specifically, for self-perceived status, while existing studies primarily focus on the elderly population, this study encompasses a broader age range(45–48). This can inform tailored interventions to improve the quality of life for a more extensive population (49–51).

This study encourages regional collaboration by applying consistent research methods and seeking representative samples across study sites, allowing effective cross-country comparisons and fostering collaborative approaches to address shared health challenges (3).

The findings of this study are limited in their generalizability and should be interpreted with caution when considering broader implications for rural populations in the study countries. However, the study was specifically designed for and conducted within the selected rural areas of Bangladesh, Cambodia, and Thailand, where SEACTN is operational. This focused approach allows for insights into health challenges and opportunities within these areas. The cross-sectional design and observational nature of the study is at risk of, such as temporal bias, selection bias, and participation bias. However, to minimize the bias as much as possible, we designed the survey with appropriate evaluation methods, and adequate training and supporting material will be well prepared for the field work.

This planned household prevalence survey will significantly contribute to disease prevention and control in the region by offering a comprehensive understanding of health conditions of the rural populations. Complementing the results from other RFI initiatives, it will serve as a foundation for evidence-based interventions, shaping future research and disease control priorities. The study findings will not only serve the next phase of SEACTN but also extend to the broader context, ultimately leading to improved health outcomes in rural communities.

Ethics and dissemination

This study protocol was approved by the Oxford Tropical Research Ethics Committee (OxTREC Ref: 6-22), National Research Ethics Committee of Bangladesh Medical Research Council (BMRC/NREC/2022-2025/69), the Cambodian National Ethics Committee for Health Research (23rd December 2022 NECHR), the Chiang Rai Provincial Public Health Research Ethical Committee (CRPPHO 75/2565).

Safety considerations

Participants may experience slight discomfort during the blood draw, however, our experienced clinical staff performing the phlebotomy will take special attention to minimize this discomfort. While some interview questions may be personal, all data will be handled confidentially, and linked only to study IDs. Participants will also be informed of their right to skip questions or end the interview if they feel uncomfortable.

Data deposition and curation

The database and all electronic data will be stored on secure servers that are backed up daily, with weekly off-site storage. Paper records, if they exist, will be kept in secure storage such as locked cabinets; if necessary, the record will be scanned and stored electronically. Study data will be archived in accordance with Mahidol Oxford Tropical Medicine Research Unit (MORU) SOPs. Electronic data will be de-identified and preserved indefinitely. Paper records will be preserved for five years after study completion. With participant's consent, electronic data can be shared according to the terms defined in the MORU data sharing policy with other researchers for future use (52).

For peer review only

Dissemination

The results of the study will be summarized in plain language in both English and the official local language. These summaries will be disseminated via the local health authorities and partners. The findings will also be disseminated internationally through conference presentations and peer-reviewed academic journal publications, aligning with Wellcome Trust policy and guidelines.

References

1. Life expectancy at birth (years) [Internet]. [cited 2023 Aug 23]. Available from: https://www.who.int/data/gho/data/indicators/indicator-details/GHO/life-expectancy-at-birth-(years)

2. Bygbjerg IC. Double Burden of Noncommunicable and Infectious Diseases in Developing Countries. Science. 2012 Sep 21;337(6101):1499–501.

3. Chongsuvivatwong V, Phua KH, Yap MT, Pocock NS, Hashim JH, Chhem R, et al. Health and health-care systems in southeast Asia: diversity and transitions. The Lancet. 2011 Jan 29;377(9763):429–37.

4. Coker RJ, Hunter BM, Rudge JW, Liverani M, Hanvoravongchai P. Emerging infectious diseases in southeast Asia: regional challenges to control. The Lancet. 2011 Feb 12;377(9765):599–609.

5. Dans A, Ng N, Varghese C, Tai ES, Firestone R, Bonita R. The rise of chronic non-communicable diseases in southeast Asia: time for action. The Lancet. 2011 Feb 19;377(9766):680–9.

6. Murray CJL. The Global Burden of Disease Study at 30 years. Nat Med. 2022 Oct;28(10):2019–26.

7. Voigt K, King NB. Out of Alignment? Limitations of the Global Burden of Disease in Assessing the Allocation of Global Health Aid. Public Health Ethics. 2017 Nov;10(3):244–56.

8. Global Burden of Disease Study 2019 (GBD 2019) Data Input Sources Tool | GHDx [Internet]. [cited 2023 Aug 23]. Available from: https://ghdx.healthdata.org/gbd-2019/data-input-sources

9. World Health Organization. Regional Office for the Western Pacific. UHC and SDG country profiles : Cambodia [Internet]. Manila: WHO Regional Office for the Western Pacific; 2018 [cited 2021 Oct 6]. Available from: https://apps.who.int/iris/handle/10665/272307

10. UHC and SDG country profiles : Lao People's Democratic Republic [Internet]. [cited 2023 Jan 20]. Available from: https://apps.who.int/iris/handle/10665/272312

11. World Health Organization. Regional Office for South-East Asia. 2019 Health SDG profile: Thailand [Internet]. World Health Organization. Regional Office for South-East Asia; 2019 [cited 2021 Oct 6]. Available from: https://apps.who.int/iris/handle/10665/327767

For peer review only

12. World Health Organization. Regional Office for South-East Asia. 2019 Health SDG profile: Bangladesh [Internet]. World Health Organization. Regional Office for South-East Asia; 2019 [cited 2021 Oct 6]. Available from: https://apps.who.int/iris/handle/10665/327747

13. World Health Organization. Regional Office for South-East Asia. 2019 Health SDG profile: Myanmar [Internet]. World Health Organization. Regional Office for South-East Asia; 2019 [cited 2021 Oct 6]. Available from: https://apps.who.int/iris/handle/10665/327762

14. Chandna A, Chew R, Shwe Nwe Htun N, Peto TJ, Zhang M, Liverani M, et al. Defining the burden of febrile illness in rural South and Southeast Asia: an open letter to announce the launch of the Rural Febrile Illness project. Wellcome Open Res. 2022 Mar 10;6:64.

15. Department of Economic and Social Affairs, United Nation. Designing Household Survey Samples: Practical Guidelines [Internet]. United Nation Publication; 2008. Available from: chrome-extension://efaidnbmnnnibpcajpcglclefindmkaj/https://unstats.un.org/unsd/publication/seriesf/seriesf_98e.pdf

16. The World Health Organization. The WHO STEPwise approach to noncommunicable disease risk factor surveillance [Internet]. 2020 [cited 2023 May 25]. Available from: https://www.who.int/teams/noncommunicable-diseases/surveillance/systems-tools/steps/manuals

17. Milligan P, Njie A, Bennett S. Comparison of two cluster sampling methods for health surveys in developing countries. Int J Epidemiol. 2004 Jun 1;33(3):469–76.

18. ICF. Demographic and Health Survey Interviewer's Manual [Internet]. Rockville, Maryland, USA: ICF; 2020 [cited 2023 Sep 22]. Available from: https://dhsprogram.com/publications/publication-dhsm1-dhs-questionnaires-and-manuals.cfm

19. MoHS/Myanmar M of H and S, ICF. Myanmar Demographic and Health Survey 2015-16 [Internet]. Nay Pyi Taw, Myanmar, and Rockville, Maryland USA: Ministry of Health and Sports and ICF; 2017 Mar [cited 2021 Oct 28]. Available from: https://dhsprogram.com/publications/publication-fr324-dhs-final-reports.cfm

20. Niport NI of PR and T, ICF. Bangladesh Demographic and Health Survey 2017-18 [Internet]. Dhakar, Bangladesh, and Rockville, Maryland, USA: NIPORT and ICF; 2020 Oct [cited 2021 Oct 28]. Available from: https://dhsprogram.com/publications/publication-FR344-DHS-Final-Reports.cfm

21. Statistics/Cambodia NI of, Health/Cambodia DG for, International ICF. Cambodia Demographic and Health Survey 2014. 2015 Sep 1 [cited 2021 Oct 28]; Available from: https://dhsprogram.com/publications/publication-fr312-dhs-final-reports.cfm

22. National Institute of Preventive and Social medicine (NIPSOM). National STEPS survey for non-communicable diseases risk factors in Bangladesh 2018 [Internet]. Dhakar, Bangladesh: National Institute of Preventive and Social medicine (NIPSOM); 2018 [cited 2021 Oct 28]. Available from: https://apps.who.int/iris/handle/10665/332886

23. University of Health Science, Preventive Medicine Department of the Ministry of Health. Prevalence of non-communicable disease risk factors in Cambodia, STEPS Survey Country report 2010 [Internet]. Phnom Penh, Cambodia: University of Health Science, Preventive Medicine Department of

For peer review only

the Ministry of Health; 2010 [cited 2021 Oct 28]. Available from: https://extranet.who.int/ncdsmicrodata/index.php/catalog/378

24. Lao People's Democratic Republic - STEPS 2013 [Internet]. [cited 2021 Oct 28]. Available from: https://extranet.who.int/ncdsmicrodata/index.php/catalog/588

25. National Statistical Office of Thailand. Thailand Multiple Indicator Cluster Survey 2019, Survey finding report [Internet]. Bangkok, Thailand: National Statistical Office of Thailand; 2020 [cited 2021 Oct 28]. Available from: https://www.unicef.org/thailand/reports/thailand-multiple-indicator-cluster-survey-2019

26. Lynch E, Falq G, Sun C, Bunchhoeung PDT, Huerga H, Loarec A, et al. Hepatitis C viraemic and seroprevalence and risk factors for positivity in Northwest Cambodia: a household cross-sectional serosurvey. BMC Infect Dis. 2021 Feb 26;21(1):223.

27. Komada K, Sugiyama M, Vongphrachanh P, Xeuatvongsa A, Khamphaphongphane B, Kitamura T, et al. Seroprevalence of chronic hepatitis B, as determined from dried blood spots, among children and their mothers in central Lao People's Democratic Republic: a multistage, stratified cluster sampling survey. Int J Infect Dis IJID Off Publ Int Soc Infect Dis. 2015 Jul;36:21–6.

28. Ashraf H, Alam NH, Rothermundt C, Brooks A, Bardhan P, Hossain L, et al. Prevalence and risk factors of hepatitis B and C virus infections in an impoverished urban community in Dhaka, Bangladesh. BMC Infect Dis. 2010 Jul 15;10:208.

29. Aye Aye Lwin, Khin Saw Aye, Moh Moh Htun, Yi Yi Kyaw, Ko Ko Zaw, Toe Thiri Aung, et al. Sero-prevalence of Hepatitis B and C Viral Infections in Myanmar: National and Regional Survey in 2015. Myanmar Health Sci Res J. 2017;29(3):167–75.

30. The EquityTool [Internet]. Equity Tool. [cited 2021 Oct 28]. Available from: https://www.equitytool.org/the-equity-tool-2/

31. Chakraborty NM, Fry K, Behl R, Longfield K. Simplified Asset Indices to Measure Wealth and Equity in Health Programs: A Reliability and Validity Analysis Using Survey Data From 16 Countries. Glob Health Sci Pract. 2016 Mar 21;4(1):141–54.

32. World Health Survey (WHS) [Internet]. [cited 2022 Apr 20]. Available from: https://apps.who.int/healthinfo/systems/surveydata/index.php/catalog/whs/about

33. van der Zouwen J, Smit JH. Evaluating Survey Questions by Analyzing Patterns of Behavior Codes and Question–Answer Sequences: A Diagnostic Approach. In: Methods for Testing and Evaluating Survey Questionnaires [Internet]. John Wiley & Sons, Ltd; 2004 [cited 2023 Sep 22]. p. 109–30. Available from: https://onlinelibrary.wiley.com/doi/abs/10.1002/0471654728.ch6

34. Presser S, Couper MP, Lessler JT, Martin E, Martin J, Rothgeb JM, et al. Methods for Testing and Evaluating Survey Questions. Public Opin Q. 2004 Mar 1;68(1):109–30.

35. Alemayehu S, Feghali KC, Cowden J, Komisar J, Ockenhouse CF, Kamau E. Comparative evaluation of published real-time PCR assays for the detection of malaria following MIQE guidelines. Malar J. 2013 Aug 8;12:277.

For peer review only

36. Wampfler R, Mwingira F, Javati S, Robinson L, Betuela I, Siba P, et al. Strategies for Detection of Plasmodium species Gametocytes. PLOS ONE. 2013 Sep 27;8(9):e76316.

37. Rosanas-Urgell A, Mueller D, Betuela I, Barnadas C, Iga J, Zimmerman PA, et al. Comparison of diagnostic methods for the detection and quantification of the four sympatric Plasmodium species in field samples from Papua New Guinea. Malar J. 2010 Dec 14;9:361.

38. Hofmann N, Mwingira F, Shekalaghe S, Robinson LJ, Mueller I, Felger I. Ultra-Sensitive Detection of Plasmodium falciparum by Amplification of Multi-Copy Subtelomeric Targets. PLOS Med. 2015 Mar 3;12(3):e1001788.

39. Case Report: Case Series of Human Plasmodium knowlesi Infection on the Southern Border of Thailand - PMC [Internet]. [cited 2024 Feb 20]. Available from: https://www.ncbi.nlm.nih.gov/pmc/articles/PMC6896887/

40. Hartung C, Lerer A, Anokwa Y, Tseng C, Brunette W, Borriello G. Open data kit: tools to build information services for developing regions. In: Proceedings of the 4th ACM/IEEE International Conference on Information and Communication Technologies and Development [Internet]. New York, NY, USA: Association for Computing Machinery; 2010 [cited 2023 Aug 23]. p. 1–12. (ICTD ’10). Available from: https://doi.org/10.1145/2369220.2369236

41. Andrews JR, Barkume C, Yu AT, Saha SK, Qamar FN, Garrett D, et al. Integrating Facility-Based Surveillance With Healthcare Utilization Surveys to Estimate Enteric Fever Incidence: Methods and Challenges. J Infect Dis. 2018 Nov 10;218(suppl_4):S268–76.

42. Aiemjoy K, Seidman JC, Saha S, Munira SJ, Sajib MSI, Sium SMA, et al. Estimating typhoid incidence from community-based serosurveys: a multicohort study. Lancet Microbe. 2022 Aug 1;3(8):e578–87.

43. Falade-Nwulia O, Suarez-Cuervo C, Nelson DR, Fried MW, Segal JB, Sulkowski MS. Oral Direct-Acting Agent Therapy for Hepatitis C Virus Infection. Ann Intern Med. 2017 May 2;166(9):637–48.

44. Hepatitis B (HepB3) immunization coverage among 1-year-olds (%) [Internet]. [cited 2023 Aug 3]. Available from: https://www.who.int/data/gho/data/indicators/indicator-details/GHO/hepatitis-b-(hepb3)-immunization-coverage-among-1-year-olds-(-)

45. Thet Ssoe H. Myanmar Aging Survey. In: Gu D, Dupre ME, editors. Encyclopedia of Gerontology and Population Aging [Internet]. Cham: Springer International Publishing; 2021 [cited 2023 Aug 23]. p. 3375–8. Available from: https://doi.org/10.1007/978-3-030-22009-9_973

46. “The situation of Thailand’s older population: An update based on the 2” by John KNODEL, Bussarawan TEERAWICHITCHAINAN et al. [Internet]. [cited 2023 Aug 23]. Available from: https://ink.library.smu.edu.sg/soss_research/1948/

47. Factors related to quality of life among older adults in Bangladesh: A cross sectional survey - Uddin - 2017 - Nursing & Health Sciences - Wiley Online Library [Internet]. [cited 2023 Aug 23]. Available from: https://onlinelibrary.wiley.com/doi/full/10.1111/nhs.12385

For peer review only

48. Jankhotkaew J, Chaiyasong S, Waleewong O, Siengsounthone L, Sengngam K, Douangvichit D, et al. The impact of heavy drinkers on others' health and well-being in Lao PDR and Thailand. J Subst Use. 2017;22(6):617–23.

49. DeSalvo KB, Fan VS, McDonell MB, Fihn SD. Predicting Mortality and Healthcare Utilization with a Single Question. Health Serv Res. 2005 Aug;40(4):1234–46.

50. Benyamini Y, Idler EL. Community Studies Reporting Association between Self-Rated Health and Mortality: Additional Studies, 1995 to 1998. Res Aging. 1999 May 1;21(3):392–401.

51. Makovski TT, Schmitz S, Zeegers MP, Stranges S, van den Akker M. Multimorbidity and quality of life: Systematic literature review and meta-analysis. Ageing Res Rev. 2019 Aug;53:100903.

52. MORU Tropical Network Policy on Sharing Data and Other Outputs [Internet]. [cited 2023 Oct 17]. Available from: https://www.tropmedres.ac/units/moru-bangkok/bioethics-engagement/data-sharing/moru-tropical-network-policy-on-sharing-data-and-other-outputs

For peer review only

Declarations

Authors' contribution

MZ, NSNH, TJP contributed to conception and design of the study, and drafted the manuscript.

SI, AS, MAI, AKN, RT, LD, CP, RC, EMB, WT, JW, SDB, SJL contributed to the design of the study, and provided critical review of the manuscript.

NW, CM contributed to the design of the study, data collection platform development, and provided critical review of the manuscript.

SIZ contributed to the design of the study, provided the study sites' map and critical review of the manuscript.

ML, RJM, NPJD, YL contributed to the conception of the study, and provided critical review of the manuscript.

Funding statement

This research was funded in whole, or in part, by the Wellcome Trust [215604/Z/19/Z]. For the purpose of Open Access, the author has applied a CC BY public copyright license to any Author Accepted Manuscript version arising from this submission.

Competing interests

Nothing to declare

Acknowledgements

The authors are grateful for the support from the local authorities in the participating countries. We acknowledge all staff from our partners and collaborators for their devoted contributions to the survey.

Tables and figures

Table 1. Required sample size tested against the estimated prevalence of key indicators of the highest prevalence, overall and by age groups of interests.

	**Expected number of participants**	**Indicator (estimated prevalence)**	**Required sample size ***
**Overall**	1500	Overweight (20%)	369
**Age groups (years)**			
≥15	1050	Current drinker (55%)	570
<5	150	Anemia (30%)	122
15-49	750	Injury or death caused by recent accident (20%)	414

For peer review only

≥50	300	Hypertension (20%)	257

*The formula used for calculating sample size is shown below. Assessing the sufficiency of the sample size for the key indicators of the highest prevalences ensures sufficient statistical power for indicators with lower prevalence. A degree of precision of 5% is applied for the sample size calculation overall and across age groups, except for the under 5 years and the 50 years or older groups, for which degrees of precision of 10% and 6% are applied, respectively.

$$n = DEFF \times \frac{Np(1-p)}{\frac{d^2}{1.96^2}(N-1) + p(1-p)}$$

n: Sample size.

DEFF: Design effect. Design effect of 1.5 is used to account for a slightly expected increase in variance due to clustering.

N: population size

p: expected prevalence of key indicators.

d: degree of precision.

Figure 1. The study sites and population of the Cross-sectional Household Health Survey conducted by the South and Southeast Asia Community-based Trials Network (SEACTN).

The Bangladesh site includes 130 villages from Cox's Bazar and Bandarban districts; the Cambodia site includes 82 villages from Battambang and Pailin provinces; and the Thailand site includes 179 villages located in Chiang Rai province.

HHS: The Household Health Survey; SEACTN: the South and Southeast Asia Community-based Trials Network

Figure 2. Questionnaire interviews, physical examinations, and laboratory procedures administrated to study participants according to age and sex.

EDTA: Blood sample collection tubes containing ethylenediaminetetraacetic acid.

For peer review only

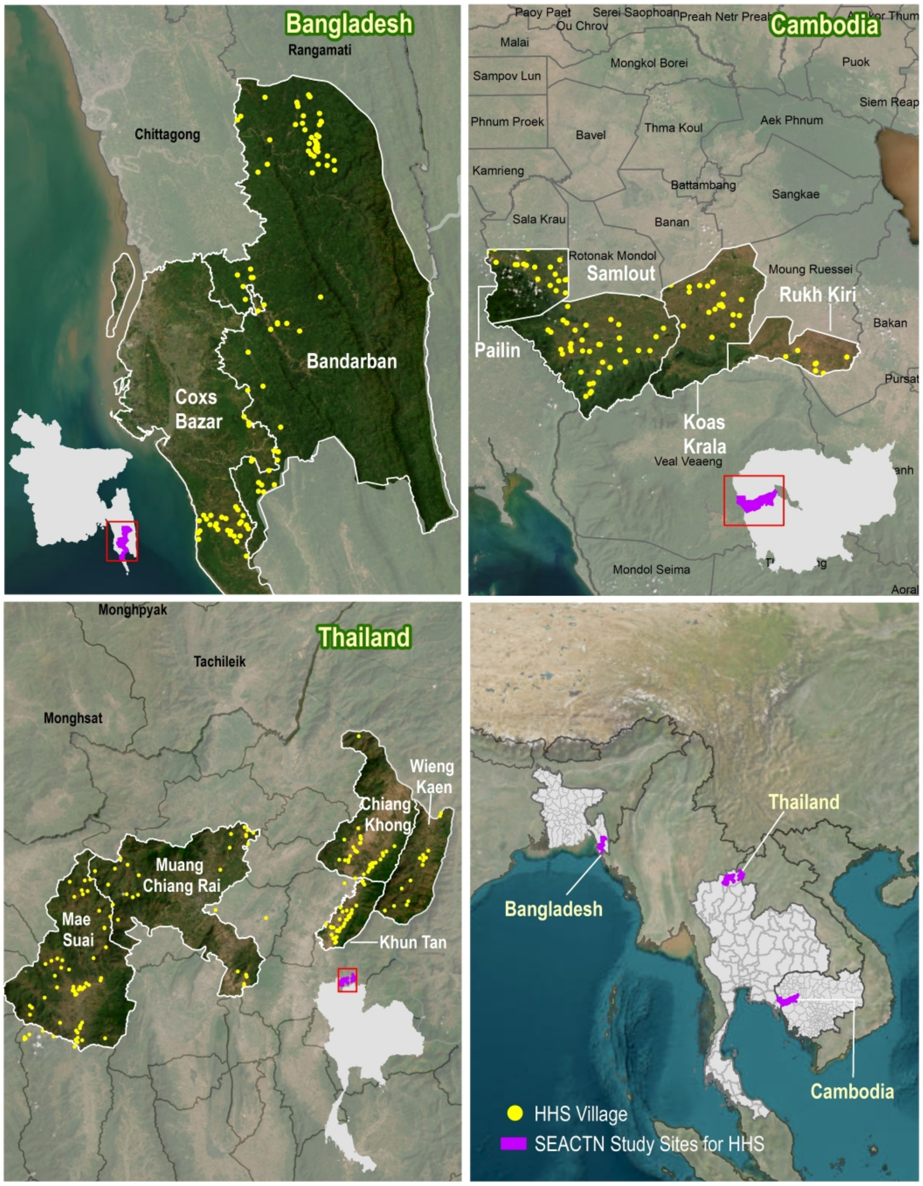

Figure 1. The study sites and population of the Cross-sectional Household Health Survey conducted by the South and Southeast Asia Community-based Trials Network (SEACTN).
The Bangladesh site includes 130 villages from Cox's Bazar and Bandarban districts; the Cambodia site includes 82 villages from Battambang and Pailin provinces; and the Thailand site includes 179 villages located in Chiang Rai province.
HHS: The Household Health Survey; SEACTN: the South and Southeast Asia Community-based Trials Network

218x279mm (500 x 500 DPI)

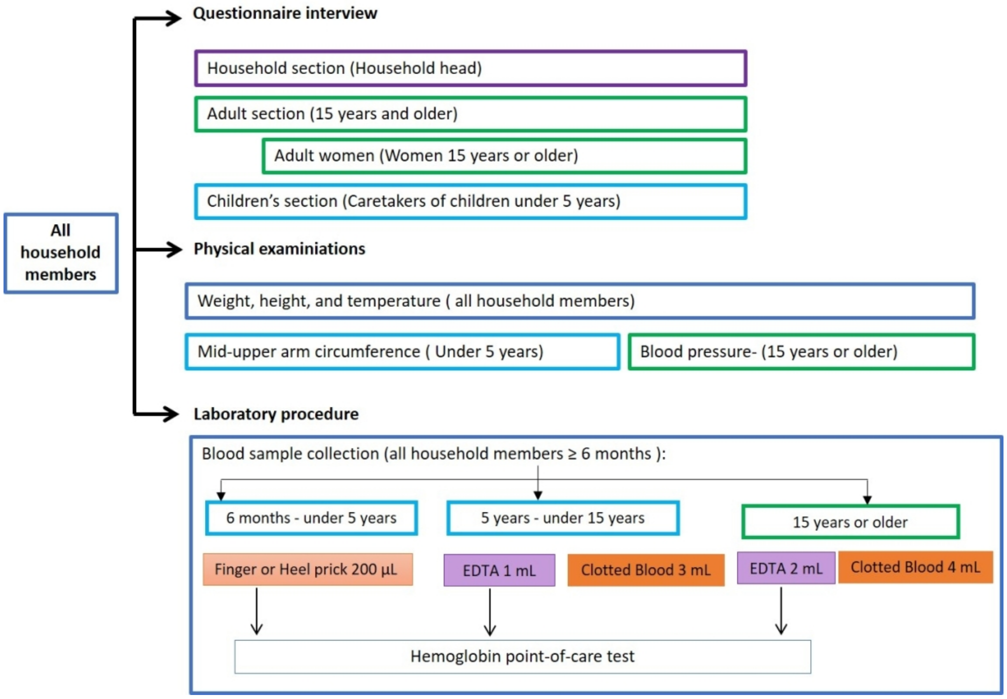

Figure 2. Questionnaire interviews, physical examinations, and laboratory procedures administrated to study participants according to age and sex.
EDTA: Blood sample collection tubes containing ethylenediaminetetraacetic acid.

230x161mm (150 x 150 DPI)

Appendix A. Questionnaire

DEFINING THE HIDDEN BURDEN OF DISEASE IN RURAL COMMUNITIES IN BANGLADESH, CAMBODIA AND THAILAND: A CROSS-SECTIONAL HOUSEHOLD HEALTH SURVEY PROTOCOL

Study site:

Village:

Household ID:

	Household head section		
Code	Question	Answer	Skip logic
S1a	(Household head or adult member of the household) Please give me the names of the persons who usually live in your household starting from the household head.<br><br>*Prompt: Have you told me the names of all the household members, even the ones are not here now?*<br><br>*Record the full name with the study ID in the paper-based household checklist*	Initials, and assign study ID automatically	
S1b	Could you now give me the names of the guests of the household who stayed here last night?		

For peer review only - http://bmjopen.bmj.com/site/about/guidelines.xhtml

For peer review only

S2	What is the relationship of [NAME] to the head of the household?	Head→S3<br>Wife or husband→S4<br>Son or daughter→S4<br>Son-in-law or daughter-in-law→S4<br>Grandchild→S4<br>Parent→S4<br>Parent-in-law→S4<br>Brother or sister→S4<br>Other relative→S4<br>Adopted/ Foster/ Stepchild →S4<br>Not related→S4<br>Don't know→S4	If S2=Head then S3, else skip to S4
S3	What is your ethnic group?	Ethnic group1<br>Ethnic group 2<br>[insert names of locally relevant ethnic groups]	
S4	Is [NAME] male or female?	Male<br>Female	
S5	Does [NAME] usually live here in the past month?	Yes<br>No	
S6	Did [NAME] stay here last night?	Yes<br>No	
S7	How old is [NAME]?	_______ in year<br><br>If age 5-14 years→S8-S10<br><br>Else→S1, until completing the information of all the mentioned household members→ S11	Go back to S1 until completing the information of household members.

For peer review only - http://bmjopen.bmj.com/site/about/guidelines.xhtml

S8	Did [NAME] attend school or any early childhood education program at any time during the [2021-2022] school year?	Yes<br>No→S10	If S8=No, then S10
S9	During [this/that] school year, what level [is/was] (NAME) attending?	Early childhood<br>Primary<br>Secondary<br>Higher<br>Don't know<br><br>→ S1, until the information of all household members is taken →S11	
S10	What Is the highest level of school [NAME] has attended?	Early childhood<br>Primary<br>Secondary<br>Higher<br>Don't know<br><br>→ S1, until the information of all household members is taken→ S11	Go back to S1 until completing the list

	**Household Schedule summary**		
Code	Question	Answers	Skip logic
S11	Total persons in household	Auto calculation	
S12	Number of household member >=18 years	Auto calculation	
S13	Number of household member 15-17 years	Auto calculation	
S14	Number of women household member>=15 years	Auto calculation	

For peer review only - http://bmjopen.bmj.com/site/about/guidelines.xhtml

S15	Number of school age children 5-14 years old	Auto calculation	
S16	Number of children under 5 years old	Auto calculation	
S17	Just to make sure that I have a complete listing: are there any other people such as small children or infants that we have not listed?	Yes →S1<br>No	If yes, go to S1
S18	Are there any other people who may not be members of your family, such as domestic servants, lodgers, or friends who usually live here?	Yes→S1<br>No	If yes, go to S1
S19	Are there any guests or temporary visitors staying here, or anyone else who stayed here last night, who have not been listed?	Yes→S1<br>No	If yes, go to S1
S20	Is there anybody, who is a household member or a guest who stayed in your household last night, is not here for the survey now?	Yes →S1<br>No →EQ1	
S21	Can you tell me the names of the people who are not here?	Choose from the Initials and study IDs	
S22	What are the reasons [name] is not here now?	Not available at the time (work or school)<br>Physical illness or disability<br>Mental illness or disability<br>Other reason, specify:_____<br>Don't know	

	**Household section**		
Code	Question	Answers	Skip logic
	Now I'm going to ask you some questions about your household amenities and possessions.<br>[use Equity Tool of the country. Example Cambodia]		
	Does your household have		
EQ1	… Electricity	Yes<br>No	
EQ2	… a television?	Yes<br>No	
EQ3	… refrigerator?	Yes	

For peer review only - http://bmjopen.bmj.com/site/about/guidelines.xhtml

		No	
EQ4	…. CD or DVD player	Yes<br>No	
EQ5	… wardrobe?	Yes<br>No	
EQ6	…Generator or Battery or Solar panel	Yes<br>No	
EQ7	Does any member of your household own a motorcycle or scooter?	Yes<br>No	
EQ8	… A watch?	Yes<br>No	
EQ9	Does any member of this household have a bank account?	Yes<br>No	
EQ10	What is the main material of the floor?	Ceramic tiles<br>Wood planks<br>Other material	
EQ11	What is the main material of the exterior walls?	Cement blocks<br>Palm / bamboo / thatch<br>Other material	
	**Housing**		
HH1	What is the main source of drinking water for members of your household?<br><br>*(show card)*<br>*The showcard provided the definitions of the water source categories. Based on the description of the respondent, choose from the categories.*	<u>Piped water</u><br>Piped into dwelling<br>Piped to yard/plot<br>Piped to neighbour<br>Public tap/standpipe<br>Tube well or borehole<br><u>Dug well</u><br>Protected well<br>Unprotected well<br><u>Water from spring</u><br>Protected spring<br>Unprotected spring	If "bottled water", then HH4

For peer review only - http://bmjopen.bmj.com/site/about/guidelines.xhtml

		Rainwater<br>Tanker truck<br>Cart with small tank<br>Surface water (river/dam/Lake/pond/stream/canal/Irrigation channel)<br>Bottled water→HH4<br><br>Other, specify:_____	
HH2	Do you do anything to the water to make it safer to drink?<br><br>*Prompt: Participants might not know it's for making the water safer to drink. The interviewer can prompt if they do anything to the water before drinking.*	Yes<br>No → HH4<br>Don't know	
HH3	What do you usually do to make the water safer to drink? Anything else? (check all that apply)	Boil<br>Add bleach/chlorine<br>Strain through a cloth<br>Use water filter (ceramic/sand/composite/etc)<br>Solar disinfection<br>Let it stand and settle<br>Other, specify______<br>Don't know	
HH4	What kind of toilet facility do members of your household usually use?<br><br>*If the respondent answers in general terms such as "flush toilet," probe to determine where the toilet flushes to; likewise, if the respondent answers "latrine," probe to determine the type of latrine.*	<u>Flush or pour flush toilet</u><br>Flush to piped sewer system<br>Flush to septic tank<br>Flush to pit latrine<br>Flush to somewhere else<br>Flush, don't know where<br><br><u>Pit latrine</u><br>Ventilated improved pit latrine	

For peer review only - http://bmjopen.bmj.com/site/about/guidelines.xhtml

	(show card)<br>*The show card provides you definitions of types of toilet, according to the description from the respondent choose the appropriate option.*<br>*If you cannot choose according to the participants description, show them the pictures on the showcard.*	Pit latrine with slab<br>Pit latrine without slab/open pit<br><br>Composting toilet<br>Bucket toilet<br>Hanging toilet/hanging latrine<br>No facility/bush/field<br><br>Other, specify _____________	
HH5	What type of fuel does your household mainly use for cooking?	Electricity<br>Liquefied Petroleum Gas (LPG)<br>Charcoal<br>Wood<br>No food cooked in household<br>Other (specify) ____________________	
HH6	Does the stove have a chimney or fan?	Yes<br>No<br>Don't know	
HH7	Is the cooking usually done in the house, in a separate building, or outdoors?	In the house<br>In a separate building<br>Outdoors<br>Other, specify:______	
HH8	Does your household have any mosquito nets?	Yes<br>No→ HH10	If HH8=No, then HH10
HH9	How many mosquito nets does your household have?	Number of net_______	
HH10	Do all of your household windows have mosquito/fly screens?	Yes<br>No	

For peer review only - http://bmjopen.bmj.com/site/about/guidelines.xhtml

ST1	How often is your house floor being cleaned (e.g. swept)?	Everyday (7 days/week)<br>Almost everyday (5-6 days/week)<br>Every other day (3-4 days/week)<br>Weekly (1-2 days/week)<br>Monthly (1-3 days/month)<br>Scarcely (a few times/ year)<br>Never	
ST2	How often do you or other members of your household perform activities to maintain grass or weed in or around your household, or work on a vegetable garden?<br><br>Prompt: For example, cut or trim weeds, plant or pick vegetables in the yard, garden, by the walls or fences of your household?	Everyday (7 days/week)<br>Almost everyday (5-6 days/week)<br>Every other day (3-4 days/week)<br>Weekly (1-2 days/week)<br>Monthly (1-3 days/month)<br>Scarcely (a few times/ year)<br>Never	
	**Household member check**		
	Now I'm going to asking you some questions about the members of your household including you, if anyone have had an accident, or had illness in the past 30 days.		
	**Accident and illness in the past 30 days**		
HM1	Was any person of your household injured or killed in an accident?<br>Prompt: anyone injured or killed in an accident any time before the interview.	Yes, how many? ___<br>No→HM14	If HM1=No, then HM14
HM2	What the name of the person got injured or killed in an accident?	Identify the ID code and show initial<br>Not in the list, initial:____	
HM3	Could you tell me in what type of accident (NAME) was injured or killed?	Landmine/unexploded bomb (UXO)<br>Gunshot/weapon<br>Road accident<br>Fire/burning<br>Snake/animal bite<br>Fall from tree/building<br>Drowning	

For peer review only - http://bmjopen.bmj.com/site/about/guidelines.xhtml

		Poisoning (chemical)<br>Violence/assault<br>Other, specify________<br>Don't know	
HM4	Is (NAME) still alive?	Yes<br>No→HM6	If HM4 =No, then HM6
HM5	In your opinion, was (NAME'S) injury serious, moderate, or slight?	Serious<br>Moderate<br>Slight<br>DON'T KNOW	
HM6	Was (NAME's) death due to accident?	Yes ⎤→ HM7<br>No ⎦	→HM7
HM7	Did the accident happen in the past 30 days?	Yes<br>No→HM14	If No then HM 14
HM8	Was advice or treatment sought for [Name] after the accident?	Yes<br>No→ HM14	If No then HM14
HM9	Where was advice or treatment first sought for [NAME] after the accident?	Public sector<br>Primary care unit/Primary care centers/Community Health centers<br>Community hospital<br>General hospital/Central hospital<br>University hospital<br>Government hospital<br>Private medical sector<br>Poly private clinic/private clinic<br>Private hospital<br>Others<br>source<br>Traditional/herbal medicine<br>Traditional healer<br>Self-bought medicine<br>Relatives/ Friends<br>, specify: ________	

For peer review only - http://bmjopen.bmj.com/site/about/guidelines.xhtml

HM10	Where did the money come from to pay for transportation and treatment for the members of your household who had an injury over the past 30 days?<br><br>Record all that mentioned<br><br>*The payment method for transportation and medical care can be different. If the respond provided one method, the interviewer should probe and confirm if both payment for transportation and treatment are from the same method.*<br><br>*If any out-of-pocket payment method is selected, then proceed to HM11.*	Subsidy or exemption→ HM14<br>Insurance scheme →HM14<br>Universal coverage card (UC)<br>Social Security/ worker compensated fund (SSSS)<br>civil servant medical benefit scheme (CSMBS)<br>State enterprise<br>Independent agency of the state<br>Local government<br>Private health insurance<br>Health insurance covered by employer<br>Other health insurance, specify____________<br><br>Out of pocket → HM11<br>Wage/income<br>Loan/borrowed money<br>Sale of assets<br>Gift from relatives/neighbours/friends<br>Saving/ family funds<br><br>Other, specify________→HM14<br>Don't know→HM14	
HM11	How much in total was spent on [NAME]'s treatment at (name of the place)?	________amount of money<br>Don't know	If HM10 checked out of pocket payment.
HM12	How much in total was spent on transport to go to and return from (name of the place)?	_____amount of money<br>Don't know	If HM10 checked out of pocket payment.
HM13	Has the debt been repaid?<br>*(If HM10= Loan/borrowed money)*	Yes<br>No<br>Don't know	If HM10= Loan/borrowed money
HM14	Please tell me if any member of your household (including you) is sick, has an illness now or at any time in the last 30 days?	Yes, how many? _________<br>No → End of the household head section	If HM14=No, then end of the section

For peer review only - http://bmjopen.bmj.com/site/about/guidelines.xhtml

	It can be **acute** disease or previous diagnosed **chronic** disease.<br>An acute illness is a condition that appears suddenly: the person did not have it immediately before becoming ill.<br>A chronic disease is an illness that will not go away or takes a long time to go away, even when treated.<br><br>Prompt if nobody:<br>Is there anyone had fever?<br>Any infant or child in your family was ill?		
HM15	Now I would like to ask you some questions about each person who is sick now or at any time in the <u>last 30 days</u>.<br><br>Could you tell me his/her/their name(s)? Then we will talk about one person at a time.	Identify all that from the list of the family member (ID number)<br><br>No body→ end of the household head section	
HM16	What type of health problems or symptoms did [name] have or is having during this illness?<br><br>DO NOT READ. TICK ONE BOX FOR EACH GROUP OF SYMPTOMS MENTIONED.<br><br><br>*If the respondent provide a diagnosis directly, especially for an acute condition, please still probe for the symptoms. Check all the symptoms that apply, and check "other acute condition, specify: ____ and write down the diagnosis.*<br>*For example: if the respondent answer " Covid", check "other acute condition, specify: COVID". Then continue probe what did the symptoms the person have, and check all the symptoms that are described.*	<u>Acute conditions</u><br>Fever<br>Persistent headaches<br>Persistent cough<br>Running nose<br>Difficulty breathing, fast breathing<br>Difficulty swallowing/ throat pain<br>Difficulty seeing or other eye complaint<br>Stomach pain, nausea, vomiting, could not eat<br>Thirst/ sweating<br>Watery diarrhoea<br>Bloody stools<br>Skin disease, such as rush, irritation, open sores<br>gyn-obstetrics<br>Could not sleep<br>Feeling tired<br>Constipation<br>Convulsion/ seizure	

For peer review only - http://bmjopen.bmj.com/site/about/guidelines.xhtml

		Musculoskeletal pain (general body pain, back and joint pain)<br><br><u>Chronic disease</u><br>Hypertension, high blood pressure<br>Heart disease, heart attack consequence<br>Diabetes, high blood sugar<br>Asthma, wheezing, chronic difficulty breathing<br>HIV infection, AIDS<br>Arthritis, chronic body pain<br>High cholesterol<br>Ulcer, chronic stomach pain<br>Stroke consequence<br>Epilepsy, seizures, fits<br>Cancer<br>Tuberculosis<br>Liver disease<br>Depression<br><br>Other acute illness, specify: _______<br>Other chronic illness, specify:________<br>Don't know	
HM17	In your opinion, was [NAME]'s illness serious, moderate, or slight?	Serious<br>Moderate<br>Slight<br>Don't know	
HM18	Was advice or treatment sought for [Name]'s illness?	Yes<br>No→End of the household head section	If HM18=No, then end of the section
HM19	Where was advice or treatment first sought for [NAME]'s illness?	<u>Public sector</u><br>Government hospital<br>Government health centre<br>Government health post	

For peer review only - http://bmjopen.bmj.com/site/about/guidelines.xhtml

	[insert Country specific list]	Mobile clinic<br>Community health worker/field worker<br>Other public sector<br>Private medical sector<br>Private hospital<br>Private clinic<br>Pharmacy<br>Private doctor<br>Mobile clinic<br>Community health worker/field worker<br>Other private medical sector<br>NGO medical sector<br>NGO hospital<br>NGO clinic<br>Other NGO medical<br><br>Other source<br>Shop<br>Traditional practitioner<br>Market<br>Itinerant drug seller<br><br>Other, specify:________	
HM20	Where did the money come from to pay for transportation and medical care for the members of your household who had an illness over the past 30 days? (record all that mentioned)<br><br>*The payment for transportation and medical care can be different. If the respond provided one method, the interviewer should probe and confirm if both payment for transportation and treatment are from the same method.*	Subsidy or exemption→end of the section<br><br>Insurance scheme→ end of the section<br>Mutual Health Organization/Community-Based Health Insurance<br>Health Insurance through Employer<br>Social Security<br><br><br>Out of pocket → HM21<br>Wage/income	

For peer review only - http://bmjopen.bmj.com/site/about/guidelines.xhtml

	If any out-of-pocket method is recorded, proceed to HM21.<br><br>[Insert locally relevant insurance schemes]	Loan/borrowed money<br>Sale of assets<br>Gift from relatives/neighbours/friends<br>Saving/ family funds<br>specify__________--> End of the household head section<br>Don't know → End of the household head section	
HM21	How much in total was spent on [NAME]'s treatment at (name of the place)?	_______amount of money<br>Don't know	If HM20 checked any out of pocket payment.
HM22	How much in total was spent on transport to go to and return from (name of the place)?	_____amount of money<br>Don't know<br><br>➔ HM23, if HM20=loan/borrowed money.<br>➔ Else, end of the household head section	HM23, if HM20=loan/borrowed money.<br>Else, end of the household head section
HM23	Have this debt been repaid?	Yes<br>No<br>Don't know	If HM20=Loan/borrowed money

For peer review only - http://bmjopen.bmj.com/site/about/guidelines.xhtml
For peer review only

	Adult section		
Code	Question	Answers	Skip logic
	**Respondent background**		
A1	How old were you at your last birthday?	Age in complete years __	
A2	Have you ever attended school?	Yes<br>No → A4	If no, then A4
A3	What is the highest level of school you completed?	No formal schooling<br>Less than primary school<br>Primary school uncompleted<br>Primary school completed<br>Secondary school completed<br>High school/ vocational school completed<br>College/university completed<br>Postgraduate degree	
A4	What is your marital status?	Never married<br>Currently married<br>Separated<br>Divorced<br>Widowed<br>Cohabitating<br>Refuse to answer	

For peer review only

For peer review only - http://bmjopen.bmj.com/site/about/guidelines.xhtml

A5	Which of the following best describes your main work status over the past 12 months?	Government employee<br>Non-government employee<br>State enterprise officer<br>Private company employee<br><br>Business<br>Business owner<br>Household business assistance<br>Agriculture<br>Agriculture (landowner and farmer)<br>Agriculture labourer (other's land)<br>Labourer<br>Industrial labourer<br>Day labourer<br>Transport labourer<br>Paid domestic worker<br>Blacksmith/ Goldsmith/other skilled labour<br>Other self-employed<br>Student<br>Home maker/household work<br>Retired<br>Unemployed<br>Unemployed (able to work, e.g. unwilling to work)<br>Unemployed (unable to work, e.g. too sick, disabled)<br><br>Other, specify:_________<br>Refuse to answer	
	Now, I'm going to ask you some questions about your behaviour when you are in or around your household, and when you are working, in the past 12 months.		
ST3	In the past 12 months, how often do you sit or lying directly on the floor in your house or lawn?	Everyday (7 days/week)<br>Almost everyday (5-6 days/week)	

For peer review only - http://bmjopen.bmj.com/site/about/guidelines.xhtml
For peer review only

		Every other day (3-4 days/week)<br>Weekly (1-2 days/week)<br>Monthly (1-3 days/month)<br>Scarcely (a few times/ year)<br>Never	
ST4	In the past 12 months, have you worked in forest or high hill (for collecting wood/ bamboo/ mushroom), or rice field, fruit/tea/coffee plantation, or dry farm field for other agriculture products (e.g. vegetable, corn, pineapple)?<br><br>*Prompt: the participant might have worked in different areas throughout the year, and worked different length in different area, prompt to record all areas that the participate have worked in the past 12 months.*	Yes<br>No→WHS1	If no, then WHS1
ST5	Which one of them have you worked in?<br>*Check all that apply*	Forest or high hill<br>Rice field<br>Fruit/Coffee/tea plantation<br>Dry field	Completing ST6-ST8 for each area checked
ST6	Do you work in the [areas checked above] all year or only when in some seasons?<br><br>*Check "all year" if worked every month in the past 12 months. The respondent might work with different frequency in different month, but if he/she has worked in the area in each month in the past 12 month, this option should be checked.*<br>*Check "in season" if not every month in the past 12 month.*	All year→ST8<br>In season →ST7	

For peer review only - http://bmjopen.bmj.com/site/about/guidelines.xhtml

ST7	How many months have you worked in [area] in the past 12 months?<br><br>*The number of months should be cumulative including all the months in the past 12months the respondent has worked in the area. E.g. if the participants worked in March-June, and November, the total months he/she worked in the area is 4 months, "3-5 months" should be checked.*	9-11 months<br>6-8 months/year<br>3-5 months/year<br>1-2 months/year or less	IfST6= In season
ST8	How often do you work in [area] (when it was the months you worked there)?<br><br>*Ask the question accordingly: if the respondent answered only work in the area in some seasons, then read the words in "(when it was the months you worked there)". If different frequency in different month, please use an average.*	Everyday (7 days/week)<br>Almost everyday (5-6 days/week)<br>Every other day (3-4 days/week)<br>Weekly (1-2 days/week)<br>Monthly (1-3 days/month)<br>Scarcely (a few times/ year)<br><br>➔ ST6, until completed all the areas the participants have listed	ST6 until completed the frequency for all the fields the respondent has worked in the past 12 months.
ST9a	The following questions we will ask you some things you do when you were at work in the area(s) you mentioned before. When you answer them, you need to consider: (all)the area(s) you have mentioned, and the total time you have worked in those area(s) in the past 12 months.<br><br>*Repeat the area(s) and the frequencies of working in the area from ST5-ST8*	Show the area(s) and frequency from ST5-ST8	
ST9	At the time when you work in the area(s), how often do you sit or lay directly on the ground, haystacks, grass or other places in the forest or high hill, or farm field without a mat?	Every time<br>Most times<br>Sometimes<br>Rarely<br>Never	

For peer review only - http://bmjopen.bmj.com/site/about/guidelines.xhtml

ST10	How often do you wear long sleeves?	Every time<br>Most times<br>Sometimes<br>Rarely<br>Never	
ST11	How often do you wear gloves?	Every time<br>Most of times<br>Sometimes<br>Rarely<br>Never	
ST12	How often do you wear shoes with socks or boots without or without socks?	Every time<br>Most of times<br>Sometimes<br>Rarely<br>Never	
ST13	How often do you wash your body after work?	Every time<br>Most of times<br>Sometimes<br>Rarely<br>Never	
ST14	How often do you change your clothes wore at work to clean clothes <u>immediately (i.e., within 30 minutes)</u> when you arrive home?	Every time<br>Most of times<br>Sometimes<br>Rarely<br>Never	
WHS1	I'm going to ask you now your overall health, including both your physical and your mental health.<br><br>In general, how would you rate your health <u>today</u>? Would you say it's very good, good, moderate, bad, or very bad?	Very good<br>Good<br>Moderate<br>Bad<br>Very Bad	

For peer review only - http://bmjopen.bmj.com/site/about/guidelines.xhtml

For peer review only

WHS2	The following questions, I'm going to ask you how much difficulty you have when doing some activities. You can give me answers, such as you have no difficulty, mild difficulty, moderate difficulty, or extreme difficulty, or cannot do it at all.<br><br>Overall in the last 30 days, how much difficulty did you have with work or household activities?	None<br>Mild<br>Moderate<br>Severe<br>Extreme/ Cannot do	
WHS3	Overall in the last 30 days, how much difficulty did you have with moving around, such as walking or climbing steps	None<br>Mild<br>Moderate<br>Severe<br>Extreme/ Cannot do	
WHS4	Overall in the last 30 days, how much difficulty did you have with selfcare, such as washing or dressing yourself?	None<br>Mild<br>Moderate<br>Severe<br>Extreme/ Cannot do	
WHS5	Overall in the last 30 days, how much of bodily aches or pains did you have?	None<br>Mild<br>Moderate<br>Severe<br>Extreme	
WHS6	Overall in the last 30 days, how much difficulty did you have with concentrating or remembering things?	None<br>Mild<br>Moderate<br>Severe<br>Extreme	
WHS7	Do you wear glasses or contact lenses?	Yes<br>No	

For peer review only - http://bmjopen.bmj.com/site/about/guidelines.xhtml
For peer review only

WHS8	In the last 30 days, how much difficulty did you have in seeing (IF WHS7=Yes, even when you wear glasses or contact lenses)?	None<br>Mild<br>Moderate<br>Severe<br>Extreme	
WHS9	Overall in the last 30 days, how much of a problem did you have with sleeping, such as falling asleep, waking up frequently during the night or<br>waking up too early in the morning?	None<br>Mild<br>Moderate<br>Severe<br>Extreme	
WHS10	Overall in the last 30 days, how much of a problem did you have with feeling sad, low, or depressed?	None<br>Mild<br>Moderate<br>Severe<br>Extreme	
	**Substance use**		
SU1	Now I would like to ask you some questions on smoking and tobacco use.<br>Do you currently smoke any tobacco products, such as cigarettes, cigar, or pipes?	Yes<br>No→S6	If No, then S6
SU2	Do you currently smoke tobacco product daily?	Yes<br>No	
SU3	How old were you when you first started smoking?	Age ______years→ S5<br>Don't know→ S4	If S3=Don't know, then S4, else S5
SU4	Do you remember how long ago it was?	__________in year/month/week<br>Don't know	
SU5	What type of tobacco do you currently smoke?<br>(Check all that apply)	Manufactured cigarettes<br>Hand-rolled cigarettes<br>Kreteks<br>Pipes full of tobacco<br>Cigars, cheroots, or cigarillos<br>Water pipe (e.g. Baraku/ Hookah/ Shisha)<br>Other, specify:____	Jump to S10

For peer review only - http://bmjopen.bmj.com/site/about/guidelines.xhtml
For peer review only

		➔ Select any option, move to S10	
SU6	In the past, did you ever smoke any tobacco products?	Yes<br>No→ S10	If S6=No, then S10
SU7	In the past, did you ever smoke tobacco product daily?	Yes<br>No	
SU8	How old were you when you first stopped smoking	Age ______years→S10<br>Don't know→S9	If S8=Don't know, then S9, else S10
SU9	Do you remember how long ago it was?	__________in year/month/week<br>Don't know	
SU10	Now I would like to ask you some questions about drinking alcohol.<br>Have you ever consumed any alcohol, such as beer, wine, spirits, or [ADD OTHER LOCAL EXAMPLES]?	Yes<br>No→S18	If No, then S18
SU11	We count one drink of alcohol as one can or small bottle of beer, one glass of wine, one shot of spirits, or [insert local example].<br><br>(Show card)<br><br>During the past 12 months, how frequently have you had at least one standard alcoholic drink?<br><br>*Refer to locally relevant examples of one standard drink.*	Daily<br>5-6 days per week<br>3-4 days per week<br>1-2 days per week<br>1-3 days per month<br>Less than once a month<br>Never	If S11=Never, then AS18
SU12	Have you consumed any alcohol in the past 30 days?	Yes<br>No→S18	If S12=No then S18
SU13	During the past 30 days, on how many occasions did you have at least one standard alcoholic drink?	Number_____<br>Don't know	

For peer review only - http://bmjopen.bmj.com/site/about/guidelines.xhtml

SU14	In the past 30 days, how many standard drinks on average did you have during one drinking occasion?<br><br>(Showcard)<br>*If the respondent is not able to reply by standard drink, ask on average the amount and type of alcohol he/she consume, and convert by yourself to number of standard drinks.*	Number of drinks_____<br>Don't know	
SU15	In the past 30 days, how many occasions did you have six or more standard alcoholic drinks in a single drinking occasion?<br><br>(Showcard)<br><br>*Prompt: Six standard drinks is roughly about: 6 cans of beer/3 big bottles of beer/ 6 shots of spirit/ a bottle of spirit shared among 3-4 people [insert local examples].*	Number of occasions____<br>Don't know	
SU16	In the past 30 days, did you consume any home brewed alcohol, any alcohol brought over the border/from another country, any alcohol not intended for drinking or other untaxed alcohol?	Yes<br>No→S18	If S16=No then S18
SU17	How many occasions did you consume any home brewed alcohol, any alcohol brought over the border/from another country, any alcohol not intended for drinking or other untaxed alcohol?	Number of occasions___<br>Don't know	
SU18	Do you currently chew betel nuts?	Yes<br>No	If S18=No then S20
SU19	In the last 24 hours, many pieces did you chew?	___pieces	
SU20	Have your ever used heroin, Yama/ice, Ecstasy, Glue, Marijuana, Cocaine, Histamine, Pantazogon, Valium/diazepam, [locally popular drug]?	Yes<br>No<br>Refuse to answer	If No or refuse to answer then D1

For peer review only - http://bmjopen.bmj.com/site/about/guidelines.xhtml

SU21	In the past 12 months have you used any of the drugs?	Yes<br>No<br>Refuse to answer	
S22	Have you ever injected drugs?	No<br>Yes, in past 12 months<br>Yes, before past 12 months<br>Refuse to answer	
	**Disease and symptom**		
D1	Have you ever been diagnosed with chronic lung disease (emphysema, bronchitis, COPD) by a doctor or other health worker?	Yes<br>No→D3	If No, then D3
D2	In the last 30 days, have you been taking any medication or other treatment (like oxygen) for it prescribed by a doctor or other health worker?	Yes<br>No	
D3	In the last 12 months, have you had a tuberculosis (TB) test? I mean, has a doctor examined your sputum (taken a sample of the substance spit out from a deep cough and sent it to a laboratory for analysis) or made an x-ray of your chest?	Yes<br>No→D5	If no, then D5
D4	Have you been taking any medication or treatment during the last 30 days prescribed by a doctor or other health worker?	Yes<br>No	
D5	Have you had fever in the past 30 days?	Yes<br>No→D7	If No, then D7
D6	How long have you had fever?	One week or more<br>No or less than one week	
D7	Are you currently coughing?<br><br>Prompt:<br>If participants answer induced coughing due to environmental factor or substance use, prompt: I mean	Yes<br>No→10	If No, then D10

For peer review only - http://bmjopen.bmj.com/site/about/guidelines.xhtml

	coughing throughout the day, even if you are not smoking or in the environment.		
D8	What is the duration of coughing continuously?	Two weeks or more<br>Less than two weeks	
D9	Are you coughing up blood?	Yes<br>No	
D10	Have you lost body weight unintentional/ suddenly?	Yes<br>No	
D11	In the past 30 days, have you experienced drenching night sweats?	Yes<br>No	If D5-D11 checked 2 or more symptoms, or coughing up blood, then refer to TB care
D12	Have you been diagnosed with Asthma (an allergy respiratory disease) by a doctor or other health worker?	Yes<br>No→D14	If No, then D14
D13	In the last 30 days, have you been taking any medication or treatment prescribed by a doctor or other health worker?	Yes<br>No	
D14	Have you ever had your blood pressure measured by a doctor or other health worker?	Yes<br>No->D17	If No, then D17
D15	Have you ever been told by a doctor or other health worker that you have raised blood pressure or hypertension?	Yes<br>No→D17	If No, then D17
D16	In the past 30 days, have you taken any drugs (medication) for raised blood pressure prescribed by a doctor or other health worker?	Yes<br>No	

For peer review only - http://bmjopen.bmj.com/site/about/guidelines.xhtml

D17	Have you ever had your blood sugar measured by a doctor or other health worker?	Yes<br>No→D20	If No, then D20
D18	Have you ever been told by a doctor or other health worker that you have raised blood sugar or diabetes?	Yes<br>No→D20	If No, then D20
D19	In the past 30 days, have your taken any drugs (medication) or insulin injection for diabetes prescribed by a doctor or other health workers?	Yes<br>No	
D20	Have you ever had your cholesterol (fat levels in your blood) measured by a doctor or other health worker?	Yes<br>No→D23	If No, then D23
D21	Have you ever been told by a doctor or other health worker that you have raised cholesterol?	Yes<br>No→D23	If No, then D23
D22	In the past 30 days, have you taken any oral treatment (medication) for raised total cholesterol prescribed by a doctor or other health worker?	Yes<br>No	
D23	Have you ever had a heart attack or chest pain from heart disease (angina) or a stroke (cerebrovascular accident or incident)?	Yes<br>No	
D24	Are you currently taking medications regularly to prevent or treat heart disease?<br>(for example: aspirin, statins- Lovastatin/ Simvastatin/ Atorvastatin/ any other statin)	Yes<br>No	
D25	Besides the health conditions we have just spoken about, do you currently have other chronic diseases that have been diagnosed by a doctor or health care provider?<br><br>(check all that apply)	No other disease<br>Cancer<br>Liver cancer<br>bronchial and lung cancer<br>Colon and rectal cancer	If mention the disease or symptoms above, go back to the relevant question

For peer review only - http://bmjopen.bmj.com/site/about/guidelines.xhtml
For peer review only

	If diseases that are mentioned are not in the list, but you know in which system it belongs, check "other, specify" in that system and fill the name of the disease.<br><br>*If you do not know which system the disease should be categorized, then check "Other unknow system disease, specify:_______" on the bottom of the list, and fill in the name of the disease.*	Breast cancer<br>Cervical cancer<br>Leukaemia/lymphoma<br>Other, specify:<br>Endocrine system and metabolic disorder<br>thyroid/goitre<br>Other, specify:_________<br>Sensor disorders<br>Glaucoma<br>Cataract<br>Hearing loss<br>Other, specify<br><br>Cardiovascular / circulatory disease<br>Enlarged heart<br>congestive heart<br>heart fluttering<br>Cardiac arteriosclerosis<br>Severe cerebrovascular disease<br>Other, specify:____<br><br>Haematological disease<br>Thalassemia<br>Chronic respiratory disease<br>Chronic emphysema<br>Other, specify:______________<br><br>Digestive disorders<br>Gastritis<br>Acid reflux<br>Liver cirrhosis<br>Other, specify:______<br><br>Genito-urinary disease	

For peer review only - http://bmjopen.bmj.com/site/about/guidelines.xhtml

		Kidney failure/kidney disabilities<br>enlarged prostate/inflammation<br>kidney stone/ bladder stone<br>Other, specify:___________<br><br><u>Muscular-skeleton disease</u><br>Gout<br>rheumatoid/ rheumatoid arthritis<br>Chronic knee pain / chronic back pain/ chronic neck pain<br>Paralysis<br>Other, specify:___________<br><br><u>Chronic infection or defective by infection disease</u><br>HIV<br>Polio<br>Leprosy<br>Chronic HBV infection<br>Chronic HCV infection<br>Other, specify:___________<br><br><u>Congenital anomalies</u><br>abnormal intelligence (Down syndrome)<br>Other, specify:___________<br><br><u>Oral disorders</u><br>Periodontitis (gingivitis)<br>Other, specify:___________<br><br><u>Immune system disorder</u><br>Allergy<br>Other, specify:___________	

For peer review only - http://bmjopen.bmj.com/site/about/guidelines.xhtml
For peer review only

		Neurological disorder<br>Parkinson's disease<br>Epilepsy/epilepsy<br>Dementia<br>Other, specify:__________<br><br>Mental health disorder<br>Alcoholism<br>Depression<br>Other, specify:_________<br><br><br>Other unknow system disease, specify:_______<br><br>Unknown ______	
CC0	You have told us the symptoms and diseases affect your health currently.<br>Now I'd like to ask you about the concerns you have related with health. It can be from the experience of yourself, your family, or your community.<br>I'm going to ask your concerns from different perspective of health, and in the end, I will ask you to rank the ones that concern you the most.		
CC1	The first 3 questions about different dimensions of health services.<br><br>I'm going to ask first if you have concerns about accessing health services, which means the service is provided somewhere, but you cannot receive it<br>Do you have any concerns about health service accessibility?	- Affordability- Cost of care<br>- Geographic accessibility- Travel to service provider (too far, take too much time, no good transportation methods, too sick to travel…)<br>- Language barrier<br>- Culture barrier<br>- Other, specify___________<br>- No concern	

For peer review only - http://bmjopen.bmj.com/site/about/guidelines.xhtml

	Prompt:<br>If the participant said no concern, then prompt service accessibility can be the cost, going to the place, or problem with communication, or something related with culture that create problem for people to get health care.<br><br>*Please record all that the participant mentioned.*		
CC2	The next question is about concerns of health service availability, which means the service is not provided, or some elements required for providing the service is absent or not enough<br>Do you have any concerns related with heath service availability?<br><br>Prompt:<br>If the participant expressed there is no concern, prompt- is there some health care service they want to use but cannot find the service? Or the service is not fully available because of health facility, health care professionals, equipment- laboratory, bed, ultrasound, or medication are absent or not enough.<br><br>*Please record all that the participant mentioned.*	- Physical presence of service (the service is not provided, or not provided all the time or at all. E.g. no dental care in the primary care unit)<br>- Health facility (health centre or hospital)<br>- Health care professionals (doctors/nurses/paramedical professionals)<br>- Beds or equipment<br>- Medication<br>- Other, specify:______<br>- No concern	
CC3	The next question is about your concerns on quality of health service, which means the service is available, and you are able to receive the service, but you are not satisfied with the service<br>Do you have concerns in the quality of health service?<br><br>Prompt:	- Safety: quality care should avoid harms to people whom the care is intended<br>- Timely: waiting time and delays<br>- Efficient: maximize the be benefit of available resources<br>- Other, specify______<br>- No concern	

For peer review only

For peer review only - http://bmjopen.bmj.com/site/about/guidelines.xhtml

	If the participant expressed no concern, prompt it can be related with safety, timely, or efficiency of the service provided.<br><br>*Please record all that the participant mentioned.*		
CC4	We have spoken about concerns related with health services, now Is like you to think about the environment around your household or at work that can potentially harm yours or others' health.<br><br>Do you have concerns related with your house, around your house, or things/ animals belong to your household, that could harm your health or other's health?<br><br>Prompt:<br>If the participant expressed no concern, prompt- it can be part of the structure of the house like wall, floor material; or something inside of the house related with health like toilet, drinking water source; or animals the household own; in the yard, garden of the household.<br><br>*Please record all that the participant mentioned.*	- House (e.g. wall, roof, floor)<br>- Amenities (e.g. toilet, cooking stove, mosquito net, drinking water)<br>- Animals (e.g. free range pigs in the yard)<br>- Other factors in and around the house, specify______<br>- No concern	
CC5	Do you think there are some risks to your or others' health related with work?<br><br>Prompt:<br>If the participant expressed no concern, prompt: for example, you often risk to get injured when you work, certain things/ products used at your work can harm your health.<br>If you work in the forest or in the rice farm, there are some insects or wild animal can harm you too.	- Risk to physical injuries<br>- Risk of chemical exposures<br>- Risk of exposure to nature environment (e.g. mosquito, snake bite)<br>- Other work-related risk factors, specify_________<br>- No concern	

For peer review only - http://bmjopen.bmj.com/site/about/guidelines.xhtml

	Please record all that the participant mentioned.		
CC6	Do you have other concerns about health of yours or people around you?<br>*Please record all that the participant mentioned.*	Presence of disease or symptoms (respondent's or other people)<br>Other, specify________<br>No other concerns	
CC7	Thank you very much for sharing your concerns. You have told me your concerns are: [show results of CC1-CC6]<br>Now, could you (pick the biggest 3 concerns and) rank them? Start from the biggest concern.<br><br>*If the participant expressed less than 3 concerns, then rank the concern(s) mentioned, and select "NA" in the unused field.*	**Top1:________**<br>**Top2:________**<br>**Top3:________**	Automatic showing a list of options selected for CC1-CC6, and pick from the options.

For peer review only - http://bmjopen.bmj.com/site/about/guidelines.xhtml

	Adult women section		
Code	Question	Answers	Skip logic
W1	Now I would like to talk about family planning - the various ways or methods that a couple can use to delay or avoid a pregnancy. Have you ever heard of (METHOD)? (Check all that applied)	Condom<br>Pill<br>IUD<br>Injectables<br>Implants<br>Female condom<br>Emergency contraception<br>Standard days method<br>Lactational amenorrhea method<br>Rhythm method<br>Withdrawal<br>Female sterilization<br>Male sterilization<br>Other modern method, specify: ______<br>Other traditional method, Specify: ___________<br>None<br>Refuse to answer	
W2	Are you currently pregnant?	Yes→W5<br>No or unsure	If yes, W5
W3	Are you or your partner currently doing something or using any method to delay or avoid getting pregnant?<br><br>Prompt: are you or your partner doing any of the following to avoid pregnancy: deliberately avoiding sex on certain days, using a condom, using withdrawal or using emergency contraception?	Yes<br>No→W5<br>Refuse to answer→W5	If No or Refuse to answer, then W5
W4	What are you currently using?	None	

For peer review only - http://bmjopen.bmj.com/site/about/guidelines.xhtml

	**<u>Do not read the answer, check all that applied.</u>**	Female sterilization<br>Male sterilization<br>IUD<br>Injectables<br>Implants<br>Pill<br>Condom<br>Female condom<br>Emergency contraception<br>Standard days method<br>Lactational amenorrhea method<br>Rhythm method<br>Withdrawal<br>Other modern method, specify:____<br>Other traditional method, specify:____	
W5	Now I would like to ask about all the birth you have had during your life. Have you ever given birth?<br>(if No prompt: including the times that a child was born alive but later died, or born dead)	Yes<br>No→ End of the section	If No, then end of the section.
W6	Now I would like to ask some questions about your last birth		
W7	How long ago it was?	_______years →W9<br>Don't know→W8	
W8	How old is the child now?	Age _____years<br>Don't know	If W7=don't know
W9	Did you see anyone for antenatal care for this pregnancy?	Yes<br>No→W12	If No, then W12
W10	Where did you receive antenatal care for this pregnancy?<br><br>Anywhere else?	<u>Home</u><br>Her home→W11<br>Other home→W11<br><br><u>Public sector →W12</u>	If W10= Home/ others then W11

For peer review only - http://bmjopen.bmj.com/site/about/guidelines.xhtml

	Check all that apply<br><br>[Insert locally relevant list of health facilities]	Government hospital<br>Government health centre<br>Government health post<br>Other public sector<br><br>Private medical sector –>W12<br>Private hospitalPrivate clinic<br>Other private medical facility<br><br>NGO medical sector→ W12<br>NGO hospital<br>NGO clinic<br>Other NGO medical<br><br>Other, specify: ______-->W11	
W11	Whom did you see when you received the antenatal care at [her home/ other home]? Anyone else?<br><br>*Check all that apply*<br>Prompt: probe to have information on the health care worker's professional background. Distinguish among different health care professionals.<br><br>[insert locally relevant list]	Health personnel<br>Doctor<br>Nurse/midwife<br>Auxiliary midwife<br><br>Other person<br>Traditional birth attendant<br>Community health worker/<br>Field worker<br><br>Other, specify: __________	
W12	Where did you give birth to this child?	Home<br>Her home →W13<br>Other home→W13<br><br>Public sector →End of the section<br>Government hospital	

For peer review only - http://bmjopen.bmj.com/site/about/guidelines.xhtml

		Government health centre<br>Government health post<br>Other public sector<br><br><u>Private medical sector → End of the section</u><br>Private hospital<br>Private clinic<br>Other private medical sector<br><br><u>NGO medical sector</u><br>NGO hospital<br>NGO clinic<br>Other NGO medical<br><u>Other, specify: ______</u> -->W13	
W13	Who assisted with the delivery?<br><br>Prompt: probe to have information on the health care worker's professional background. Distinguish among different health care professionals.<br><br>Check all that apply	<u>Health personnel</u><br>Doctor<br>Nurse/midwife<br>Auxiliary midwife<br><br><u>Other person</u><br>Traditional birth attendant<br>Community health worker/<br>Field worker<br><br><u>Other, specify: ________</u>	If W12= Home

	Children's section		

For peer review only - http://bmjopen.bmj.com/site/about/guidelines.xhtml

Code	Question	Answers	Skip logic
C1	We would like to ask you question about the [NAME]' health, including breastfeeding, vaccination history.<br>Are you willing to answer the questions?<br>Do you think you are able to answer the questions?	No, I don't want to/ or cannot answer the questions → C2<br>Yes, I'm willing to and able to answer the questions→C3	If No, then C2
C2	Could you tell us who is the best person to answer the questions about [Name] health?	Identify the member list, and ID	Restart from C1 with the person pointed
C3	Relationship of the person answering the question to the child	Mother<br>Father<br>Adopted/foster/ stepmother<br>adopted/foster/ stepfather<br>Grandmother<br>Grandfather<br>Sister<br>Brother<br>Other, specify:_______	
C4	Has [NAME] ever been breastfed?	Yes<br>No→C7	If No, then C7
C5	Is [NAME] still breastfed?	Yes<br>No→7	If No, then C7
C6	Did (NAME) drink or eat anything other than breast milk yesterday or last night? Including plain water, other milk, non-milk liquid, or complementary food)	Yes<br>No	
	**Vaccination history**		**Only ask if 0-35 months child→ C7-C17, else C18**
C7	Now I would like to ask some questions about vaccinations received by [NAME].<br><br>Do you have a card or other document where (NAME)'s vaccinations are written down?	Yes, has only a card<br>Yes, has only another document<br>Yes, has card and other document<br>No, no card and no other document→C15	

For peer review only - http://bmjopen.bmj.com/site/about/guidelines.xhtml
For peer review only

C8	May I see the card or other document where (NAME)'s vaccinations are written down?	Yes, only card seen<br>Yes, only other document seen<br>Yes, card and other document seen<br>No card and no other document seen→C15	If NO then C16
C9	*Don't need to ask the question, compare the record in the vaccination card or document with vaccination schedule of Thailand. Check appropriate vaccines according to the child's age, and choose the option.*<br><br>*(show card)*<br>*Use the vaccination schedule in the show card to compare with the child's vaccination card*	Completed all age-appropriate vaccines→C12<br>Missing 1 dose age-appropriate vaccine<br>Missing more doses age-appropriate vaccines	If completed all age-appropriate vaccines, then C12
C10	Were age-appropriate doses of hepatitis B vaccination given according to the vaccination card or other document?	Yes, completed all age-appropriate doses – C12<br>Received the birth dose, but some doses missing according to children's age →C12<br>There is no record showing hepatitis B vaccination was given	
C11	At or soon after birth, did (NAME) receive a Hepatitis B vaccination, that is, an injection in the thigh to prevent Hepatitis B?	Yes<br>No<br>Don't know	If C11= There is no record
C12	Was a dose of vitamin A supplement given in the past 6 month according to the vaccination card or other document?	Yes →C14<br>No	
C13	In the past 6 months, did [Name] receive a vitamin A dose. (Show common type of ampules/capsules/syrups)	Yes<br>No<br>Don't know	If C13=No
C14	Ask the respondent for permission to photograph vaccination card or other document where vaccinations are written. If permission is granted, photograph card.	Photograph taken<br>Photograph not taken, permission not received<br>Photograph not taken, other reason<br><br>→ End of the section	

For peer review only - http://bmjopen.bmj.com/site/about/guidelines.xhtml

C15	Did (NAME) ever receive any vaccinations to prevent (NAME) from getting diseases, including vaccinations received in campaigns or immunization days or child health days?	Yes<br>No → C18<br>Don't know	If No/ don't know, then C18
C16	How many times did [Name] receive vaccination?	____times<br>Reported completed all age-appropriate vaccine<br>Don't know	
C17	At or soon after birth, did (NAME) receive a Hepatitis B vaccination, that is, an injection in the thigh to prevent Hepatitis B?	Yes<br>No<br>Don't know	
C18	In the past 6 months, did [Name] received vitamin A dose.<br>Show common type of ampules/capsules/syrups)	Yes<br>No<br>Don't know	All under 5 years

The end of questionnaire

For peer review only

For peer review only - http://bmjopen.bmj.com/site/about/guidelines.xhtml
